# Synthesis of Tunable Fluorescent Imidazole-Fused Heterocycle Dimers

**DOI:** 10.1021/acs.orglett.2c01642

**Published:** 2022-07-13

**Authors:** Qiang Zheng, Xin Li, Katarzyna Kurpiewska, Alexander Dömling

**Affiliations:** † University of Groningen, Department of Drug Design, A. Deusinglaan 1, 9713 AV Groningen, The Netherlands; ‡ Department of Crystal Chemistry and Crystal Physics, Faculty of Chemistry, Jagiellonian University, 30-387 Kraków, Poland

## Abstract

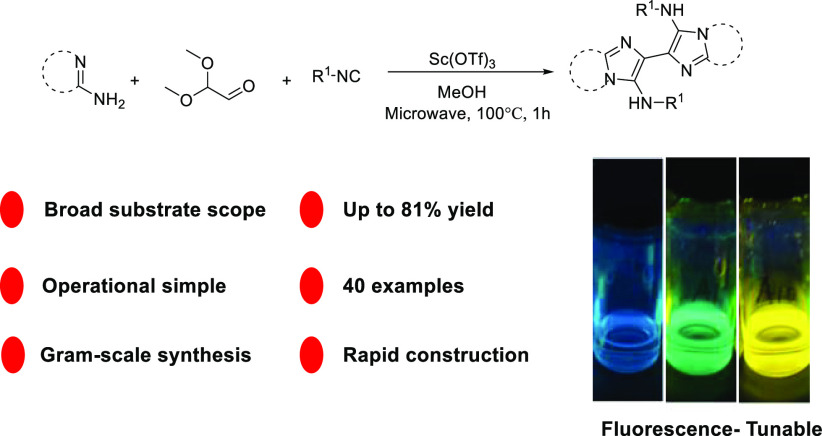

A short, concise, and one-pot synthesis of imidazo-fused heterocycle dimers with tunable fluorescent properties has been developed. By the first time use of glyoxal dimethyl acetal in the Groebke–Blackburn–Bienaymé (GBB) three-component reaction (3CR), the innovation features a new series of fluorescence-tunable imidazo-fused heterocycle dimers exhibiting a broad substrate scope with good yields. Luminescence studies demonstrate that these GBB-dimers possess color-tunable properties, and their emission colors can be successively changed from blue to green and yellow by easy substituent control.

There are many natural and synthetic symmetrical small molecule dimers with potential biological activities, such as anticancer,^1^ antimalarial,^2^ antibacterial,^3^ and opioid antagonist activities.^4,5^ One reason to synthetically aim for symmetrical compounds are homodimeric symmetrical receptors, such as that for PD-L1.^6^ The imidazo[1,2-*a*]heterocyclic scaffold^7^ accessible by the Groebke–Blackburn–Bienaymé (GBB) multicomponent reaction (MCR) is well-known in many FDA-approved drugs, such as miroprofen,^8^ zolpidem,^9^ and DS-1.^10^ The first imidazo[1,2-*a*]pyridine dimer was reported in 2015 by Manna et al. and was produced by the annulation of nitrosopyridine with alkynes (Figure 1).^11^ In 2016, Kudo disclosed the synthesis of noxious organism control agents exploiting the condensation of 2-aminopyridine and imidazo-bromomethyl ketone.^12^ The GBB MCR reaction is an efficient way to get the important imidazo[1,2-*a*]heterocycle scaffold with various aldehydes.^13^ Based on our ongoing interest in MCR chemistry, we described the fast construction of a series of symmetric imidazo[1,2-*a*]heterocycle dimers by one-pot GBB reaction. Importantly, it is the first time that glyoxal dimethyl acetal, which acts as an orthogonal bifunctional monoprotected building block to achieve a new series of fluorescent imidazo[1,2-*a*]heterocycle dimers, was used in the GBB-3CR.

**Figure 1:**
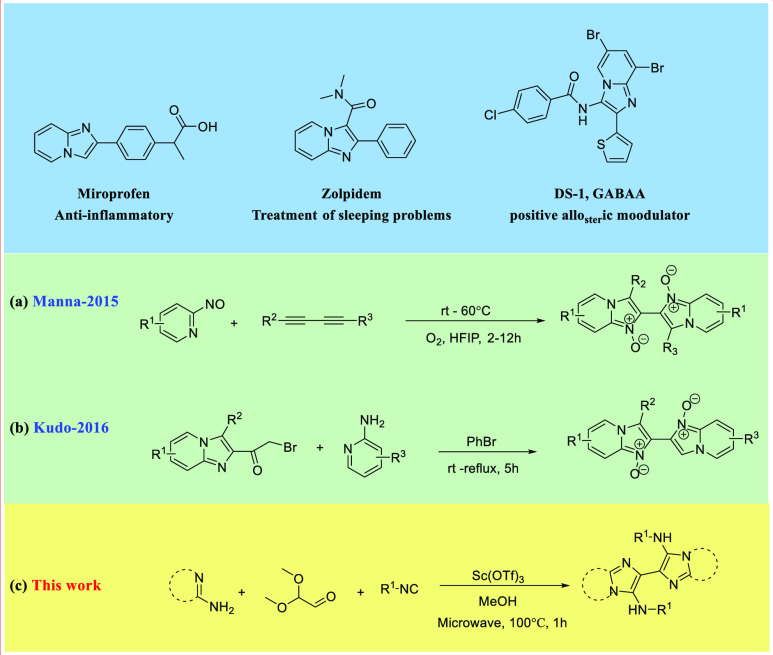
Biological GBB scaffold drugs and previous and current scope of work.

We first selected **1a** and **2a** as model substrates and employed various molar ratios, solvents, and catalysts, and glyoxal dimethyl acetal (60% in H_2_O) for condition optimization (Table 1). First, we screened the ratio of **1a**, glyoxal dimethyl acetal, and **2a** (entries 1–4). We used scandium triflate (Sc(OTf)_3_) as catalyst and methanol as solvent because they are the most often used conditions for GBB-3CR. The reaction with ratio **1a**/glyoxal dimethyl acetal/**2a** at 1:1:1 yielded product in 24% yield, and those with ratios 2:1:2, 2.2:1:2.2, and 1.3:1:1.3 gave 74%, 64%, and 34%, respectively. With the best ratio 2:1:2, we tried to find best solvent for this reaction. The product **3au** was generated in 45% yield when we used trifluoroethanol (TFE) as solvent (entry 5). Water is also a widely used solvent in GBB-3CR; however, it only gave very low yield of 11% (entry 6). The reaction with acetonitrile as solvent gave **3au** in 36% yield, while only 21% and 17% yield were achieved with solvent free reaction and PEG40, respectively (entries 7–9). Subsequently, we surveyed the effects of using different catalysts. When 4-methylbenzenesulfonic acid (PTSA) was employed, 64% yield of product was obtained (entry 5). Inorganic acids such as perchloric acid and acetic acid gave product in 43% and 48% yield, respectively, whereas ammonium chloride could generate **3au** in 51% yield. Zirconium tetrachloride (ZrCl_4_) as Lewis acid could afford the desired product **3a** in 63% yield. Using glyoxal (40 wt % in H_2_O) as the dialdehyde source gave a lower 40% yield. In addition, we also heated this reaction at 80 °C for 12 h, resulting in 54% yield.

**Table 1:**
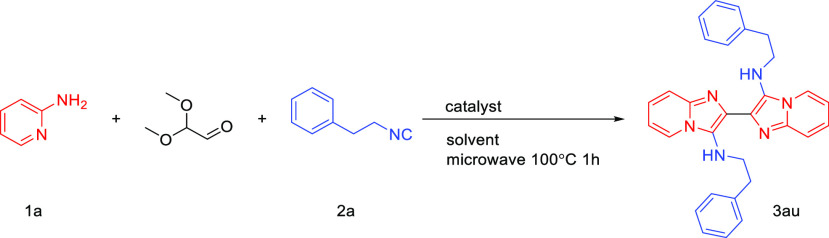
Optimization of Conditions^a^

entry	ratio	solvent	catalyst	yield^b^ (%)
1	1:1:1	MeOH	Sc(OTf)_3_	24
**2**	**2:1:2**	**MeOH**	**Sc(OTf)_3_**	**74**
3	2.2:1:2.2	MeOH	Sc(OTf)_3_	64
4	1.3:1:1.3	MeOH	Sc(OTf)_3_	34
5	2:1:2	TFE	Sc(OTf)_3_	45
6	2:1:2	water	Sc(OTf)_3_	11
7	2:1:2	MeCN	Sc(OTf)_3_	36
8	2:1:2	solvent free	Sc(OTf)_3_	21
9	2:1:2	PEG40	Sc(OTf)_3_	17
10	2:1:2	MeOH	PTSA	64
11	2:1:2	MeOH	perchloric acid	43
12	2:1:2	MeOH	acetic acid	48
13	2:1:2	MeOH	NH_4_Cl	51
14	2:1:2	MeOH	ZrCl_4_	63
15^c^	2:1:2	MeOH	Sc(OTf)_3_	40
16^d^	2:1:2	MeOH	Sc(OTf)_3_	54

a Reaction conditions: unless otherwise stated, all the reactions were performed with **1a** (1 mmol), glyoxal dimethyl acetal (60% in H_2_O, 0.5 mmol), **2a** (1 mmol), and catalyst (20 mol %) in solvent (0.5 mL) at 100 °C under microwave radiation for 1 h. PTSA = 4-methylbenzenesulfonic acid; TFE = trifluoroethanol.

b Isolated yield.

c Glyoxal (40 wt % in H_2_O) was used as dialdehyde source.

d Heated at 80 °C for 12 h in sealed vial using aluminum heating blocks.

Having identified the optimal reaction conditions (Table 1, entry 2), we evaluated the substrate scope of the newly developed synthetic protocol (Scheme 1). When *tert*-butyl isocyanide reacted with substituted 2-aminopyridine with different electron-withdrawing groups, desired products were finally obtained in relatively good yields (**3aa**–**3ad**). Gratifyingly, cyclohexyl isocyanide was able to afford desired product **3ae** in 81% yield. The use of various substituents on the 2-aminopyridine regardless of their electronic characteristics, such as chloro (**3af**), bromo (**3ag**, **3ah**), iodo (**3aj**), trifluoromethyl (**3ak**), methyl (**3al**), and methyl formate (**3am**), afforded the corresponding products in 35–66% yields.

**Scheme 1:**
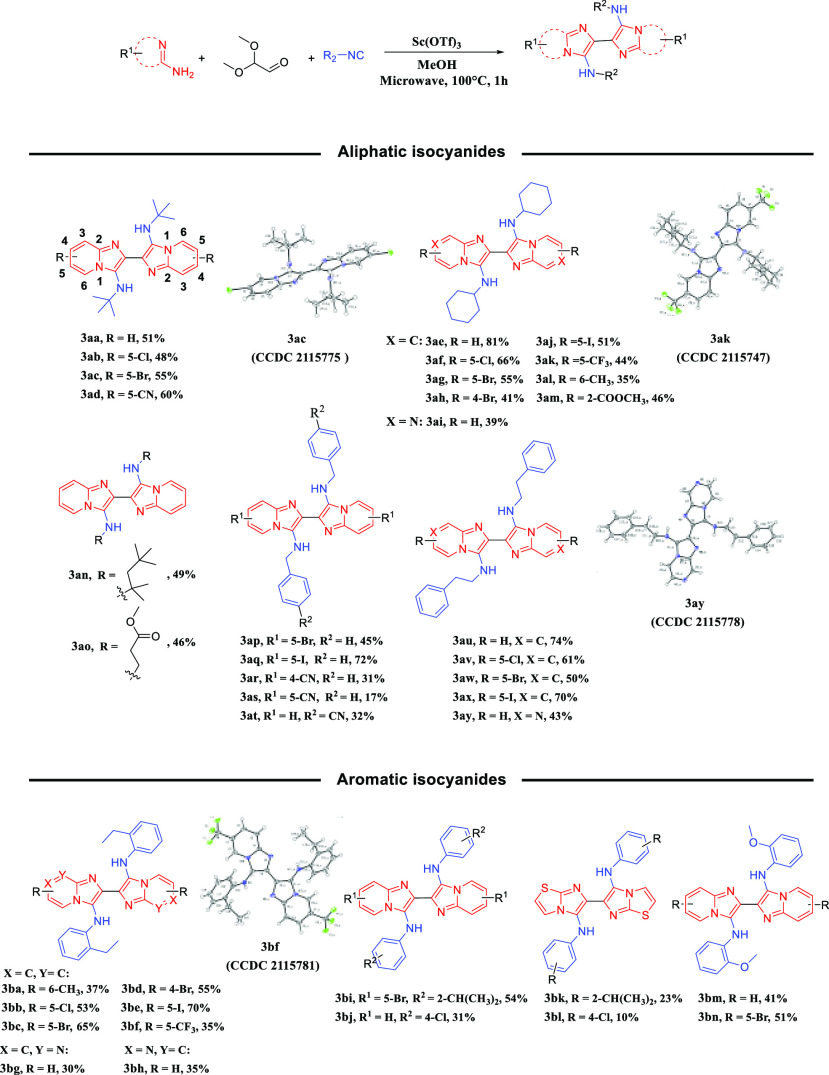
Scope of the Substrates

We also tried pyrimidin-2-amine to add more diversity of the central imidazo-bicycle to the GBB dimer scaffold, and the product was generated in 39% yield (**3ai**). In addition, some other isocyanides such as 2-isocyano-2,4,4-trimethylpentane and methyl 3-isocyanopropionate could also provide products in moderate yields, 49% and 46%, respectively (**3an**–**3ao**). Interestingly, benzyl isocyanide was also tolerated under the current conditions when reacted with bromo or iodo substituted 2-aminopyridine (**3ap**, **3aq**), whereas it only furnished the corresponding products in reduced yields when reacted with cyano substituted 2-aminopyridine (**3ar**, **3as**).

When 4-(isocyanomethyl)benzonitrile was used to react with 2-aminopyridine, product **3at** was afforded only in 32% yield. When phenylethyl isocyanide reacted with substituted 2-aminopyridine with different halogen atoms, the desired products were obtained in good yields (**3au**–**3ay**). Subsequently, we explored the applicability of the reaction conditions to different aromatic isocyanides. Satisfactorily, when phenylethyl isocyanide reacted with substituted 2-aminopyridine with different halogen atoms, desired products were finally obtained in relatively good yields (**3ba**–**3bf**). However, pyrimidin-2-amine and pyrazin-2-amine could only afford the corresponding products with relatively low yields, 30% and 35%, respectively (**3bg**, **3bh**). For aromatic isocyanides such as 1-isocyano-2-isopropylbenzene and 1-chloro-4-isocyanobenzene, compounds **3bi** and **3bj** were obtained in 54% and 31% yield, respectively. To further explore the scope, we used thiazol-2-amine to obtain a unique 6,6′-biimidazo[2,1-*b*]thiazole scaffold of **3ai** and **3aj** with somewhat diminished yield, 23% and 10%, respectively (**3bk**, **3bl**). In addition, 2-methoxyphenyl isocyanide, which was also a suitable substrate, generated corresponding products in moderate to good yields (**3bm**, **3bn**). Significantly, we successfully obtained crystal structures of **3ab**, **3ac**, **3ag**, **3ak**, **3aw**, **3ay**, and **3bf** by X-ray crystallography analysis (Figure S1). Worthwhile to mention, the exocyclic NH of one imidazo ring forms an intramolecular hydrogen bond to the imidazo-N of the next ring, providing a rather rigid fully coplanar hexacyclic ring system, belonging to the symmetry point group *C*_2*h*_, with an inversion center, a 2-fold rotation axis, and a horizontal plane.

To verify the synthetic practicality of this simple workup reaction, we carried out a gram-scale experiment in which the model reaction was performed on a 5 mmol scale; compound **3bc** was isolated in 54% yield (1.69 g) by prolonging the reaction time to 2 h (Scheme 2a). Further application was demonstrated by the hydrolysis of compound **3am** (Scheme 2b). The hydrolysis of **3am** proceeded smoothly, giving the corresponding 3,3′-bis(cyclohexylamino)-[2,2′-biimidazo[1,2-*a*]pyridine]-6,6′-dicarboxylic acid **3bo** with 90% yield.^14^

**Scheme 2:**
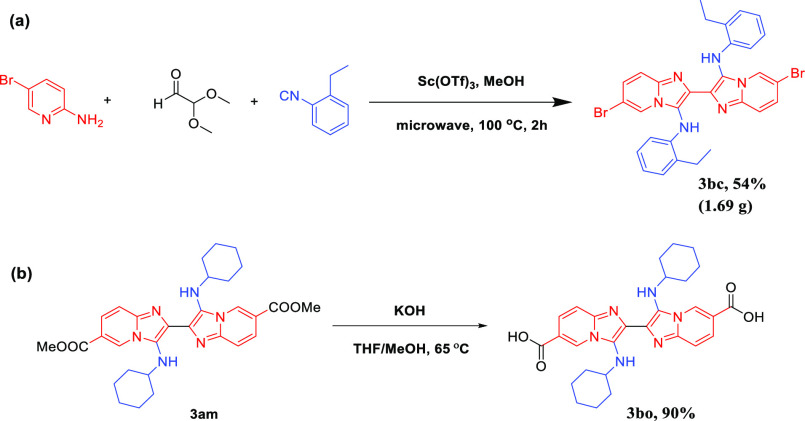
Gram-Scale Synthesis and Applications

In addition to synthesis of symmetric GBB dimers, we also explored unsymmetric synthesis, incorporating two different imidazo heterocycles. As shown in Scheme 3, 5-chloropyridin-2-amine and 4-bromopyridin-2-amine were employed at the same time to afford unsymmetric compound **3bp** in 26% yield, while the two homodimers **3bb** and **3bd** were generated in 40% and 8% yield, respectively. Likewise, another example produced unsymmetric **3bq** in 28% yield and symmetric **3br** and **3bs** in 36% and 8% yield. We obtained the crystal structure of **3bp** (Figure S1).

**Scheme 3:**
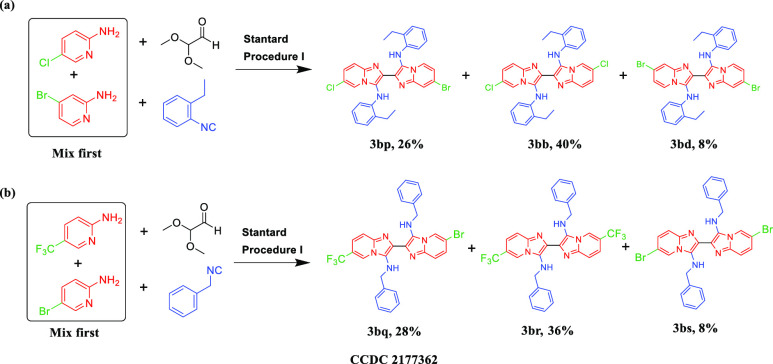
Unsymmetric Synthesis

Then, we investigated the luminescence properties of these GBB dimers. First, we determined a suitable wavelength for the photoluminescence assay by UV/vis spectroscopy. The UV/vis absorption spectrum of **3ae** recorded in different solvents showed that THF is the best solvent to give a strong absorption band centered at 390 nm with another maxima (280 nm) in the ultraviolet region (Figure S2A). Next, we recorded the UV/vis absorption spectra of **3bj**, **3bm**, **3aa**, **3al**, **3ai**, **3ag**, and **3ak** in THF and obtained their maximum absorption wavelengths (Figure S1B). Due to the weak fluorescence intensities achieved from the λ_max_ in the ultraviolet region of these dimers, we chose another λ_max_ in the visible region and tested the luminescence activity of **3ae** with an excitation at 390 nm in different solvents, and THF turned out to be the best solvent, showing the highest luminescence intensity (Figure 2A). Then, we carried out luminescence photophysical studies on further GBB-dimer compounds. With excitation wavelengths at 365 or 370 nm, **3bj** and **3bm**, both containing aromatic amine moieties, emitted a blue luminescence at 455 nm in THF at 25 °C. When arylamino substituents were changed to alkylamino substituents like *tert*-butylamino or cyclohexylamino groups, the emission wavelengths of **3aa** and **3ae** were red-shifted to 460 nm (light blue) or 500 nm (green). Interestingly, when an electron-donating methyl group was introduced on the pyridine ring, compound **3al** could achieved a slight blue shift to 490 nm compared to that of **3ae** (green). The electron-withdrawing nitrogen atom (**3ai**, 520 nm), 5-Br (**3ag**, 530 nm), or 5-CF_3_ (**3ak**, 545 nm) employed with the pyridine resulted in the emission band being red-shifted to the yellow color range. Notably, **3ag** showed a significantly weak fluorescence intensity, suggesting that 5-Br could be used to reduce luminescence activity. The relative fluorescence quantum yields (Φ) of compounds in Figure 2 are also summarized in Table S3 (see Supporting Information).

**Figure 2:**
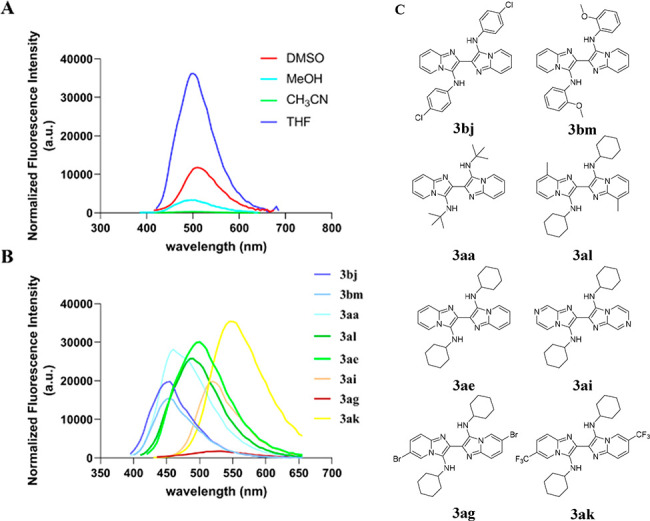
(A) Fluorescence spectra of **3ae** (10 μM) in different solvents at 25 °C with an excitation at 390 nm. (B) Fluoroscence intensity of **3bj**, **3bm**, **3aa**, **3ae**, **3al**, **3ai**, **3ag**, and **3ak** (10 μM) in THF at 25 °C at corresponding excitation. (C) Structures of compounds.

In summary, we have reported a multicomponent reaction of isocyanide, amidine, and glyoxal dimethyl acetal leading to various tunable fluorescence imidazole-fused heterocycle dimers. This method features high synthetic efficiency, mild conditions, operational simplicity, and broad substrate scope. A plausible mechanism has also been proposed in the Supporting Information. Furthermore, these compounds possess color fine-tunable luminescence properties, and we can achieve a sequentially change in the emission colors of these GBB-dimers from blue to green and yellow by introducing corresponding 2-amino pyridines or isocyanides.

## Supporting Information Available

Experimental procedures, ^1^H and ^13^C{^1^H} NMR spectra for all compounds, and X-ray crystallographic data for **3ab**, **3ac**, **3ag**, **3ak**, **3aw**, **3ay**, **3bf**, and **3bq** (PDF)

## Accession Codes

CCDC 2115747, 2115775–2115776, 2115778–2115781, and 2177362 contain the supplementary crystallographic data for this paper. These data can be obtained free of charge via www.ccdc.cam.ac.uk/data_request/cif, or by emailing data_request@ccdc.cam.ac.uk, or by contacting The Cambridge Crystallographic Data Centre, 12 Union Road, Cambridge CB2 1EZ, UK; fax: +44 1223 336033.
