# Supplementary material for: Synthesis of Tunable Fluorescent Imidazole-Fused Heterocycle Dimers
Source: Org Lett. 2022 Jul 13;24(28):5014–7. doi: 10.1021/acs.orglett.2c01642 (PMC9490819; doi:10.1021/acs.orglett.2c01642)
Supplement: Supplementary file 1 — ol2c01642_si_001.pdf [file ol2c01642_si_001.pdf]

Supplementary Information

Synthesis of Tunable Fluorescent Imidazole-fused Heterocycle Dimers

Qiang Zheng[a#], Xin Li[a#], Katarzyna Kurpiewska[b], and Alexander Dömling[a*]

[a]University of Groningen, Department of Drug Design, A. Deusinglaan 1, 9713 AV Groningen, The Netherlands

[b]Department of Crystal Chemistry and Crystal Physics Faculty of Chemistry, Jagiellonian University, 30-387 Kraków, Poland

#These authors contributed equally.

*Corresponding e-mail: a.s.s.domling@rug.nl

Table of contents

1. General information--S2

2. Crystal sample preparation procedure and X-ray Crystallographic Data -----------------S3-S6

3. Figure S2. UV−visible absorption spectrum of compounds in Figure 2----------------------S6

4. Table S2: The absorption maximum wavelengths and molar absorption coefficient-----------S6

5. Table S3: Relative Fluorescence Quantum Yields (Φ) of compounds in Figure 2------------S7

6. General Procedure **I-III**--S8

7. Proposed Mechanism---S9

8. Characterizations of final compounds---S10-S20

9. ^{1}H and ^{13}C NMR spectrums of **3aa-3bq**---S21-S65

10. References---S65

Supplementary Methods

General Information.

Reagents were available from commercial suppliers and used without any purification unless otherwise noted. All isocyanides were made in house by performing the Ugi procedure. Other reagents were purchased from Sigma Aldrich, ABCR, Acros, Fluorochem and AK Scientific and were used without further purification. Nuclear magnetic resonance spectra were recorded on a Bruker Avance 500 spectrometer. Chemical shifts for ^{1}H NMR were reported relative to TMS (δ 0 ppm) or internal solvent peak ($CDCl_3$ δ 7.26 ppm, CD_3OD δ 3.31 ppm or D_2O δ 4.79 ppm) and coupling con-stants were in hertz (Hz). The following abbreviations were used for spin multiplicity: s = singlet, d = doublet, t = triplet, dt = double triplet, ddd = doublet of double doublet, m = multiplet, and br = broad. Chemical shifts for 13C NMR reported in ppm relative to the solvent peak ($CDCl_3$ δ 77.23 ppm, DMSO δ 39.52 ppm, CD_3OD δ 49.00 ppm). Flash chromatography was performed on a Grace Reveleris X2 using Grace Reveleris Silica columns (12g) and a gradient of petroleum ether/ethyl acetate (0–100%) or dichloromethane/methanol (0–20%) was applied. All microwave irradiation reactions were carried out in a Biotage Initiator™ Microwave Synthesizer Thin layer chromatography was performed on Fluka pre-coated silica gel plates (0.20 mm thick, particle size 25 μm). Mass spectra were measured on a Waters Investigator Supercritical Fluid Chromatograph with a 3100 MS Detector (ESI) using a solvent system of methanol and CO_2 on a Viridis silica gel column (4.6 × 250 mm, 5 μm particle size) and reported as (m/z). High resolution mass spectra (HRMS) were recorded using a LTQ-Orbitrap-XL (Thermo Fisher Scientific; ESI pos. mode) at a resolution of 60000@m/z400. UV-absorbance and fluorescence spectra were recorded on a Synergy H1 Hybrid Reader (BioTek) instrument. Melting points were obtained on a melting point apparatus and were uncorrected.

Crystal sample preparation:

Method A: Crystallize directly in an NMR tube with $CDCl_3$.
The sealing film was wrapped around the nozzle of the sample NMR tubes, and several small holes were punched in the sealing film, then the NMR tubes were kept still in a dark environment for 2-4 weeks.

Method B: recrystallization in EtOH
30-50 mg sample powder was added into the 3 mL vial with around 1 mL EtOH, sealed the vial and heated at 70 ^{o}C for sereral minutes, filtered the solution and removed undissolved material, then transfer the filtrate to a new 3 mL glass vial , cap the vial, and kept still for 2-4 weeks in a dark environment.

Crystal structure determination

X-ray diffraction data for single crystal of compound **3ab, 3ac, 3ag, 3ak, 3aw, 3ay, 3bf** and **3bq** was collected using Rigaku XtaLAB Synergy S Dualflex diffractometer (four circle diffractometer with a mirror monochromator) with HyPix detector and a PhotonJet CuKα radiation source (λ = 1.54184 Å) for all collected data sets. Additionally, the diffractometer was equipped with a CryoJet HT cryostat system (Oxford Instruments) allowing low temperature experiments, performed at 100 (11) K. The obtained data set was processed with CrysAlisPro software[1]. The phase problem was solved by direct methods using SUPERFLIP[2]. Parameters of obtained models were refined by full-matrix least-squares on F^2 using SHELXL-2018/3[3]. Calculations were performed using WinGX integrated system (ver. 2021.2)[4]. Figure was prepared with Mercury 2020.3.0 software[5].

All non-hydrogen atoms were refined anisotropically. All hydrogen atoms attached to carbon atoms were positioned with the idealised geometry and refined using the riding model with the isotropic displacement parameter $U_{iso}[H]$ = 1.2 (or 1.5 (methyl groups only)) $U_{eq}[C]$. Crystal data and structure refinement results for presented crystal structure are shown in Table S1. The molecular geometry (asymmetric unit) observed in the crystal structure is shown in Figure S1.

Crystallographic data have been deposited with the Cambridge Crystallographic Data Centre as supplementary publication no. CCDC 2115776 (**3ab**), CCDC 2115775 (**3ac**), CCDC 2115779 (**3ag**), CCDC 2115747 (**3ak**) , CCDC 2115780 (**3aw**), CCDC 2115778 (**3ay**), CCDC 2115781 (**3bf**) and CCDC 2177362 (**3bq**). Copies of the data can be obtained, free of charge, on application to CCDC, 12 Union Road, Cambridge CB2 1EZ, UK, (fax: +44-(0)1223-336033 or e-mail: deposit@ccdc.cam.ac.uk).

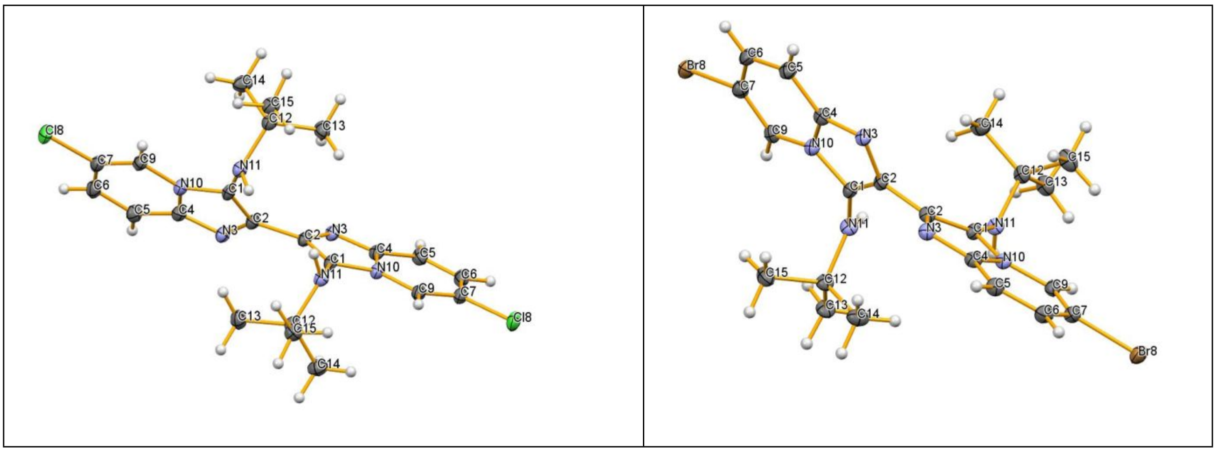

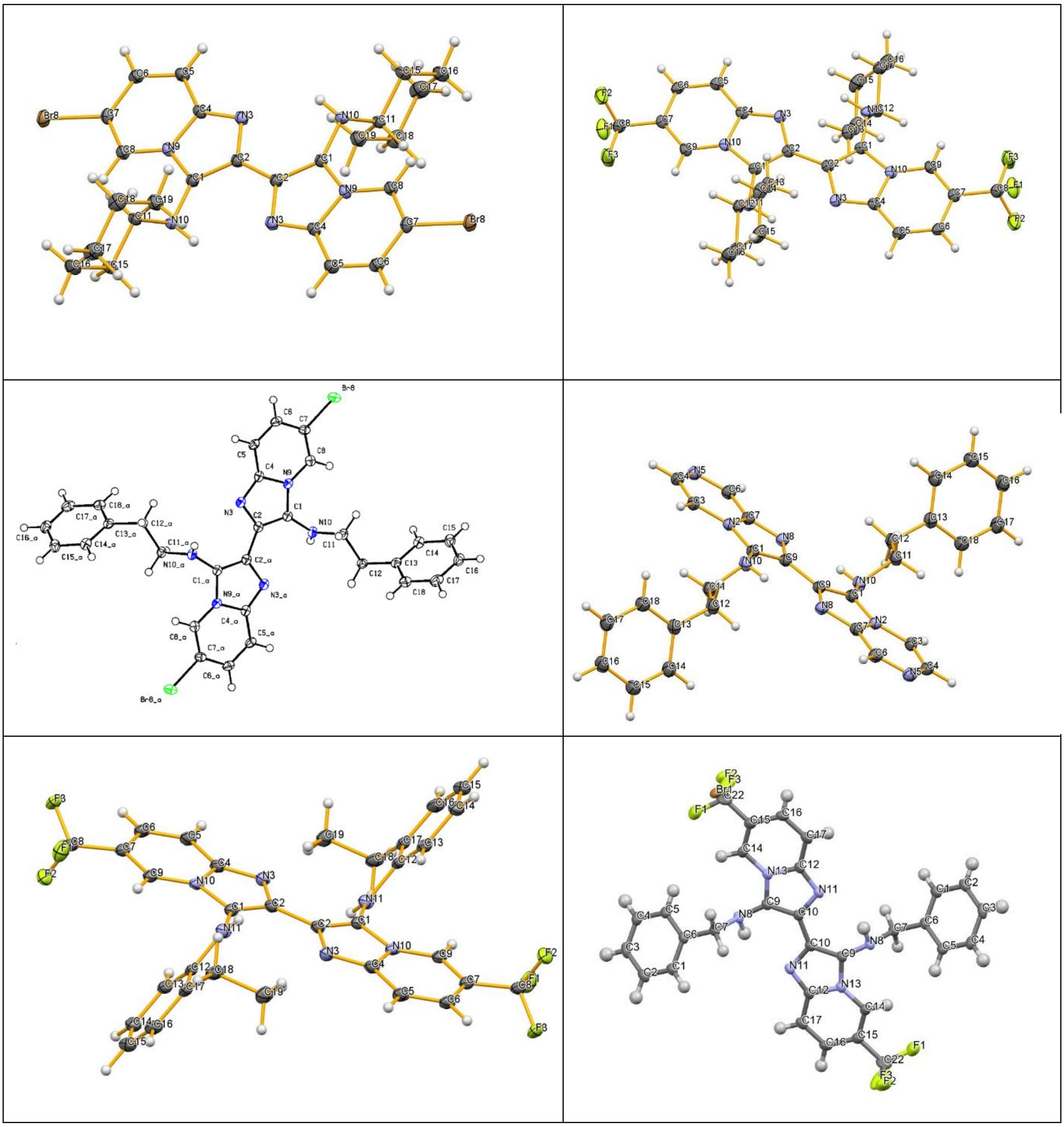

Figure S1. Molecular geometry observed in the crystal structures of compounds **3ab, 3ac, 3ag, 3ak, 3aw, 3ay, 3bf** and **3bq** (asymmetric unit entitles half of the molecules, here shown entire molecules generated by the symmetry operations) showing the atom labelling scheme. The positional disorder within the benzene ring is observed with equal site occupancy (50:50). Displacement ellipsoids of non-hydrogen atoms are drawn at the 30% probability level. H atoms are presented as small spheres with an arbitrary radius.

Table S1. Crystal data and structure refinement results for compounds **3ab, 3ac, 3ag, 3ak, 3aw, 3ay 3bf** and **3bq**.

	**3ab**	**3ac**	**3ag**	**3ak**	**3aw**	**3ay**	**3bf**	**3bq**
Empirical moiety formula	C_{22} H_{26} Cl_2 N_6	C_{22} H_{26} Br_2 N_2	C_{26} H_{30} Br_2 N_6	C_{28} H_{30} F_6 N_6	C_{30} H_{26} Br_2 N_6	C_{28} H_{26} N_8	C_{32} H_{26} F_6 N_6	C_{29} H_{22} Br F_3 N_6
Formula weight [g/mol]	445.39	534.29	586.36	564.58	630.37	474.57	608.59	591.43
Crystal system	Triclinic	Monoclinic	Monoclinic	Monoclinic	Monoclinic	Monoclinic	Triclinic	Monoclinic

Space group	$P\bar{1}$	*C2/c*	*C2/c*	$P2_1/c$	*C2/c*	$P2_1/n$	$P\bar{1}$	*I 2/a*
Unite cell dimensions	a = 6.0868(2) Å b = 9.3555(2)Å c = 10.1126(3) Å α=68.554(2)° β= 87.729(2)° γ=82.093(2)°	a = 20.0848(3) Å b = 6.02270(10) Å c = 19.8516(3) Å α=90° β= 112.855(2)° γ=90°	a = 23.0708(3) Å b = 6.08570(10) Å c = 17.6425(2) Å α=90° β= 97.0390(10) ° γ=90°	a = 12.7587(4)Å b = 5.20290(10)Å c = 20.1059(6)Å α=90° β= 105.252(3)° γ=90°	a = 25.1289(4)Å b = 4.60290(10)Å c = 22.3158(4) Å α=90° β= 98.181(2) ° γ=90°	a = 4.47770(10) Å b = 24.4436(2)Å c = 10.62450(10)Å α=90° β= 90.4190(10)° γ=90°	a = 4.48960(10) Å b = 11.2573(4)) Å c = 14.0120(4)Å α=102.195(3) ° β= 98.109(2) ° γ=96.233(2) °	a = 20.8037(5) Å b = 4.61240(10) Å c = 27.2762(6)) Å α=90° β= 102.747(2)° γ=90°
Volume [Å^3]	530.86(3)	2212.81(7)	2458.37(6)	1287.66(6)	2554.91(8)	1162.83(3)	678.23(4)	2552.78(10)
Z	1	4	4	2	4	2	1	4
D_{calc} [Mg/m^3]	1.393	1.604	1.590	1.456	1.639	1.367	1.490	1.539
μ [mm^{-1}]	2.875	4.814	4.393	1.019	4.282	0.674	1.020	2.656
F(000)	234	1080	1192	588	1272	500	314	1200
Crystal size [mm^3]	0.2 x 0.2 x 0.1	0.2 x 0.15 x 0.1	0.25 x 0.2 x 0.1	0.15 x 0.15 x 0.1	0.3 x 0.3 x 0.2	0.2 x 0.2 x 0.15	0.25 x 0.2 x 0.1	0.43 x 0.06 x 0.03
Θ range	4.69° to 75.40°	4.78° to 74.97°	3.86° to 80.31°	3.59° to 79.87°	3.55° to 80.32°	3.61° to 80.74°	3.27° to 75.05°	3.32° to 75.58°
Index ranges	-7 ≤ h ≤ 7, -11 ≤ k ≤ 11, -12 ≤ l ≤ 12	-24 ≤ h ≤ 24, -7 ≤ k ≤ 7, -24 ≤ l ≤ 24	-29 ≤ h ≤ 28, -7 ≤ k ≤ 6, -22 ≤ l ≤ 22	-16 ≤ h ≤ 16, -6 ≤ k ≤ 6, -25 ≤ l ≤ 24	-31 ≤ h ≤ 30, -5 ≤ k ≤ 5, -26 ≤ l ≤ 28	-4 ≤ h ≤ 5, -31 ≤ k ≤ 31, -13 ≤ l ≤ 13	-5 ≤ h ≤ 5, -13 ≤ k ≤ 14, -16 ≤ l ≤ 17	-25 ≤ h ≤ 26, -4 ≤ k ≤ 5, -31 ≤ l ≤ 34
Refl. collected	12996	24163	7526	15024	7829	35339	12856	12194
Independent reflections	2067 [R(int) = 0.0298]	2204 [R(int) = 0.0623]	2617 [R(int) = 0.0233]	2777 [R(int) = 0.0716]	2693 [R(int) = 0.0324]	2533 [R(int) = 0.0298]	2637 [R(int) = 0.0716]	2551 [R(int) = 0.0301]
Completeness [%] to Θ	99.8 (Θ 67.68°)	99.8 (Θ 67.68°)	99.0 (Θ 67.68°)	98.6 (Θ 67.68°)	96.6 (Θ 67.68°)	99.0 (Θ 67.68°)	99.9 (Θ 67.68°)	99.6 (Θ 67.68°)
Absorption correction	Multi-scan	Multi-scan	Multi-scan	Multi-scan	Multi-scan	Multi-scan	Multi-scan	Multi-scan
Tmin. and Tmax.	0.750 and 1.000	0.426 and 1.000	0.809 and 1.000	0.300 and 1.000	0.590 and 1.000	0.618 and 1.000	0.300 and 1.000	0.675 and 1.000
Data/ restraints/ parameters	2067 / 0 / 144	2204 / 0 / 139	2617 / 0 / 159	2777 / 0 / 186	2693 / 0 / 159	2533 / 0 / 165	2637 / 0 / 200	2551 / 204 / 11
GooF on F2	1.047	1.050	1.069	1.095	1.057	1.059	1.107	1.116
Final R indices [I>2sigma(I)]	R1= 0.0280, wR2= 0.0700	R1= 0.0299, wR2= 0.0788	R1= 0.0262, wR2= 0.0695	R1= 0.0476, wR2= 0.1305	R1= 0.0363, wR2= 0.1001	R1= 0.0477, wR2= 0.1185	R1= 0.0657, wR2= 0.1797	R1= 0.0395,
R indices (all data)	R1= 0.0275, wR2= 0.0703	R1= 0.0293, wR2= 0.0794	R1= 0.0253 wR2= 0.0690	R1= 0.0453 wR2= 0.1266	R1= 0.0353 wR2= 0.0991	R1= 0.0443, wR2= 0.1159	R1= 0.0610 wR2= 0.1745	wR2= 0.1051
$\Delta\rho_{max}$, $\Delta\rho_{min}$ [e·Å^{-3}]	0.36 and -0.25	0.65 and -0.75	0.45 and -0.41	0.39 and -0.30	0.80 and -0.95	0.27 and -0.40	0.47 and -0.59	R1= 0.0386,

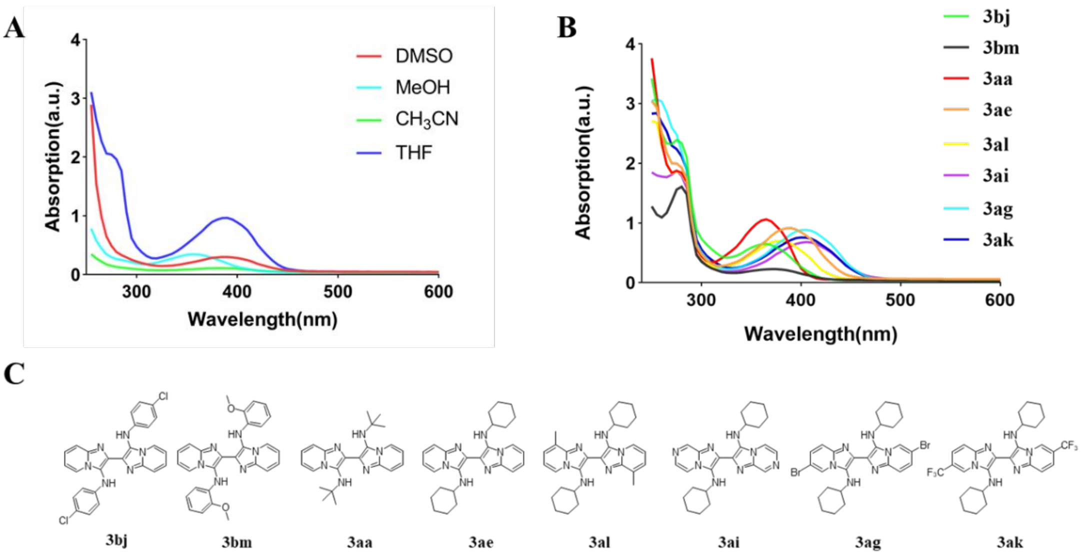

Figure S2. (A) UV−visible absorption spectrum of **3ae** (10 μM) in different solvents; (B) UV−visible absorption spectrum of **3bj**, **3bm**, **3aa**, **3ae**, **3al**, **3ai**, **3ag**, **3ak**, (10 μM) in THF.

Table S2: The absorption maximum wavelengths and molar absorption coefficient

No.	Solvent	Compd.	$\lambda_{abs\ (nm)}$	Molar absorption coefficient ($M^{-1}cm^{-1}$)
1	THF	3ae	390	96300
2	DMSO	3ae	390	29800
3	MeOH	3ae	355	34200
4	CH_3CN	3ae	385	10700
5	THF	3bj	365	65000
6	THF	3bm	370	22800
7	THF	3aa	365	106100
8	THF	3al	380	69700
9	THF	3ai	405	68000
10	THF	3ag	405	88400
11	THF	3ak	400	75800

Table S3: Relative Fluorescence Quantum Yields (Φ) of compounds in Figure 2.

No.	Solvent	Compd.	$\lambda_{em\ (nm)}$	Relative Φ
1	Dioxane	Anthracene	400	0.36[a]
2	THF	Anthracene	405	0.180
3	THF	3ae	500	0.061
4	DMSO	3ae	515	0.071
5	MeOH	3ae	495	0.014
6	CH_3CN	3ae	505	0.004
7	THF	3bj	455	0.050
8	THF	3bm	455	0.110
9	THF	3aa	460	0.043
10	THF	3al	490	0.060
11	THF	3ai	520	0.048
12	THF	3ag	530	0.003
13	THF	3ak	545	0.076

[a]absolute *Φ of* Anthracene in Dioxane[6].

The method of determining quantum yields is the most widely used relative method and the quantum yield of the unknown Φ_X is calculated according to the following equation.

$$\Phi_X = \Phi_R * \frac{A_R}{A_X} * \frac{E_X}{E_R} * \frac{n_X^2}{n_R^2}$$

A is the absorbance of the solution, E is the corrected emission intensity, and n is the average refractive index of the solution. Subscripts R and X refer to the reference and unknown compound, respectively. In our case, the reference compound is Anthracene (Φ_R = 0.36, cyclohexane)[6].

Refractive Index value of solvents are listed below.

n_{THF} = 1.407 n_{DMSO} = 1.479 n_{MeOH} = 1.328 n_{CH3CN} = 1.344 $n_{Dioxane}$ = 1.422

General Procedure I: for synthesis of GBB dimers

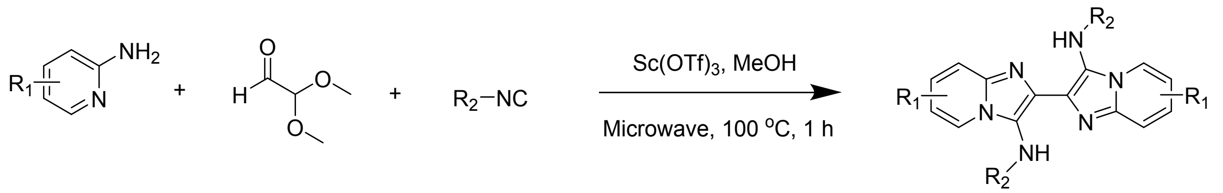

Procedure: To a solution of 2-aminopyridine (1 mmol) and 2,2-dimethoxyacetaldehyde(60 wt. % in H_2O, 0.85 mmol) in MeOH, $Sc(OTf)_3$ (0.1 mmol) and isocyanide (1 mmol) were added into a seal microwave vial. The mixture was heat at 100 °C in microwave for 1 h. After the reaction, in most cases, yellow solid would precipitate in the vial, then the solid was filtrated and dried to get the finally compound; in the cases without precipitates, the purification was conducted by column chromatography to get the final product.

General Procedure II: for synthesis of GBB dimers

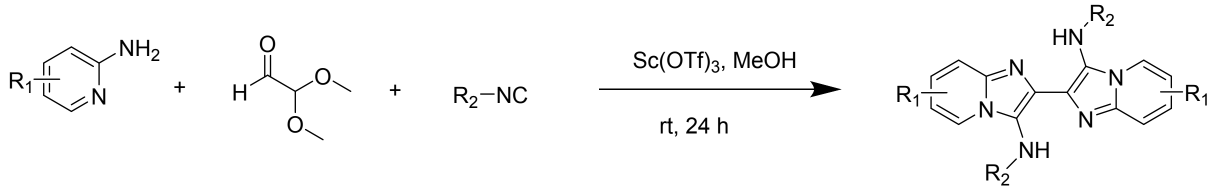

Procedure: To a solution of 2-aminopyridine (1 mmol) and 2,2-dimethoxyacetaldehyde(60 wt. % in H_2O, 0.85 mmol) in MeOH, $Sc(OTf)_3$ (0.1 mmol) and isocyanide (1 mmol) were added into a seal microwave vial. The mixture was stirred at room temperature for 24 h. After the reaction, the purification was conducted by column chromatography to get the final product.

General Procedure III: for hydrolysis of GBB dimer

Procedure: GBB dimer (0.2mmol) was suspended in a mixture of THF/MeOH(1:1, 1ml) and 0.5ml aqueous solution of 2N KOH. The resultant mixture was stirred and refluxed overnight. After cooling to room temperature, organic layer was removed and remaining solution was acidified with 1N HCl to give a precipitate.

Proposed Mechanism

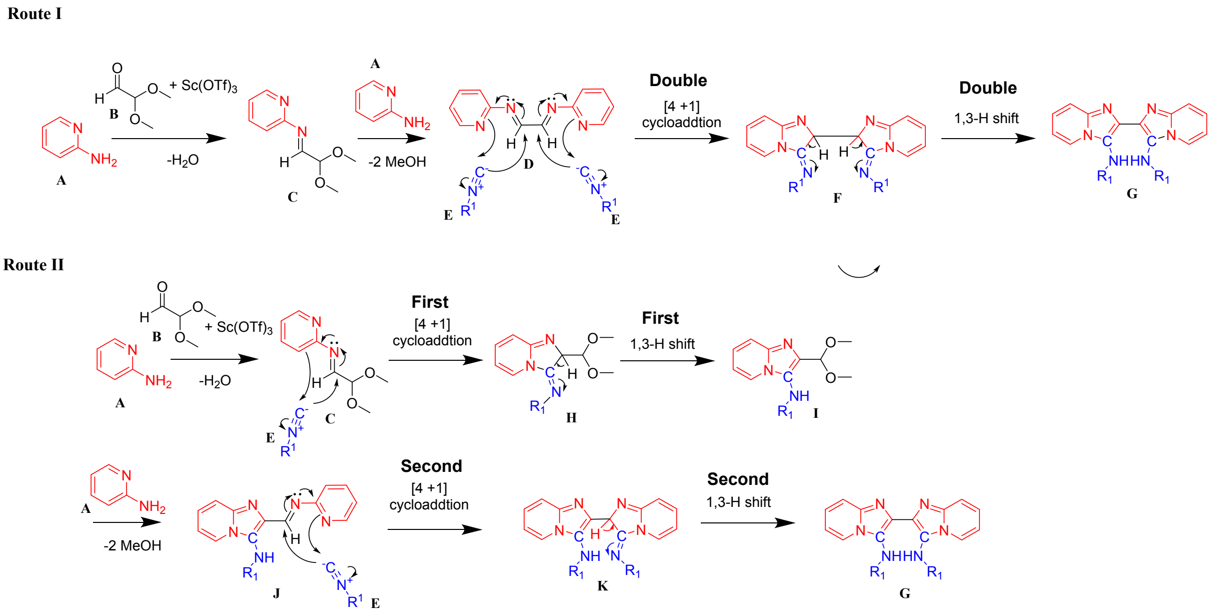

According to the classic GBB reaction mechanism, we proposed two plausible mechanism routes for our imidazole-fused heterocycle dimers reaction.

In route **I**, 2-aminopyridine (**A**) reacts with glyoxal dimethyl acetal (**B**) in the presence of $Sc(OTf)_3$ to form mono-imine intermediate **C**, then **C** undergo the SN_2 reaction with **A** to obtain di-imine intermediate **D**. Then **D** reacts with 2 equiv. isocyanide (**E**) though [4 +1] cycloaddition to give **F**, finally, then aromatization of **F** via double 1,3-H shift to form the imidazole-fused heterocycle dimer **G**.

In route **II**, mono-imine intermediate **C** may preferentially react with 1 equiv. isocyanide (**E**) by first [4 +1] cycloaddition reaction to achieve intermediate **H**, then **H** undergo first 1,3-H shift to form imidazole-fused heterocycle monomer **I**. Then **I** reacts with second equiv. **A** to give **J**, which goes through second [4 +1] cycloaddition with another 1 equiv. **E** to obtain K, followed by second 1,3-H shift to achieve final product **G**.

N3,N3'-di-tert-butyl-[2,2'-biimidazo[1,2-a]pyridine]-3,3'-diamine(3aa)

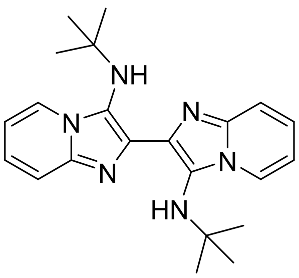

Follow general procedure I on 1 mmol scale. Yellow solid (96 mg, 51%), m.p.: 239.3-239.7°C; TCL (Dichloromethane : Methanol = 97 : 3): R_f = 0.51; ^{1}H NMR (500 MHz, $CDCl_3$) δ 8.29 (dd, *J* = 6.9, 1.3 Hz, 2H), 7.48 (dd, *J* = 8.8, 1.2Hz, 2H), 7.06 (ddd, *J* = 8.9, 6.9, 1.2 Hz, 2H), 6.71 (ddd, *J* = 8.9, 8.8, 1.3 Hz, 2H), 4.91 (brs, 2H), 1.16 (s, 18H).^{13}C NMR (126 MHz, $CDCl_3$) δ 141.6, 133.3, 126.7, 123.7, 123.1, 116.9, 110.8, 56.8, 30.0. HRMS (ESI) m/z: $[M + H]^+$ calcd for $C_{22}H_{29}N_6$, 377.2448; found, 377.2447.

N^3,*N*$^{3'}$-di-tert-butyl-6,6'-dichloro-[2,2'-biimidazo[1,2-*a*]pyridine]-3,3'-diamine(**3ab**)

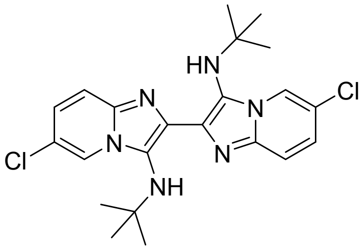

Follow general procedure I on 1 mmol scale. Yellow solid (106 mg, 48%), m.p.: 231.3-233.2°C; TCL (Dichloromethane: Methanol = 97 : 3): R_f = 0.28; ^{1}H NMR (500 MHz, $CDCl_3$) δ 8.30 (d, *J* = 2.1 Hz, 2H), 7.41 (d, *J* = 8.9 Hz, 2H), 7.06 (dd, *J* = 8.9, 2.1 Hz, 2H), 4.79 (s, 2H), 1.14 (s, 18H). ^{13}C NMR (126 MHz, $CDCl_3$) δ 140.1, 133.9, 127.5, 125.0, 121.7, 119.8, 117.4, 57.2, 30.1. HRMS (ESI) m/z: $[M + H]^+$ calcd for $C_{22}H_{27}N_6Cl_2$, 445.1669; found, 445.1668.

6,6'-dibromo-*N*^{3},*N*$^{3'}$-di-tert-butyl-[2,2'-biimidazo[1,2-*a*]pyridine]-3,3'-diamine(**3ac**)

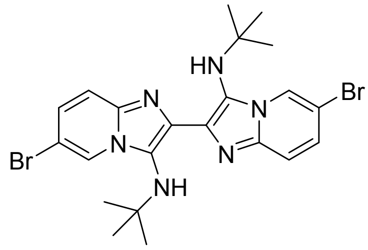

Follow general procedure I on 1 mmol scale. Yellow solid (146 mg, 55%), m.p.: 234.2-235.9 °C; TCL (Dichloromethane : Methanol = 97 : 3): R_f = 0.25; ^{1}H NMR (500 MHz, $CDCl_3$) δ 8.40 (d, *J* =2.0 Hz, 2H), 7.37 (d, *J* = 8.6 Hz, 2H), 7.14 (dd, *J* = 8.6, 2.0 Hz, 2H), 4.79 (s, 2H), 1.14 (s, 18H). ^{13}C NMR (126 MHz, $CDCl_3$) δ 140.2, 134.0, 127.3, 126.8, 123.9, 117.7, 106.2, 57.2, 30.1. HRMS (ESI) m/z: $[M + H]^+$ calcd for $C_{21}H_{31}O_4N_2Br_2$, 533.0645; found, 533.0650.

3,3'-bis(tert-butylamino)-[2,2'-biimidazo[1,2-*a*]pyridine]-7,7'-dicarbonitrile(**3ad**)

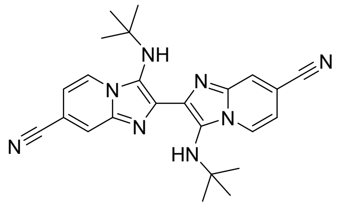

Follow general procedure I on 1 mmol scale. Yellow solid (128 mg, 60%), m.p.: 249.3-251.0°C; TCL (Dichloromethane : Methanol = 97 : 3): R_f = 0.51;^{1}H NMR (500 MHz, DMSO-d_6) δ 8.57 (d, *J* = 7.1 Hz, 2H), 8.26 (d, *J* = 1.3 Hz, 2H), 7.19 (dd, *J* = 7.1, 1.3 Hz, 2H), 5.36 (s, 2H), 0.99 (s, 18H).^{13}C NMR (126 MHz, DMSO-d_6) δ 139.9, 136.6, 129.0, 125.4, 123.8, 118.7, 112.0, 105.5, 57.1, 29.9. HRMS (ESI) m/z: $[M + H]^+$ calcd for $C_{24}H_{27}N_8$, 427.2353; found, 427.2348.

N^3,*N*$^{3'}$-dicyclohexyl-[2,2'-biimidazo[1,2-*a*]pyridine]-3,3'-diamine(**3ae**)

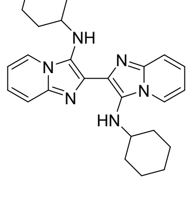

Follow general procedure I on 1 mmol scale. Yellow solid (173 mg, 81%), m.p.: 248.2-248.8°C; TCL (Dichloromethane : Methanol = 97 : 3): R_f = 0.51; ^{1}H NMR (500 MHz, $CDCl_3$) δ 8.05 (dd, *J* = 6.9, 1.2 Hz, 2H), 7.50 (dd, *J* = 8.9, 1.3 Hz, 2H), 7.10 (ddd, *J* = 8.9, 6.8, 1.2 Hz, 2H), 6.77 (ddd, *J* = 6.9, 6.8, 1.3 Hz, 2H), 5.41 (brs, 2H), 3.08 – 3.04 (m, 2H), 1.97 – 1.84 (m, 4H), 1.79 – 1.69 (m, 4H), 1.58 – 1.56 (m, 2H), 1.42 – 1.34 (m, 4H), 1.25 – 1.19 (m, 6H).^{13}C

NMR (126 MHz, $CDCl_3$) δ 140.8, 129.6, 128.1, 122.5, 122.2, 117.1, 111.0, 56.0, 33.9, 25.8, 25.1. HRMS (ESI) m/z: $[M + H]^+$ calcd for $C_{26}H_{33}N_6$, 429.2761; found, 429.2760.

6,6'-dichloro-N^3,$N^{3'}$-dicyclohexyl-[2,2'-biimidazo[1,2-*a*]pyridine]-3,3'-diamine(**3af**)

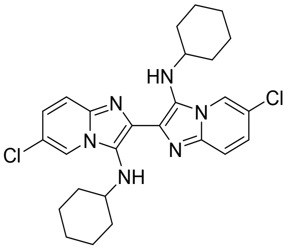

Follow general procedure I on 1 mmol scale. Yellow solid (163 mg, 66%), m.p.: 240.2-242.5 °C; TCL (Dichloromethane : Methanol = 97 : 3): R_f = 0.14; ^1H NMR (500 MHz, $CDCl_3$) δ 8.03 (d, J = 2.2 Hz, 2H), 7.41 (d, J = 8.8 Hz, 2H), 7.01 (dd, J = 8.8, 2.2 Hz, 2H), 5.32 (s, 2H), 3.04 – 3.00 (m, 2H), 1.89 – 1.84 (m, 4H), 1.75 – 1.70 (m, 4H), 1.59 – 1.54 (m, 2H), 1.39 – 1.28 (m, 4H), 1.26 – 1.16 (m, 6H). ^{13}C NMR (126 MHz, $CDCl_3$) δ 139.3, 130.5, 128.8, 123.7, 120.4, 119.8, 117.6, 56.1, 34.0, 25.9, 25.1. HRMS (ESI) m/z: $[M + H]^+$ calcd for $C_{26}H_{31}N_6Cl_2$, 497.1982; found, 497.1982.

6,6'-dibromo-N^3,$N^{3'}$-dicyclohexyl-[2,2'-biimidazo[1,2-*a*]pyridine]-3,3'-diamine(**3ag**)

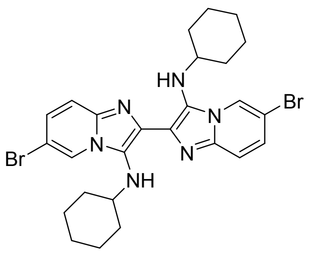

Follow general procedure I on 1 mmol scale. Yellow solid (160 mg, 55%), m.p.: 242.3-243.4 °C; TCL (Dichloromethane : Methanol = 97 : 3): R_f = 0.18; ^1H NMR (500 MHz, $CDCl_3$) δ 8.12 (d, J = 2.7 Hz, 2H), 7.36 (d, J = 9.0 Hz, 2H), 7.11 (dd, J = 9.0, 2.7 Hz, 2H), 5.29 (s, 2H), 3.05 – 2.99 (m, 2H), 1.88 – 1.85 (m, 4H), 1.75 – 1.68 (m, 4H), 1.58 – 1.54 (m, 2H), 1.37 – 1.29 (m, 4H), 1.25 – 1.15 (m, 6H). ^{13}C NMR (126 MHz, $CDCl_3$) δ 139.4, 130.2, 128.7, 125.8, 122.6, 117.9, 106.3, 56.1, 33.9, 25.9, 25.1. HRMS (ESI) m/z: $[M + H]^+$ calcd for $C_{26}H_{31}N_6Br_2$, 585.0971; found, 585.0969.

7,7'-dibromo-N^3,$N^{3'}$-dicyclohexyl-[2,2'-biimidazo[1,2-*a*]pyridine]-3,3'-diamine(**3ah**)

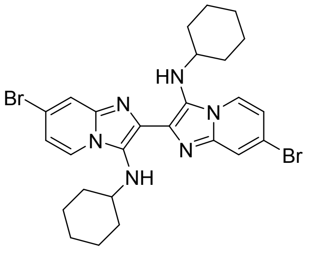

Follow general procedure I on 1 mmol scale. Yellow solid (120 mg, 41%), m.p.: 272.0-274.2 °C; TCL (Dichloromethane : Methanol = 97 : 3): R_f = 0.22; ^1H NMR (500 MHz, $CDCl_3$) δ 7.87 (d, J = 7.3 Hz, 2H), 7.66 (d, J = 2.0 Hz, 2H), 6.84 (dd, J = 2.0, 7.3 Hz, 2H), 5.31 (s, 2H), 3.05 – 2.94 (m, 2H), 1.90 – 1.83 (m, 4H), 1.74 – 1.71 (m, 5H), 1.57 – 1.55 (m, 2H), 1.37 – 1.28 (m, 4H), 1.26 – 1.14 (m, 6H). ^{13}C NMR (126 MHz, $CDCl_3$) δ 140.9, 128.8, 122.9, 119.3, 115.3, 56.3, 34.0, 25.9, 25.1. HRMS (ESI) m/z: $[M + H]^+$ calcd for $C_{26}H_{30}N_6Br_2$, 585.0971; found, 585.0975.

N^3,$N^{3'}$-dicyclohexyl-[2,2'-biimidazo[1,2-*a*]pyrazine]-3,3'-diamine(**3ai**)

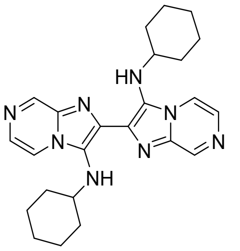

Follow general procedure I on 1 mmol scale. Yellow solid (84 mg, 39%), m.p.: 216.7-217.9 °C; TCL (Dichloromethane : Methanol = 97 : 3): R_f = 0.24; ^1H NMR (500 MHz, $CDCl_3$) δ 8.89 (d, J = 1.6 Hz, 2H), 7.89 (dd, J = 4.7, 1.6 Hz, 2H), 7.79 (d, J = 4.7, 2H), 5.72 (s, 2H), 3.23 – 3.12 (m, 2H), 1.91 – 1.86 (m, 4H), 1.79 – 1.72 (m, 4H), 1.60 – 1.55 (m, 2H), 1.42 –– 1.35 (m, 4H), 1.29 – 1.21 (m, 6H). ^{13}C NMR (126 MHz, $CDCl_3$) δ 143.1, 136.2, 130.6, 130.5, 128.9, 115.6, 55.6, 34.2, 25.8, 24.9. HRMS (ESI) m/z: $[M + H]^+$ calcd for $C_{24}H_{31}N_8$, 431.2666; found, 431.2663.

N^3,$N^{3'}$-dicyclohexyl-6,6'-diiodo-[2,2'-biimidazo[1,2-*a*]pyridine]-3,3'-diamine(**3aj**)

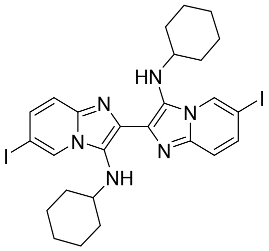

Follow general procedure I on 1 mmol scale. Yellow solid (173 mg, 51%), m.p.: 234.3-235.2 °C; TCL (Dichloromethane : Methanol = 97 : 3): R_f = 0.22; ^{1}H NMR (500 MHz, $CDCl_3$) δ 8.23 (d, *J* = 1.7 Hz, 2H), 7.276 (d, *J* = 6.1 Hz, 2H), 7.21 (dd, *J* = 6.1, 1.7 Hz, 2H), 5.27 (s, 2H), 3.06 – 2.93 (m, 2H), 1.89 – 1.82 (m, 4H), 1.75 – 1.66 (m, 4H), 1.59 – 1.53 (m, 2H), 1.37 – 1.26 (m, 4H), 1.26 – 1.12 (m, 6H). ^{13}C NMR (126 MHz, $CDCl_3$) δ 139.5, 130.3, 129.8, 128.3, 127.6, 118.3, 74.4, 56.2, 33.9, 25.9, 25.04. HRMS (ESI) m/z: $[M + H]^+$ calcd for $C_{26}H_{31}N_6I_2$, 681.0694; found, 681.0697.

N^3,$N^{3'}$-dicyclohexyl-6,6'-bis(trifluoromethyl)-[2,2'-biimidazo[1,2-*a*]pyridine]-3,3'-diamine(**3ak**)

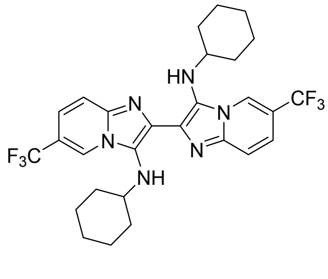

Follow general procedure I on 1 mmol scale. Yellow solid (123 mg, 44%), m.p.: 235.5-236.6 °C; TCL (Dichloromethane : Methanol = 97 : 3): R_f= 0.60; ^{1}H NMR (500 MHz, $CDCl_3$) δ 8.37 (d, *J* = 1.4 Hz, 2H), 7.56 (d, *J* = 8.9 Hz, 2H), 7.21 (dd, *J* = 8.9, 1.4 Hz, 2H), 5.33 (s, 2H), 3.07 – 3.01 (m, 2H), 1.90 – 1.87 (m, 4H), 1.77 – 1.71 (m, 4H), 1.59 – 1.55 (m, 2H), 1.43 – 1.31 (m, 4H), 1.29 – 1.14 (m, 6H). ^{13}C NMR (126 MHz, $CDCl_3$) δ 140.59, 130.70, 129.71, 125.12 (q, *J* = 269.9 Hz), 121.74 (dd, *J* = 11.4, 5.6 Hz), 118.63, 117.84, 116.00 (q, *J* = 34.0 Hz), *56.58, 34.00, 25.86, 25.03.* , 56.6, 34.0, 25.8, 25.0. HRMS (ESI) m/z: $[M + H]^+$ calcd for $C_{28}H_{31}N_6F_6$, 565.2509; found, 565.2506.

N^3,$N^{3'}$-dicyclohexyl-8,8'-dimethyl-[2,2'-biimidazo[1,2-*a*]pyridine]-3,3'-diamine(**3al**)

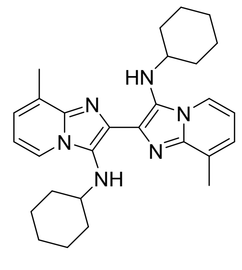

Follow general procedure I on 1 mmol scale. Yellow solid (80 mg, 35%), m.p.: 231.5-233.2 °C; TCL (Dichloromethane : Methanol = 97 : 3): R_f = 0.18; ^{1}H NMR (500 MHz, $CDCl_3$) δ 7.93 (dd, *J* = 7.0, 2.4 Hz, 2H), 6.87 (dd, *J* = 7.0, 6.9 Hz, 2H), 6.67 (dd, *J* = 6.9, 2.4 Hz, 2H), 5.53 (s, 2H), 3.15 – 3.10 (m, 2H), 2.60 (s, 6H), 1.95 – 1.87 (m, 4H), 1.79 – 1.71 (m, 4H), 1.57 – 1.53 (m, 2H), 1.45 – 1.35 (m, 4H), 1.26 – 1.17 (m, 6H).^{13}C NMR (126 MHz, $CDCl_3$) δ 140.3, 128.8, 126.3, 121.1, 120.5, 112.7, 111.4, 56.6, 42.1, 33.9, 27.1, 25.9, 24.9, 17.1. HRMS (ESI) m/z: $[M + H]^+$ calcd for $C_{28}H_{37}N_6$, 457.3074; found, 457.3074.

Dimethyl 3,3'-bis(cyclohexylamino)-[2,2'-biimidazo[1,2-*a*]pyridine]-6,6'-dicarboxylate(**3am**)

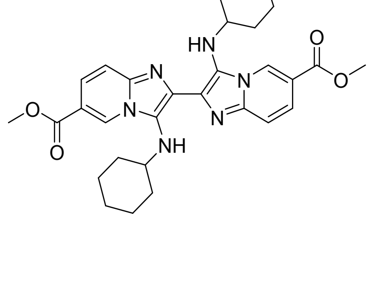

Follow general procedure I on 1 mmol scale. Yellow solid (124 mg, 46%), m.p.: 258.2-260.5°C; TCL (Dichloromethane : Methanol = 97 : 3): R_f = 0.33; ^{1}H NMR (500 MHz, $CDCl_3$) δ 8.79 (d, *J* = 1.7 Hz, 2H), 7.62 (dd, *J* = 9.5, 1.7 Hz, 2H), 7.46 (d, *J* = 9.5 Hz, 2H), 5.35 (s, 2H), 3.97 (s, 6H), 3.13 – 3.05 (m, 2H), 1.92 – 1.87 (m, 4H), 1.75 – 1.72 (m, 4H), 1.58– 1.54 (m, 2H), 1.42 – 1.33 (m, 4H), 1.27 – 1.18 (m, 6H). ^{13}C NMR (126 MHz,

CDCl$_3$) δ 166.0, 141.4, 130.5, 129.4, 127.1, 122.4, 116.5, 115.5, 77.4, 77.2, 76.9, 56.5, 52.5, 33.9, 25.9, 25.0. HRMS (ESI) m/z: $[M + H]^+$ calcd for $C_{30}H_{37}O_4N_6$, 545.2871; found, 545.2867.

N^3,*N*$^{3'}$-bis(2,4,4-trimethylpentan-2-yl)-[2,2'-biimidazo[1,2-*a*]pyridine]-3,3'-diamine(**3an**)

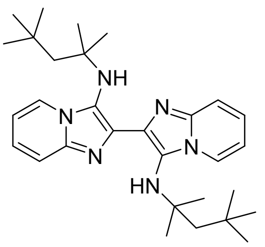

Follow general procedure I on 1 mmol scale. Yellow solid (120 mg, 49%), m.p.: 241.3-241.3°C; TCL (Dichloromethane : Methanol = 97 : 3): R_f = 0.51; ^{1}H NMR (500 MHz, CDCl$_3$) δ 8.31 (dd, *J* = 6.9, 1.2 Hz, 2H), 7.47 (dd, *J* = 9.1, 1.2 Hz, 2H), 7.16 – 6.99 (m, 2H), 6.79 – 6.65 (m, 2H), 4.96 (s, 2H), 1.75 (s, 4H), 1.14 (s, 18H), 1.11 (s, 12H). ^{13}C NMR (126 MHz, CDCl$_3$) δ 141.6, 133.8, 126.4, 123.8, 122.9, 116.8, 110.7, 61.1, 56.6, 32.0, 31.8, 28.7. HRMS (ESI) m/z: $[M + H]^+$ calcd for $C_{30}H_{45}N_6$, 489.3700; found, 489.3692.

dimethyl 3,3'-([2,2'-biimidazo[1,2-*a*]pyridine]-3,3'-diylbis(azanediyl))dipropionate(**3ao**)

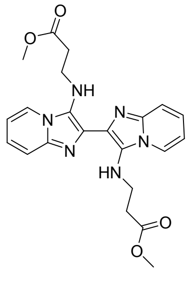

Follow general procedure I on 1 mmol scale. Yellow solid (99 mg, 46%), m.p.: 249.1-249.8°C; TCL (Dichloromethane : Methanol = 97 : 3): R_f = 0.51; ^{1}H NMR (500 MHz, CDCl$_3$) δ 8.19 – 8.12 (m, 2H), 7.48 (dd, *J* = 9.1, 1.4 Hz, 2H), 7.10 (ddd, *J* = 9.1, 6.6, 1.3 Hz, 2H), 6.80 (ddd, *J* = 6.8, 6.6, 1.4 Hz, 2H), 5.52 (s, 2H), 3.67 (s, 6H), 3.45 – 3.42 (m, 4H), 2.65 (t, *J* = 5.9 Hz, 4H). ^{13}C NMR (126 MHz, CDCl$_3$) δ 172.8, 141.2, 129.2, 127.9, 122.9, 122.5, 117.1, 111.5, 51.6, 42.9, 34.8. HRMS (ESI) m/z: $[M - H]^-$ calcd for $C_{22}H_{23}N_6O_4$, 435.1762; found, 435.1767.

N^3,*N*$^{3'}$-dibenzyl-6,6'-dibromo-[2,2'-biimidazo[1,2-*a*]pyridine]-3,3'-diamine(**3ap**)

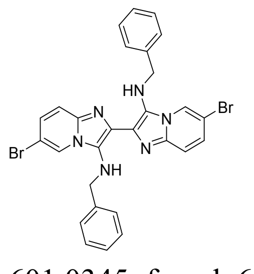

Follow general procedure I on 1 mmol scale. Yellow solid (134 mg, 45%), m.p.: 204.0-206.5 °C; TCL (Dichloromethane : Methanol = 97 : 3): R_f = 0.71; ^{1}H NMR (500 MHz, CDCl$_3$) δ 8.07 (d, *J* = 2.2 Hz, 2H), 7.45 – 7.40 (m, 4H), 7.33 (dd, *J* = 8.4, 2.2 Hz, 6H), 7.30 – 7.26 (m, 2H), 7.12 (dd, *J* = 9.5, 1.9 Hz, 2H), 5.58 (t, *J* = 6.9 Hz, 2H), 4.24 (d, *J* = 6.6 Hz, 4H). ^{13}C NMR (126 MHz, CDCl$_3$) δ 139.6, 139.2, 129.4, 128.6, 128.5, 127.6, 126.7, 122.6, 117.7, 106.6, 52.3. HRMS (ESI) m/z: $[M + H]^+$ calcd for $C_{28}H_{23}N_6Br_2$, 601.0345; found, 601.0345.

N^3,*N*$^{3'}$-dibenzyl-6,6'-diiodo-[2,2'-biimidazo[1,2-*a*]pyridine]-3,3'-diamine(**3aq**)

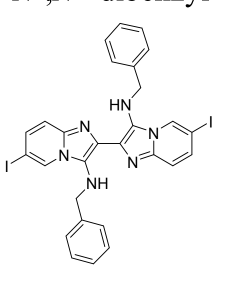

Follow general procedure I on 1 mmol scale. Yellow solid (251 mg, 72%), m.p.: 226.5-228.3 °C; TCL (Dichloromethane : Methanol = 97 : 3): R_f = 0.36; ^{1}H NMR (500 MHz, CDCl$_3$) δ 8.17 (d, *J* = 1.3 Hz, 2H), 7.45 – 7.39 (m, 4H), 7.26 – 7.23 (m, 10H), 5.59 (s, 2H), 4.23 (d, *J* = 6.4 Hz, 4H). ^{13}C NMR (126 MHz, CDCl$_3$) δ 139.8, 139.8, 139.2, 131.2, 129.1, 128.6, 128.5, 127.6, 121.2, 118.2, 77.41, 77.2, 76.9, 74.7, 52.5. HRMS (ESI) m/z: $[M + H]^+$ calcd for $C_{28}H_{23}N_6I_2$, 697.0068; found, 697.0072.

3,3'-bis(benzylamino)-[2,2'-biimidazo[1,2-*a*]pyridine]-7,7'-dicarbonitrile(**3ar**)

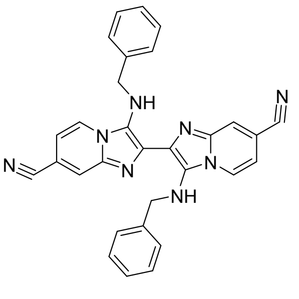

Follow general procedure I on 1 mmol scale. Yellow solid (77 mg, 31%), m.p.: 236.3-236.7°C; TCL (Dichloromethane : Methanol = 97 : 3): R_f = 0.51; ^{1}H NMR (500 MHz, DMSO) δ 8.44 (d, *J* = 7.1 Hz, 2H), 8.21 (d, *J* = 1.3 Hz, 2H), 7.36 – 7.28 (m, 4H), 7.27 – 7.13 (m, 8H), 6.35 (s, 2H), 4.42 (d, *J* = 7.0 Hz, 4H). ^{13}C NMR (126 MHz, DMSO) δ 139.7, 138.1, 132.1, 130.1, 128.7, 128.4, 127.6, 124.1, 124.0, 118.9, 112.7, 103.8, 50.3. HRMS (ESI) m/z: $[M + H]^+$ calcd for $C_{30}H_{23}N_8$, 495.2040; found, 495.2041.

3,3'-bis(benzylamino)-[2,2'-biimidazo[1,2-*a*]pyridine]-6,6'-dicarbonitrile(**3as**)

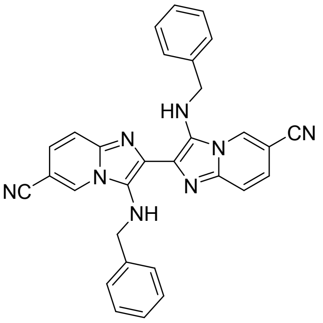

Follow general procedure I on 1 mmol scale. Yellow solid (42 mg, 17%), m.p.: 245.8-246.0°C; TCL (Dichloromethane : Methanol = 97 : 3): R_f = 0.51; ^{1}H NMR (500 MHz, DMSO) δ 8.98 (d, *J* = 1.3 Hz, 2H), 7.62 (dd, *J* = 9.3, 1.3 Hz, 2H), 7.40 – 7.33 (m, 6H), 7.29 – 7.19 (m, 6H), 5.98 (s, 2H), 4.36 (d, *J* = 5.4 Hz, 4H). ^{13}C NMR (126 MHz, DMSO) δ 139.8, 139.6, 130.7, 130.6, 129.2, 128.8, 128.6, 127.6, 124.6, 123.6, 117.8, 97.2, 51.2. HRMS (ESI) m/z: $[M + H]^+$ calcd for $C_{30}H_{23}N_8$, 495.2040; found, 495.2038.

4,4'-(([2,2'-biimidazo[1,2-*a*]pyridine]-3,3'-diylbis(azanediyl))bis(methylene))dibenzonitrile(**3at**)

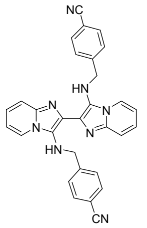

Follow general procedure I on 1 mmol scale. Yellow solid (80 mg, 32%), m.p.: 246.1-241.8°C; TCL (Dichloromethane : Methanol = 97 : 3): R_f = 0.51; ^{1}H NMR (500 MHz, $CDCl_3$) δ 8.01 (dd, *J* = 6.8, 1.3 Hz, 2H), 7.65– 7.58 (m, 8H), 7.47 (dd, *J* = 9.1, 1.3 Hz, 2H), 7.14 (ddd, *J* = 9.0, 6.7, 1.3 Hz, 2H), 6.80 (dd, *J* = 6.8, 1.1 Hz, 2H), 5.69 (t, *J* = 7.3 Hz, 2H), 4.35 (d, *J* = 7.3 Hz, 4H). ^{13}C NMR (126 MHz, $CDCl_3$) δ 144.9, 141.2, 132.1, 128.9, 128.1, 123.5, 122.1, 118.9, 117.1, 111.9, 111.1, 98.1, 51.6. HRMS (ESI) m/z: $[M + H]^+$ calcd for $C_{30}H_{23}N_8$, 495.2040; found, 495.2039.

N^3,$N^{3'}$-diphenethyl-[2,2'-biimidazo[1,2-*a*]pyridine]-3,3'-diamine(**3au**)

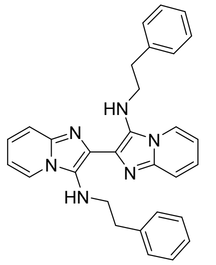

Follow general procedure I on 1 mmol scale. Yellow solid (175 mg, 74%), m.p.: 246.2-246.8°C; TCL (Dichloromethane : Methanol = 97 : 3): R_f = 0.51; ^{1}H NMR (500 MHz, $CDCl_3$) δ 7.86 (dd, *J* = 6.9, 1.4 Hz, 2H), 7.47 (dd, *J* = 9.0, 1.2 Hz, 2H), 7.36 – 7.26 (m, 10H), 7.10 (ddd, *J* = 9.0, 6.9, 1.4 Hz, 2H), 6.73 (dd, *J* = 6.8, 1.2 Hz, 2H), 5.46(s, 2H), 3.41 (t, *J* = 7.2 Hz, 4H), 3.01 (t, *J* = 7.2 Hz, 4H). ^{13}C NMR (126 MHz, CDCl3) δ 141.1, 139.8, 129.0, 128.8, 128.7, 128.4, 126.2, 122.7, 122.3, 117.1, 111.3, 49.1, 36.9.HRMS (ESI) m/z: $[M + H]^+$ calcd for $C_{30}H_{29}N_6$, 473.2448; found, 473.2444.

6,6'-dichloro-N^3,$N^{3'}$-diphenethyl-[2,2'-biimidazo[1,2-*a*]pyridine]-3,3'-diamine(**3av**)

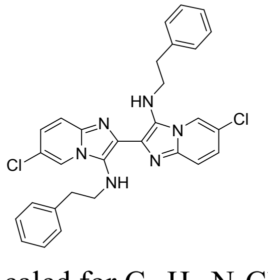

Follow general procedure I on 1 mmol scale. Yellow solid (166 mg, 61%), m.p.: 202.3-203.6 °C; TCL (Dichloromethane : Methanol = 97 : 3): R_f = 0.40; ^{1}H NMR (500 MHz, $CDCl_3$) *δ* 7.71 (d, *J* = 2.1 Hz, 2H), 7.42 (d, *J* = 9.5 Hz, 2H), 7.31 – 7.27 (m, 4H), 7.26 – 7.20 (m, 6H), 7.08 (dd, *J* = 9.5, 1.9 Hz, 2H), 5.34 (s, 2H), 3.34 (t, *J* = 6.9 Hz, 4H), 2.96 (t, *J* = 6.9 Hz, 4H). ^{13}C NMR (126 MHz, $CDCl_3$) *δ* 139.5, 138.1, 129.6, 129.2, 128.5, 127.7, 126.6, 124.5, 122.2, 120.9, 116.4, 49.4, 36.9. HRMS (ESI) m/z: $[M + H]^+$ calcd for $C_{30}H_{27}N_6Cl_2$, 541.1669; found, 541.1669.

6,6'-dibromo-N^3,$N^{3'}$-diphenethyl-[2,2'-biimidazo[1,2-*a*]pyridine]-3,3'-diamine(**3aw**)

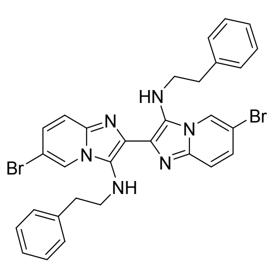

Follow general procedure I on 1 mmol scale. Yellow solid (157 mg, 50%), m.p.: 207.2-208.6 °C; TCL (Dichloromethane : Methanol = 97 : 3): R_f = 0.30; ^1H NMR (500 MHz, $CDCl_3$) δ 7.85 (d, J = 2.7 Hz, 2H), 7.32 – 7.22 (m, 12H), 7.09 (dd, J = 9.5, 1.9 Hz, 2H), 5.34 (t, J = 7.3 Hz, 2H), 3.35 – 3.31 (m, 4H), 2.95 (t, J = 7.0 Hz, 4H). ^{13}C NMR (126 MHz, $CDCl_3$) δ 139.6, 139.5, 129.4, 129.1, 128.6, 126.5, 126.1, 122.4, 117.8, 106.5, 49.0, 37.0. HRMS (ESI) m/z: $[M + H]^+$ calcd for $C_{30}H_{27}N_6Br_2$, 629.0658; found, 629.0660.

6,6'-diiodo-N^3,$N^{3'}$-diphenethyl-[2,2'-biimidazo[1,2-*a*]pyridine]-3,3'-diamine(**3ax**)

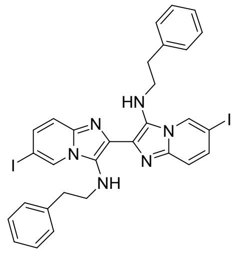

Follow general procedure I on 1 mmol scale. Yellow solid (252 mg, 70%), m.p.: 212.4-213.8 °C; TCL (Dichloromethane : Methanol = 97 : 3): R_f = 0.31; ^1H NMR (500 MHz, $CDCl_3$) δ 7.99 – 7.95 (m, 2H), 7.33 – 7.28 (m, 4H), 7.26 – 7.11 (m, 10H), 5.32 (t, J = 7.4 Hz, 2H), 3.34 – 3.29 (m, 4H), 2.94 (t, J = 7.0 Hz, 4H). ^{13}C NMR (126 MHz, $CDCl_3$) δ 139.6, 139.6, 130.6, 129.1, 128.9, 128.9, 128.6, 127.3, 126.5, 118.3, 74.5, 49.1, 37.0. HRMS (ESI) m/z: $[M + H]^+$ calcd for $C_{30}H_{27}N_6I_2$, 725.0381; found, 725.0384.

N^3,$N^{3'}$-diphenethyl-[2,2'-biimidazo[1,2-*a*]pyrazine]-3,3'-diamine(**3ay**)

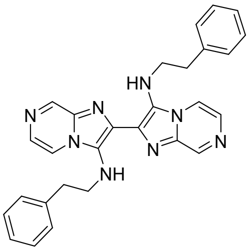

Follow general procedure II on 1 mmol scale. Yellow solid (103 mg, 43%), m.p.: 212.5-212.9 °C; TCL (Dichloromethane : Methanol = 97 : 3): R_f = 0.13; ^1H NMR (500 MHz, $CDCl_3$) δ 8.78 (d, J = 1.4 Hz, 2H), 7.74 (d, J = 4.6 Hz, 2H), 7.68 (dd, J = 4.6, 1.4 Hz, 2H), 7.32 – 7.26 (m, 5H), 7.25 – 7.22 (m, 5H), 5.79 (t, J = 7.3 Hz, 2H), 3.49 (t, J = 6.9 Hz, 4H), 2.97 (t, J = 6.9 Hz, 4H). ^{13}C NMR (126 MHz, $CDCl_3$) δ 143.1, 139.1, 136.2, 131.3, 129.9, 129.1, 128.8, 128.6, 126.7, 115.3, 48.3, 37.2. HRMS (ESI) m/z: $[M + H]^+$ calcd for $C_{28}H_{27}N_8$, 475.2353; found, 475.2353.

N^3,$N^{3'}$-bis(2-ethylphenyl)-8,8'-dimethyl-[2,2'-biimidazo[1,2-*a*]pyridine]-3,3'-diamine(**3ba**)

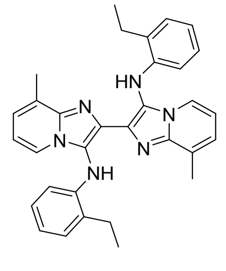

Follow general procedure I on 1 mmol scale. Yellow solid (93 mg, 37%), m.p.: 263.5-264.8 °C; TCL (Dichloromethane : Methanol = 97 : 3): R_f = 0.51; ^1H NMR (500 MHz, $CDCl_3$) δ 7.93 (s, 2H),7.50 (dd, J = 6.8, 1.4 Hz, 2H), 7.23 (dd, J = 7.5, 1.8 Hz, 2H) 6.95 – 6.92 (m, 4H), 6.87 – 6.84 (m, 2H) 6.63 (dd, J = 6.8, 2.0 Hz, 2H), 6.03 (dd, J = 8.0, 1.3 Hz, 2H), 3.12 (q, J = 7.6 Hz, 4H), 2.60 (s, 6H), 1.53 (t, J = 7.6 Hz, 6H), 1.79 – 1.71 (m, 4H), 1.55 (p, J = 4.5, 5.0 Hz, 2H), 1.45 – 1.35 (m, 4H), 1.26 – 1.17 (m, 6H). ^{13}C NMR (126 MHz, $CDCl_3$) δ 141.8, 131.3, 128.8, 126.9, 126.9, 123.4, 121.5, 120.5, 114.4, 112.1, 24.6, 16.9, 13.9. HRMS (ESI) m/z: $[M + H]^+$ calcd for $C_{32}H_{33}N_6$, 501.2761; found, 501.2762.

6,6'-dichloro-N^3,$N^{3'}$-bis(2-ethylphenyl)-[2,2'-biimidazo[1,2-*a*]pyridine]-3,3'-diamine(**3bb**)

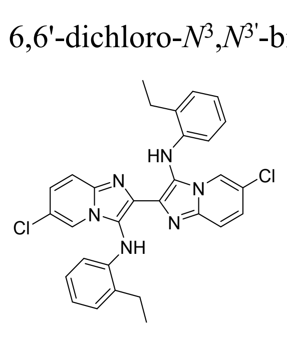

Follow general procedure I on 1 mmol scale. Yellow solid (144 mg, 53%), m.p.: 252.1-252.8 °C; TCL (Dichloromethane : Methanol = 97 :

3): R_f = 0.37; ^{1}H NMR (500 MHz, $CDCl_3$) δ 7.61 (d, J = 2.0 Hz, 2H), 7.52 (s, 2H), 7.43 (dd, J = 9.3, 1.2 Hz, 2H), 7.27 – 7.25 (m, 2H), 7.09 (dd, J = 9.4, 2.0 Hz, 2H), 6.99 – 6.96 (m, 2H), 6.92 – 6.89 (m, 2H), 6.00 (dd, J = 7.9, 1.4 Hz, 2H), 2.93 (q, J = 7.6 Hz, 4H), 1.55 (t, J = 7.6 Hz, 6H). ^{13}C NMR (126 MHz, $CDCl_3$) δ 141.1, 140.3, 131.6, 130.8, 129.2, 127.2, 124.9, 123.8, 121.2, 120.3, 117.8, 114.4, 24.6, 14.0. HRMS (ESI) m/z: $[M + H]^+$ calcd for $C_{30}H_{27}N_6Cl_2$, 541.1669; found, 541.1667.

6,6'-dibromo-*N*3,*N*3'-bis(2-ethylphenyl)-[2,2'-biimidazo[1,2-*a*]pyridine]-3,3'-diamine(**3bc**)

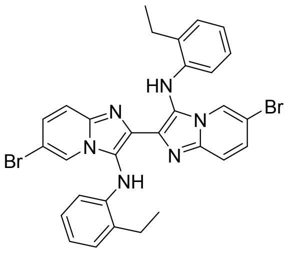

Follow general procedure I on 1 mmol scale. Yellow solid (203 mg, 65%), m.p.: 279.5-280.9 °C; TCL (Dichloromethane : Methanol = 97 : 3): R_f = 0.33; ^{1}H NMR (500 MHz, $CDCl_3$) δ 7.71 (d, J = 1.6 Hz, 2H), 7.51 (s, 2H), 7.38 (dd, J = 9.6, 2.2 Hz, 2H), 7.29 – 7.23 (m, 2H), 7.19 – 7.17 (m, 2H), 6.99 – 6.96 (m, 2H), 6.92 – 6.89 (m, 2H), 5.99 (dd, J = 7.7, 1.4 Hz, 2H), 2.73 (q, J = 7.6 Hz, 4H), 1.35 (t, J = 7.6 Hz, 6H). ^{13}C NMR (126 MHz, $CDCl_3$) δ 141.1, 140.4, 131.6, 130.6, 129.2, 127.2, 126.9, 123.7, 123.4, 121.2, 118.1, 114.3, 106.8, 24.6, 14.0. HRMS (ESI) m/z: $[M + H]^+$ calcd for $C_{30}H_{27}N_6Br_2$, 629.0658; found, 629.0658.

7,7'-dibromo-*N*3,*N*3'-bis(2-ethylphenyl)-[2,2'-biimidazo[1,2-*a*]pyridine]-3,3'-diamine(**3bd**)

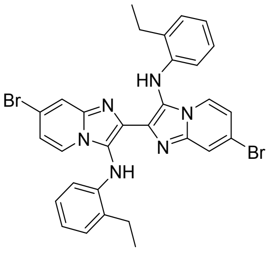

Follow general procedure I on 1 mmol scale. Yellow solid (172 mg, 55%), m.p.: 308.2-309.7 °C; TCL (Dichloromethane : Methanol = 97 : 3): R_f = 0.39; ^{1}H NMR (500 MHz, $CDCl_3$) δ 7.67 (d, J = 1.9 Hz, 2H), 7.53 (s, 2H), 7.43 (d, J = 7.3 Hz, 2H), 7.24 (dd, J = 7.3, 2.0 Hz, 2H), 6.97 – 6.94 (m, 2H), 6.90 – 6.87 (m, 2H), 6.83 – 6.81 (m, 2H), 6.00 (dd, J = 7.3, 1.2 Hz, 2H), 2.91 (q, J = 7.6 Hz, 4H), 1.53 (t, J = 7.6 Hz, 6H). ^{13}C NMR (126 MHz, $CDCl_3$) δ 141.0, 131.8, 129.3, 127.0, 123.8, 123.7, 121.2, 119.6, 115.8, 114.5, 24.6, 14.1. HRMS (ESI) m/z: $[M + H]^+$ calcd for $C_{30}H_{27}N_6Br_2$, 629.0658; found, 629.0659.

*N*3,*N*3'-bis(2-ethylphenyl)-6,6'-diiodo-[2,2'-biimidazo[1,2-*a*]pyridine]-3,3'-diamine(**3be**)

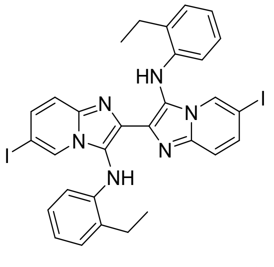

Follow general procedure I on 1 mmol scale. Yellow solid (254mg, 70%), m.p.: 297.1-297.6 °C; TCL (Dichloromethane : Methanol = 97 : 3): R_f = 0.16; ^{1}H NMR (500 MHz, $CDCl_3$) δ 7.83 (d, J = 1.8 Hz, 2H), 7.51 (s, 2H), 7.29 – 7.24 (m, 4H), 7.24 (s, 2H), 6.99 – 6.96 (m, 2H), 6.91 – 6.88 (m, 2H), 5.98 (dd, J = 8.0, 1.8 Hz, 2H), 2.93 (q, J = 7.6 Hz, 4H), 1.54 (t, J = 7.6 Hz, 6H). ^{13}C NMR (126 MHz, $CDCl_3$) δ 141.2, 140.5, 137.9, 131.5, 130.1, 129.2, 128.2, 127.2, 123.3, 121.1, 118.5, 114.3, 74.9, 24.6, 14.0. HRMS (ESI) m/z: $[M + H]^+$ calcd for $C_{30}H_{27}N_6I_2$, 725.0381; found, 725.0380.

*N*3,*N*3'-bis(2-ethylphenyl)-6,6'-bis(trifluoromethyl)-[2,2'-biimidazo[1,2-*a*]pyridine]-3,3'-diamine(**3bf**)

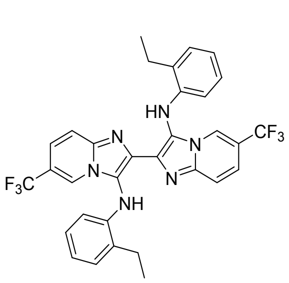

Follow general procedure I on 1 mmol scale. Yellow solid (105 mg, 35%), m.p.: 238.5-240.3 °C; TCL (Dichloromethane : Methanol = 97 :

3): R_f = 0.52; ^{1}H NMR (500 MHz, $CDCl_3$) δ 7.94 (d, J = 1.5 Hz, 2H), 7.61 (s, 2H), 7.59 – 7.57 (m, 2H), 7.31– 7.27 (m, 4H), 7.02 – 6.89 (m, 4H), 6.00 (dd, J = 7.9, 1.4 Hz, 2H), 2.97 (q, J = 7.5 Hz, 4H), 1.57 (t, J = 7.5 Hz, 6H).^{13}C NMR (126 MHz, $CDCl_3$) δ 141.57, 140.94, 131.87, 131.08, 129.41, 127.17, 124.89, 123.54 (q, J = 272.0 Hz), 122.45 (dd, J = 5.5 Hz), 121.59, 119.55 (d, J = 2.5 Hz), 118.10, 116.41 (q, J = 34.2 Hz), 114.33, 24.58, 14.04. HRMS (ESI) m/z: $[M + H]^+$ calcd for $C_{32}H_{27}N_6F_6$, 609.2196; found, 609.2191.

N^3,*N*$^{3'}$-bis(2-ethylphenyl)-[2,2'-biimidazo[1,2-*a*]pyrimidine]-3,3'-diamine(**3bg**)

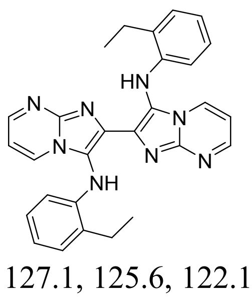

Follow general procedure I on 1 mmol scale. Yellow solid (70 mg, 30%), m.p.: 270.5-271.8 °C; TCL (Dichloromethane : Methanol = 97 : 3): R_f = 0.35; ^{1}H NMR (500 MHz, $CDCl_3$) δ 8.96 (dd, J = 4.7, 1.7 Hz, 2H), 7.88 (s, 2H), 7.79 (dd, J = 4.6, 1.7 Hz, 2H), 7.45 (dd, J = 4.7, 4.6 Hz, 2H), 7.31 (dd, J = 7.6, 1,7 Hz, 2H), 7.03 – 7.00 (m, 2H), 6.99 – 6.94 (m, 2H), 6.06 (dd, J = 7.7, 1.4 Hz, 2H), 2.98 (q, J = 7.5 Hz, 4H), 1.56 (t, J = 7.5 Hz, 6H). ^{13}C NMR (126 MHz, $CDCl_3$) δ 143.3, 139.7, 136.9, 132.8, 130.7, 129.6, 129.1, 127.1, 125.6, 122.1, 116.7, 115.7, 24.8, 14.2. HRMS (ESI) m/z: $[M + H]^+$ calcd for $C_{28}H_{27}N_8$, 475.2353; found, 475.2352.

N^3,*N*$^{3'}$-bis(2-ethylphenyl)-[2,2'-biimidazo[1,2-*a*]pyrazine]-3,3'-diamine(**3bh**)

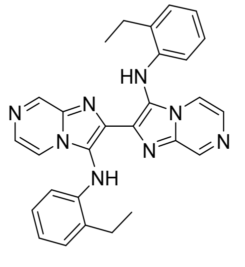

Follow general procedure I on 1 mmol scale. Yellow solid (82 mg, 35%), m.p.: 264.2-266.1 °C; TCL (Dichloromethane : Methanol = 95 : 5): R_f = 0.40; ^{1}H NMR (500 MHz, $CDCl_3$) δ 8.98 – 8.94 (m, 2H), 7.88 (s, 2H), 7.79 (d, J = 4.6 Hz, 2H), 7.47 – 7.42 (m, 2H), 7.33 – 7.28 (m, 2H), 7.05 – 6.93 (m, 4H), 6.06 (dd, J = 7.9, 1.6 Hz, 2H), 2.98 (q, J = 7.6 Hz, 4H), 1.56 (t, J = 7.6 Hz, 6H). ^{13}C NMR (126 MHz, $CDCl_3$) δ 143.2, 139.7, 136.9, 132.8, 130.7, 129.6, 129.1, 127.0, 125.6, 122.1, 116.6, 115.6, 24.8, 14.1. HRMS (ESI) m/z: $[M + H]^+$ calcd for $C_{28}H_{27}N_8$, 475.2353; found, 475.2352.

6,6'-dibromo-*N*^{3},*N*$^{3'}$-bis(2-isopropylphenyl)-[2,2'-biimidazo[1,2-*a*]pyridine]-3,3'-diamine(**3bi**)

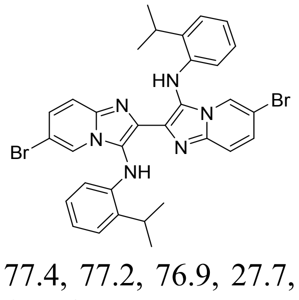

Follow general procedure I on 1 mmol scale. Yellow solid (183 mg, 54%), m.p.: 249.3-251.0°C; TCL (Dichloromethane : Methanol = 97 : 3): R_f = 0.51; ^{1}H NMR (500 MHz, $CDCl_3$) δ 7.71 (d, J = 2.4 Hz, 2H), 7.64 (s, 2H), 7.40 – 7.31 (m, 4H), 7.17 (dd, J = 8.6, 1.9 Hz, 2H), 7.01 – 6.91 (m, 4H), 6.03 – 5.96 (m, 2H), 3.55 – 3.48 (m, 2H), 1.56 (d, J = 6.8 Hz, 12H).^{13}C NMR (126 MHz, $CDCl_3$) δ 140.4, 140.3, 136.3, 130.5, 126.9, 126.9, 126.1, 123.9, 123.3, 121.4, 117.9, 114.8, 106.8, 77.4, 77.2, 76.9, 27.7, 23.0. HRMS (ESI) m/z: $[M + H]^+$ calcd for $C_{32}H_{31}N_6Br_2$, 657.0971; found, 657.0974.

N^3,*N*$^{3'}$-bis(4-chlorophenyl)-[2,2'-biimidazo[1,2-*a*]pyridine]-3,3'-diamine(**3bj**)

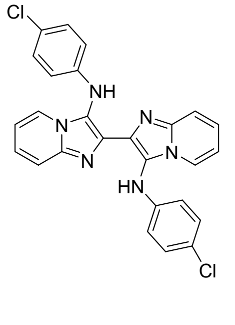

Follow general procedure I on 1 mmol scale. Yellow solid (75 mg, 31%), m.p.: 259.3-259.6°C; TCL (Dichloromethane : Methanol = 97 : 3): R_f = 0.51;^{1}H NMR (500 MHz, $CDCl_3$) δ 7.56 (dd, J = 6.9, 1.8 Hz, 2H), 7.53 (dd, J = 8.7, 2.2 Hz, 2H), 7.32 (s, 2H), 7.13 (ddd, J = 8.7, 6.7, 1.3 Hz, 2H), 7.08

(dd, J = 8.8, 1.4 Hz, 4H), 6.73 – 6.99 (m, 2H), 6.42 – 6.39 (m, 4H).^{13}C NMR (126 MHz, $CDCl_3$) δ 142.9, 142.3, 131.4, 129.3, 124.9, 124.2, 123.1, 121.3, 117.5, 116.2, 112.0. HRMS (ESI) m/z: $[M + H]^+$ calcd for $C_{26}H_{19}Cl_2N_6$, 485.1043; found, 485.1039.

N^5,*N*$^{5'}$-bis(2-isopropylphenyl)-[6,6'-biimidazo[2,1-*b*]thiazole]-5,5'-diamine(**3bk**)

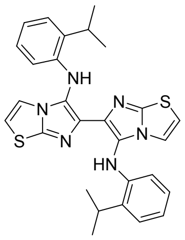

Follow general procedure I on 1 mmol scale. Yellow solid (59 mg, 23%), m.p.: 231.3-231.4°C; TCL (Dichloromethane : Methanol = 97 : 3): R_f = 0.51;^{1}H NMR (500 MHz, $CDCl_3$) δ 7.35 – 7.31 (m, 2H), 7.26 (s, 2H), 7.05 – 6.98 (m, 4H), 6.95 – 6.92 (m, 2H), 6.70 (d, J = 4.2 Hz, 2H), 6.39 (d, J = 4.2 Hz, 2H), 3.41 (q, J = 6.8 Hz, 2H), 1.47 (d, J = 6.8 Hz, 12H).^{13}C NMR (126 MHz, $CDCl_3$) δ 144.5, 141.4, 135.9, 130.5, 126.6, 125.7, 123.8, 120.8, 117.7, 114.7, 111.1, 27.5, 22.8. HRMS (ESI) m/z: $[M + H]^+$ calcd for $C_{28}H_{29}N_6S_2$, 513.1890; found, 513.1888.

N^5,*N*$^{5'}$-bis(4-chlorophenyl)-[6,6'-biimidazo[2,1-*b*]thiazole]-5,5'-diamine(**3bl**)

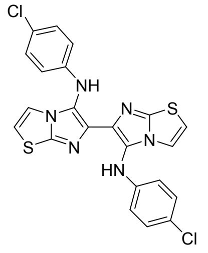

Follow general procedure I on 1 mmol scale. Yellow solid (25 mg, 10%), m.p.: 250.7-260.0°C; TCL (Dichloromethane : Methanol = 97 : 3): R_f = 0.51;^{1}H NMR (500 MHz, DMSO) δ 8.06 (s, 2H), 7.41 (d, J = 4.5 Hz, 2H), 7.21 (d, J = 4.5 Hz, 2H), 7.12 (d, J = 7.6 Hz, 4H), 6.49 (d, J = 7.6 Hz, 4H).^{13}C NMR (126 MHz, DMSO) δ 145.6, 145.4, 133.5, 129.2, 122.3, 122.0, 118.3, 115.8, 113.7. HRMS (ESI) m/z: $[M + H]^+$ calcd for $C_{22}H_{15}Cl_2N_6S_2$, 497.0171; found, 497.0172.

N^3,*N*$^{3'}$-bis(2-methoxyphenyl)-[2,2'-biimidazo[1,2-*a*]pyridine]-3,3'-diamine(**3bm**)

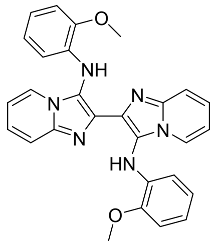

Follow general procedure I on 1 mmol scale. Yellow solid (98 mg, 41%), m.p.: 244.1-244.6°C; TCL (Dichloromethane : Methanol = 97 : 3): R_f = 0.51;^{1}H NMR (500 MHz, $CDCl_3$) δ 7.70 (dd, J = 6.8, 1.2 Hz, 2H), 7.64 (dd, J = 8.7, 1.2 Hz, 2H), 7.21 – 7.13 (m, 2H), 7.03 (s, 2H), 6.91 – 6.83 (m, 2H), 6.82 – 6.76 (m, 2H), 6.76 – 6.70 (m, 2H), 6.68 – 6.60 (m, 2H), 6.04 (dd, J = 7.9, 2.0 Hz, 2H), 3.95 (s, 6H).^{13}C NMR (126 MHz, $CDCl_3$) δ 147.8, 142.7, 133.9, 132.5, 123.9, 123.2, 121.1, 120.9, 119.5, 117.9, 112.9, 111.7, 110.2, 55.7. HRMS (ESI) m/z: $[M + H]^+$ calcd for $C_{28}H_{25}N_6O_2$, 477.2034; found, 477.2028.

6,6'-dibromo-*N*^{3},*N*$^{3'}$-bis(2-methoxyphenyl)-[2,2'-biimidazo[1,2-*a*]pyridine]-3,3'-diamine(**3bn**)

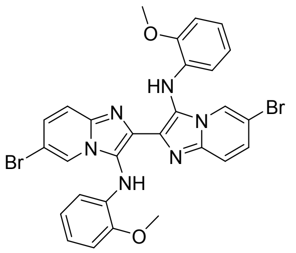

Follow general procedure I on 1 mmol scale. Yellow solid (161 mg, 51%), m.p.: 260.5-262.3 °C; TCL (Dichloromethane : Methanol = 97 : 3): R_f = 0.28; ^{1}H NMR (500 MHz, $CDCl_3$) δ 7.83 (d, J = 2.0 Hz, 2H), 7.48 (d, J = 8.6 Hz, 2H), 7.21 (dd, J = 8.6, 2.0 Hz, 2H), 6.97 (s, 2H), 6.87 (dd, J = 8.2, 1.4 Hz, 2H), 6.81 – 6.78 (m, 2H), 6.67 – 6.63 (m, 2H), 5.98 (dd, J = 7.8, 1.5 Hz, 2H), 3.95 (s, 6H). ^{13}C NMR (126 MHz, $CDCl_3$) δ 147.9, 141.1, 133.6, 132.9, 127.6, 123.3, 121.6, 121.3, 120.1, 118.7, 112.9, 110.5, 106.9, 55.9. HRMS (ESI) m/z: $[M + H]^+$ calcd for $C_{28}H_{23}O_2N_6Br_2$, 633.0244; found, 633.0248

3,3'-bis(cyclohexylamino-d)-[2,2'-biimidazo[1,2-*a*]pyridine]-6,6'-dicarboxylic acid(**3bo**)

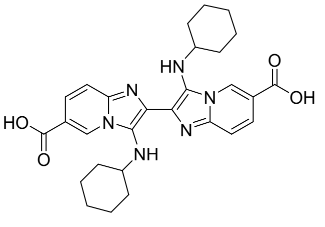

Follow general procedure III on 0.2 mmol scale. Yellow solid (93 mg, 90%), m.p.: 236.8-237.0°C; TCL (Dichloromethane : Methanol = 97 : 3): R_f= 0.51;1H NMR (500 MHz, DMSO) δ 8.74 (s, 2H), 7.57 (dd, J = 8.8, 2.0 Hz, 4H), 3.14 – 3.11 (m, 2H), 1.80– 1.78 (m, 4H), 1.67 – 1.63 (m, 4H), 1.49 – 1.46 (m, 2H), 1.30 – 1.13 (m, 10H).13C NMR (126 MHz, DMSO) δ 166.5, 151.7, 141.2, 130.4, 128.9, 126.8, 123.3, 116.5, 55.9, 33.8, 25.9, 24.6. HRMS (ESI) m/z: $[M + H]^+$ calcd for $C_{28}H_{33}N_6O_4$, 517.2558; found, 517.2558.

7'-bromo-6-chloro-*N*3,*N*3'-bis(2-ethylphenyl)-[2,2'-biimidazo[1,2-*a*]pyridine]-3,3'-diamine(**3bp**)

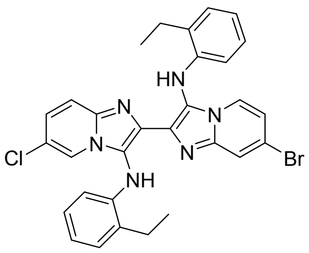

The compound synthesized from 2-amino-5-chloropyridine (0.5 mmol), 2-amino-4-bromopyridine (0.5 mmol), 2,2-dimethoxyacetaldehyde(60 wt. % in H_2O, 0.85 mmol), $Sc(OTf)_3$ (0.1 mmol) and isocyanide (1 mmol) Follow general procedure I. Yellow solid (76 mg, 26%), m.p.: 251.0-252.4 °C; TCL (Ethyl acetate : Petroleum ether = 3 : 7): R_f = 0.22; ^{1}H NMR (500 MHz, $CDCl_3$) δ 7.66 (d, *J* = 2.2 Hz, 1H), 7.61 (d, *J* = 2.2 Hz, 1H), 7.54 (s, 1H), 7.50 (s, 1H), 7.45 – 7.38 (m, 2H), 7.28 – 7.24 (m, 2H), 7.09 (dd, *J* = 8.4, 2.2 Hz, 1H), 6.96 – 6.92 (m, 2H), 6.93 – 6.88 (m, 2H), 6.80 (dd, *J* = 7.2, 2.0 Hz, 1H), 6.00 (dd, *J* = 6.1, 2.1 Hz, 2H), 2.96 – 2.90 (m, 4H), 1.58 – 1.53 (m, 6H). ^{13}C NMR (126 MHz, $CDCl_3$) δ 141.9, 141.1, 141.1, 140.3, 131.8, 131.7, 130.9, 130.1, 129.2, 127.1, 127.1, 124.9, 123.8, 123.8, 123.7, 121.2, 121.2, 121.2, 120.3, 119.6, 117.7, 117.3, 115.7, 114.6, 114.3, 24.6, 24.6, 14.1. HRMS (ESI) m/z: $[M + H]^+$ calcd for $C_{30}H_{27}N_6BrCl$, 585.1164; found, 585.1164.

*N*3,*N*3'-dibenzyl-7-bromo-7'-(trifluoromethyl)-[2,2'-biimidazo[1,2-*a*]pyridine]-3,3'-diamine(**3bq**)

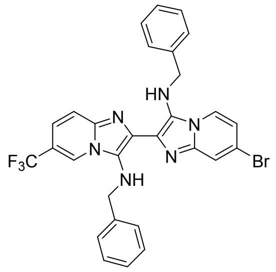

The compound synthesized from 2-amino-4-fluooropyridine (0.5 mmol), 2-amino-4-trifluoromethylpyridine (0.5 mmol), 2,2-dimethoxyacetaldehyde(60 wt. % in H_2O, 0.85 mmol), $Sc(OTf)_3$ (0.1 mmol) and isocyanide (1 mmol) Follow general procedure I. Yellow solid (76 mg, 28%), m.p.: 251.0-252.4 °C; TCL (Dichloromethane : Methanol = 97 : 3): R_f= 0.50; ^{1}H NMR (500 MHz, $CDCl_3$) δ 8.26 (d, *J* = 2.0 Hz, 1H), 8.09 (d, *J* = 2.0 Hz, 1H), 7.50 (*d*, J = 8.7 Hz, 1H), 7.48 – 7.45 (m, 2H), 7.43 – 7.38 (m, 2H), 7.37 – 7.35 (m, 1H), 7.35 – 7.27 (m, 6H), 7.18 (dd, *J* = 8.6, 1.9 Hz, 1H), 7.14 (dd, *J* = 8.6, 1.9 Hz, 1H), 5.61 (s, 2H), 4.29 – 4.22 (m, 4H).^{13}C NMR (126 MHz, $CDCl_3$) δ 141.9, 141.1, 141.1, 140.3, 131.8, 131.7, 130.9, 130.1, 129.2, 127.1, 127.1, 124.9, 123.8, 123.8, 123.7, 121.2, 121.2, 121.2, 120.3, 119.6, 117.7, 117.3, 115.7, 114.6, 114.3, 24.6, 24.6, 14.1. HRMS (ESI) m/z: $[M + H]^+$ calcd for $C_{29}H_{23}$ N_6BrF_3, 591.1114; found, 591.1114.

*N*3,*N*3'-dibenzyl-6,6'-bis(trifluoromethyl)-[2,2'-biimidazo[1,2-a]pyridine]-3,3'-diamine(**3br**)

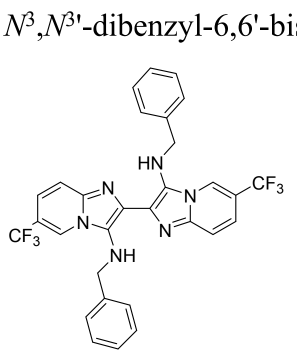

Yellow solid (52 mg, 36%), m.p.: 278.0-280.6 °C; TCL (Dichloromethane : Methanol = 97 : 3): R_f = 0.58; ^{1}H NMR (500 MHz, TFA-d) δ 8.24 (d, *J* = 7.2 Hz, 2H), 7.94 (d, *J* = 1.6 Hz, 2H), 7.57 (dd, *J*

= 7.2, 1.6 Hz, 2H), 7.11 – 6.78 (m, 10H), 4.12 (s, 4H). ^{13}C NMR (126 MHz, TFA-d) δ 137.93 , 136.92 , 131.96 , 130.72 , 128.35 , 127.94 , 127.75 , 124.51 , 122.54(d, J =10.5 Hz), 114.73, 114.44 (q, J = 283.5 Hz), 112.38, 51.74. HRMS (ESI) m/z: $[M + H]^+$ calcd for $C_{30}H_{23}N_6F_6$, 581.1810; found, 581.1814.

*N*3,*N*3'-dibenzyl-7,7'-dibromo-[2,2'-biimidazo[1,2-a]pyridine]-3,3'-diamine(**3bs**)

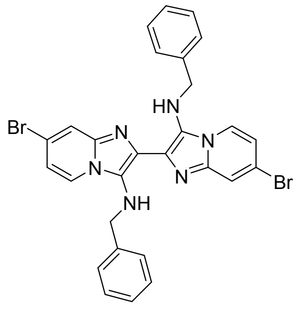

Yellow solid (12 mg, 8%), m.p.: 236.0-237.9 °C; TCL (Dichloromethane : Methanol = 97 : 3): R_f = 0.38; ^1H NMR (500 MHz, TFA-d) δ 8.68 (d, J = 7.5 Hz, 2H), 8.12 (dd, J = 7.5, 1.5 Hz, 2H), 7.97 (d, J = 1.5 Hz, 2H), 7.07 – 6.85 (m, 10H), 4.22 (s, 4H). ^{13}C NMR (126 MHz, TFA-d) δ 138.12 , 136.85 , 132.34 , 131.46 , 128.51 , 128.12 , 127.56 , 123.65, 114.32 (q, J = 283.4 Hz), 113.44, 112.70, 52.00. HRMS (ESI) m/z: $[M + H]^+$ calcd for $C_{28}H_{23}Br_2N_6$, 601.0273; found, 601.0275.

^1H NMR spectrum of **3aa** (500 MHz, $CDCl_3$)

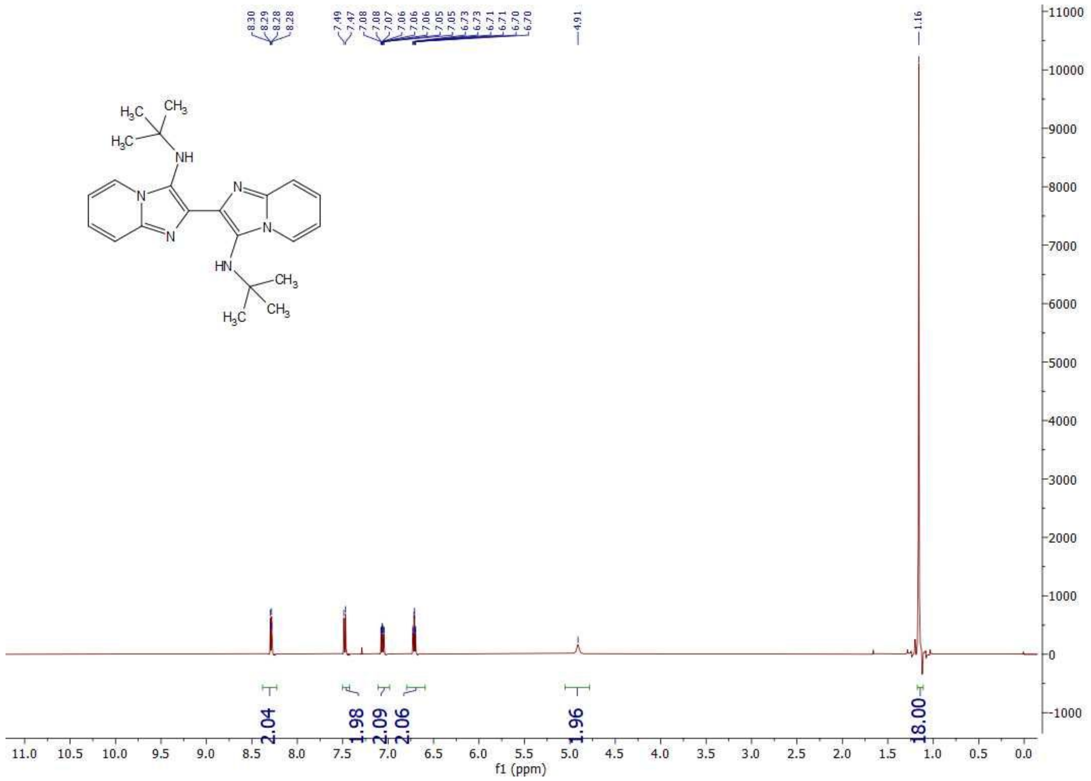

$^{13}C\{^{1}H\}$ NMR spectrum of **3aa** (126 MHz, $CDCl_3$)

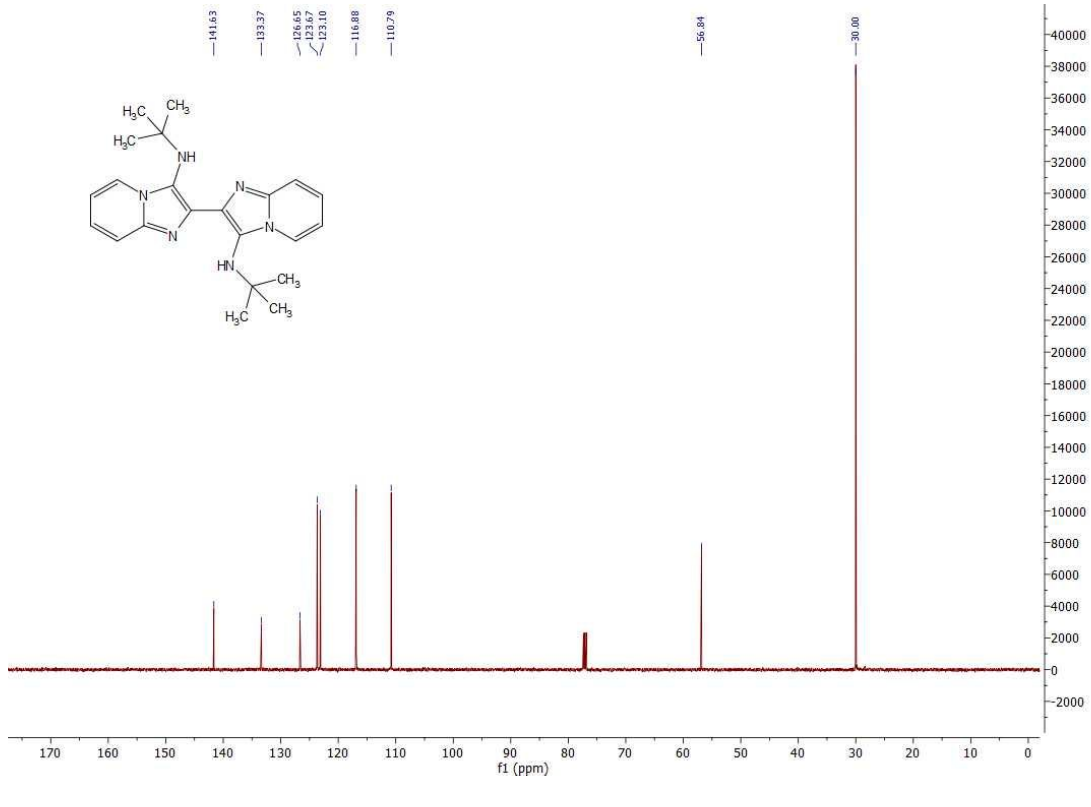

^{1}H NMR spectrum of **3ab** (500 MHz, $CDCl_3$)

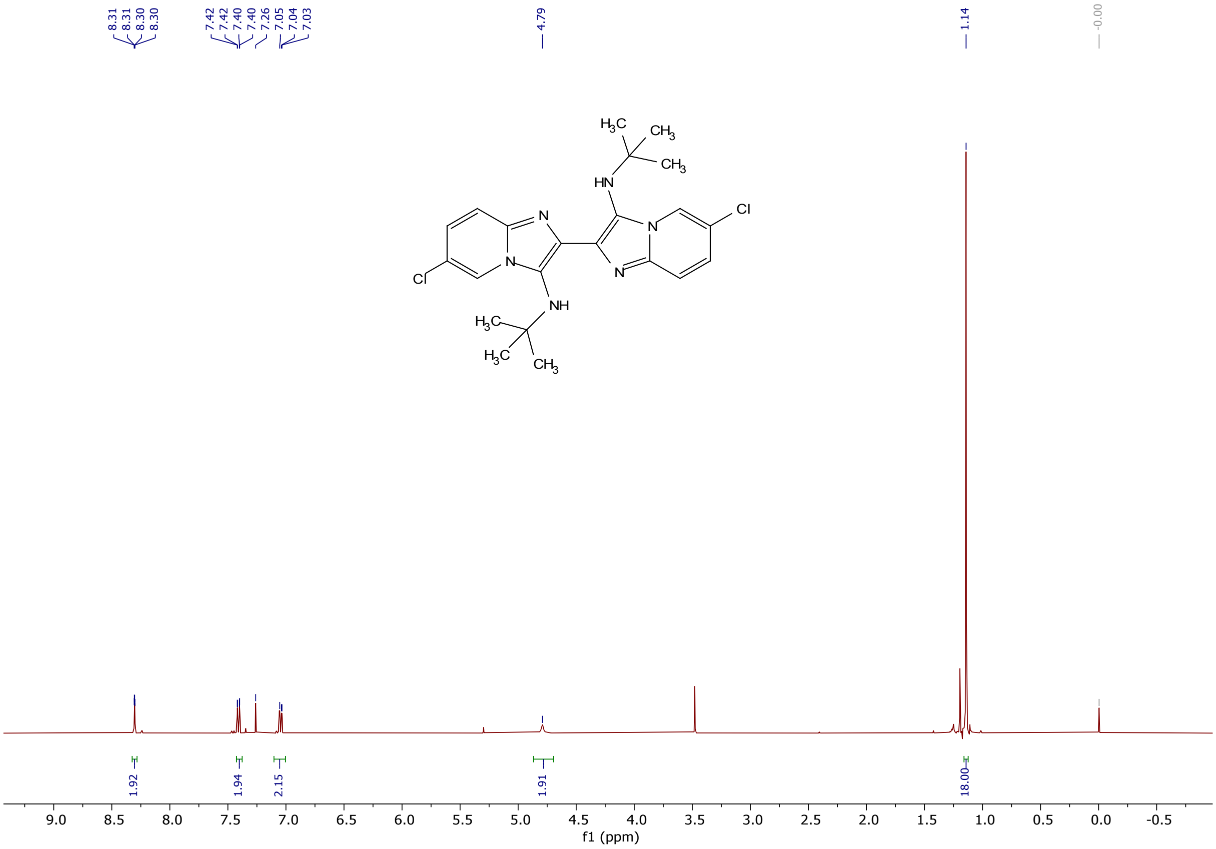

$^{13}C\{^{1}H\}$ NMR spectrum of **3ab** (126 MHz, $CDCl_3$)

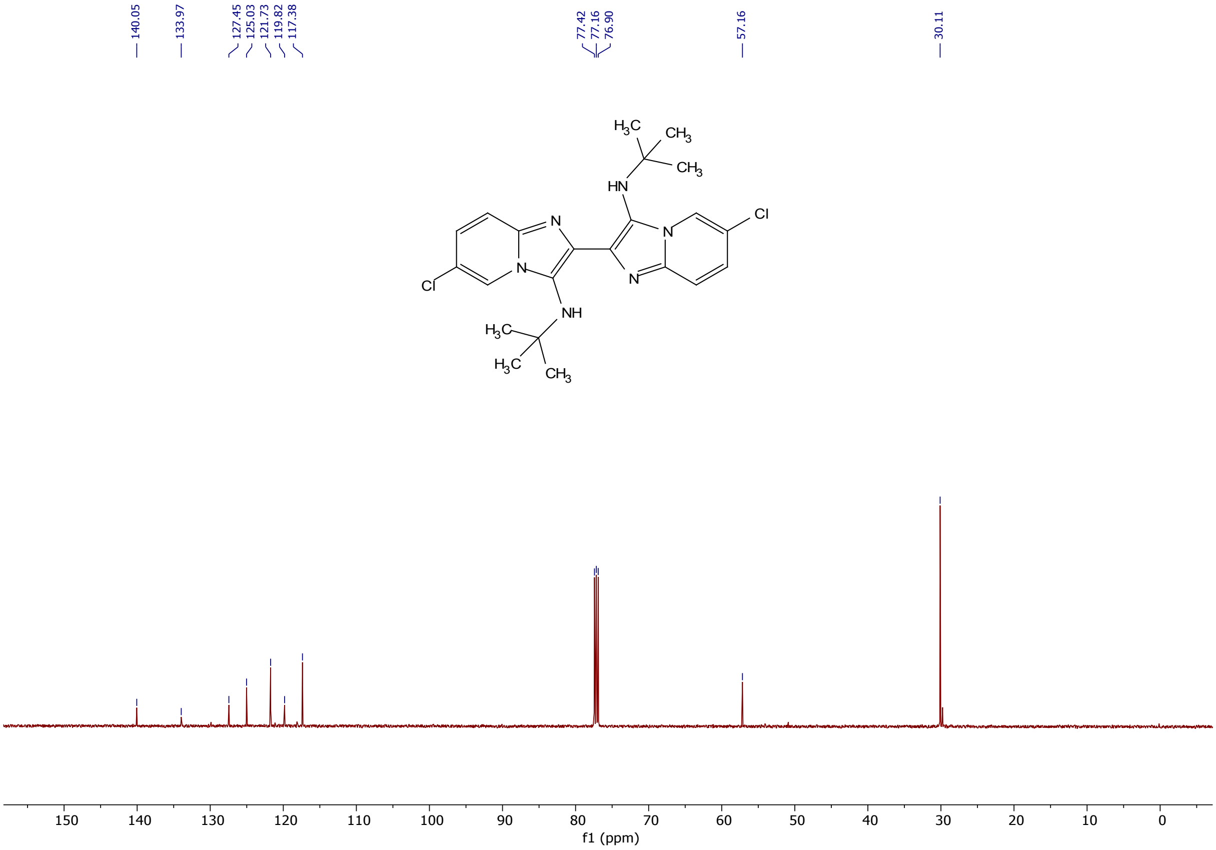

^{1}H NMR spectrum of **3ac** (500 MHz, $CDCl_3$)

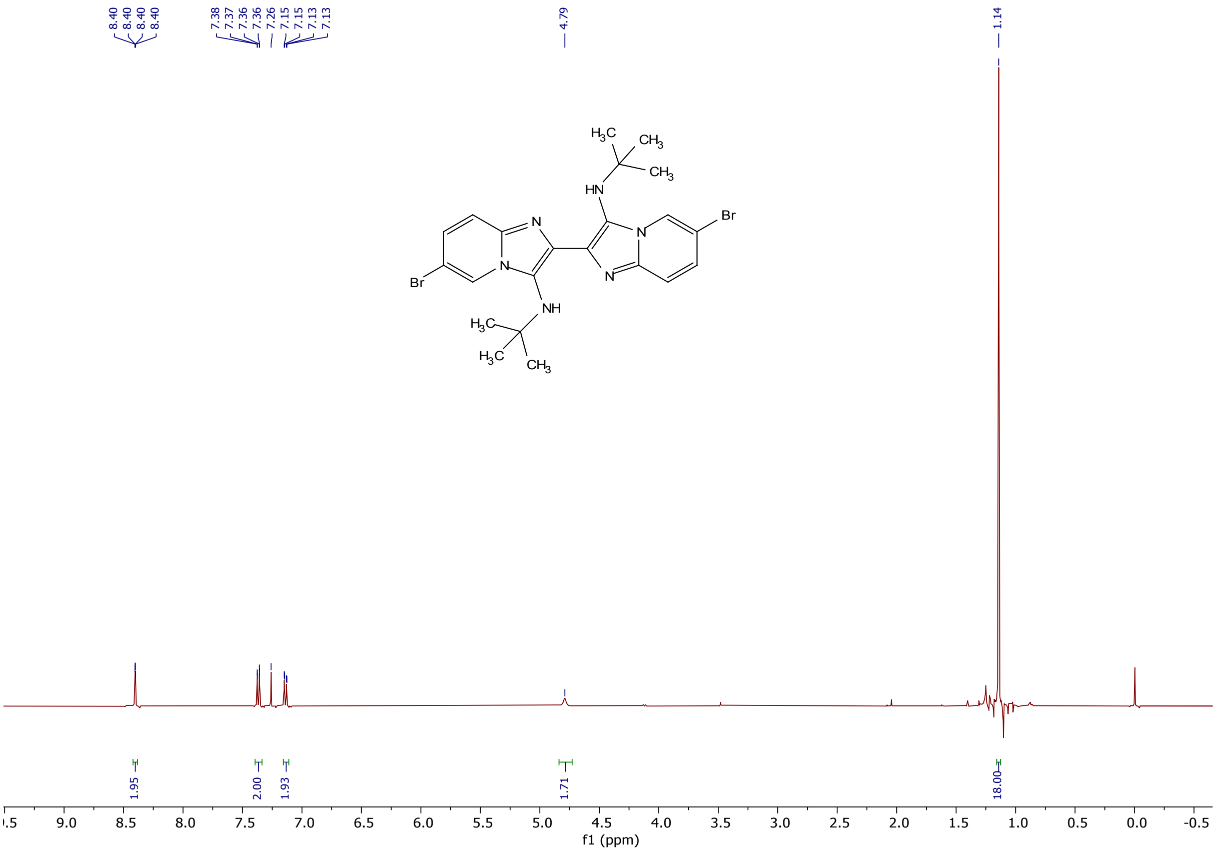

$^{13}C\{^1H\}$ NMR spectrum of **3ac** (126 MHz, $CDCl_3$)

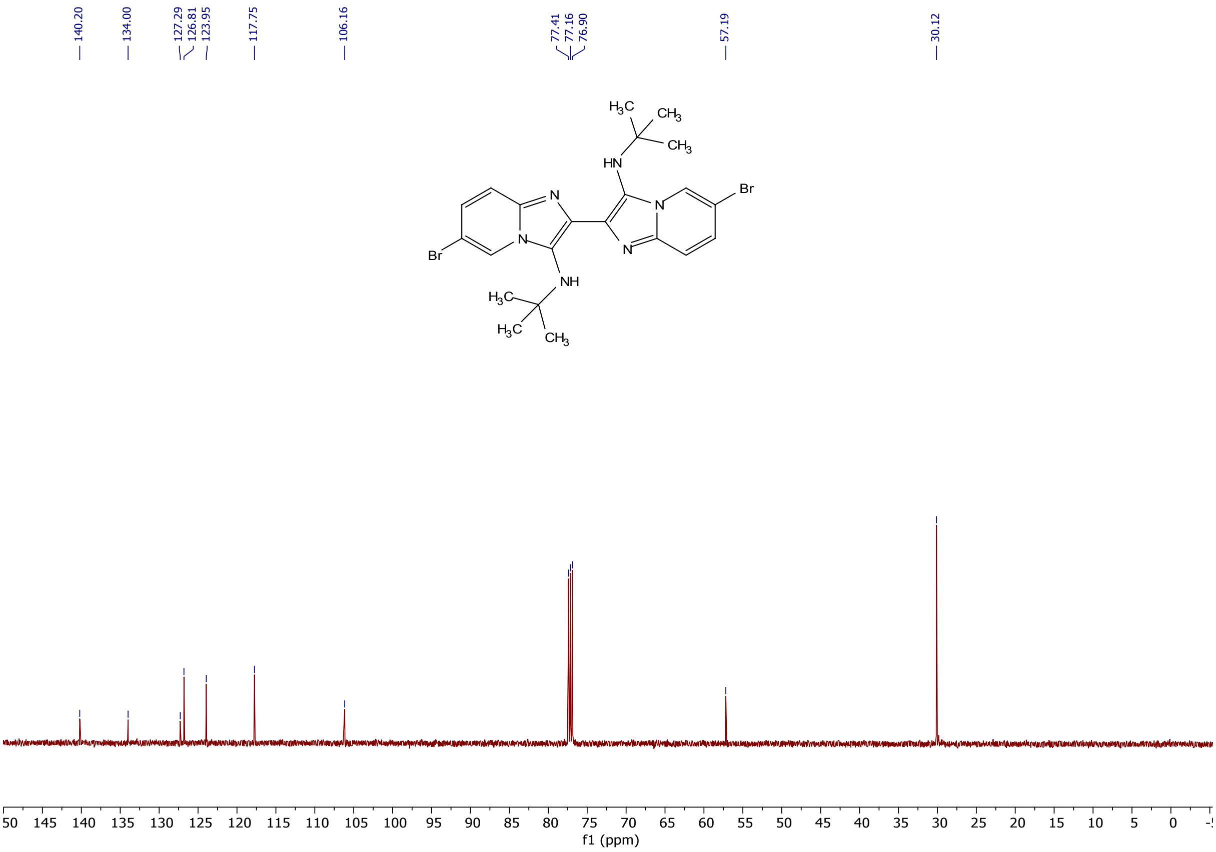

^1H NMR spectrum of **3ad** (500 MHz, DMSO-d_6)

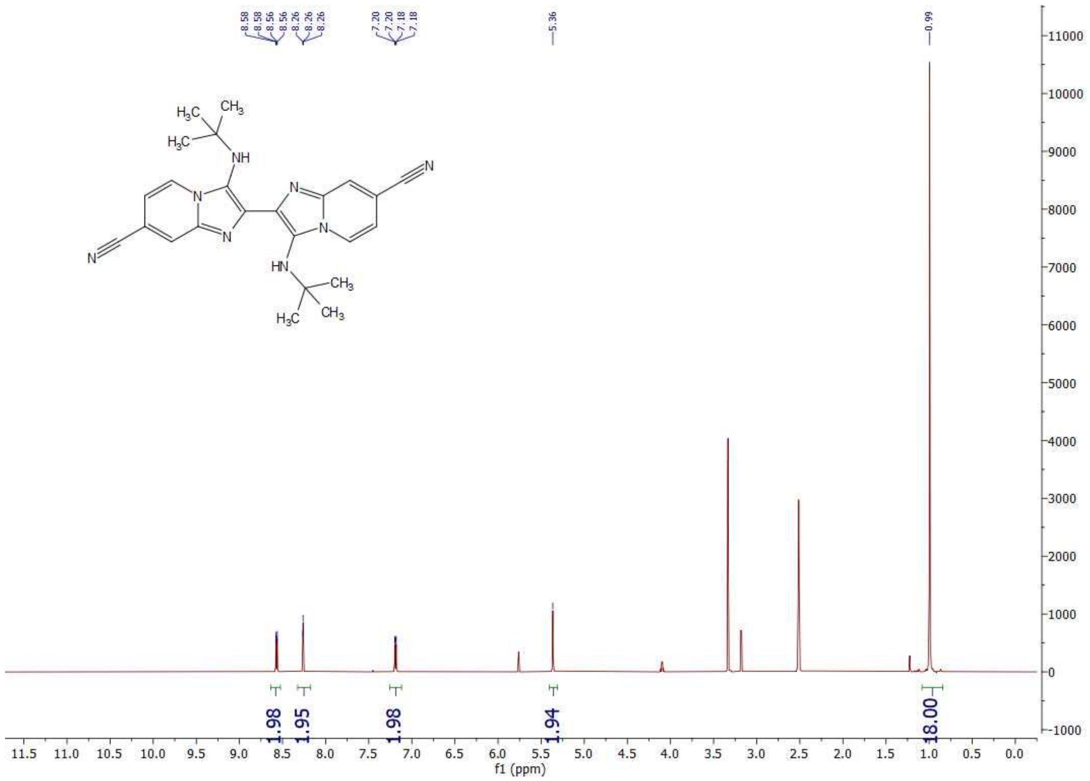

$^{13}C\{^1H\}$ NMR spectrum of **3ad** (126 MHz, DMSO-d_6)

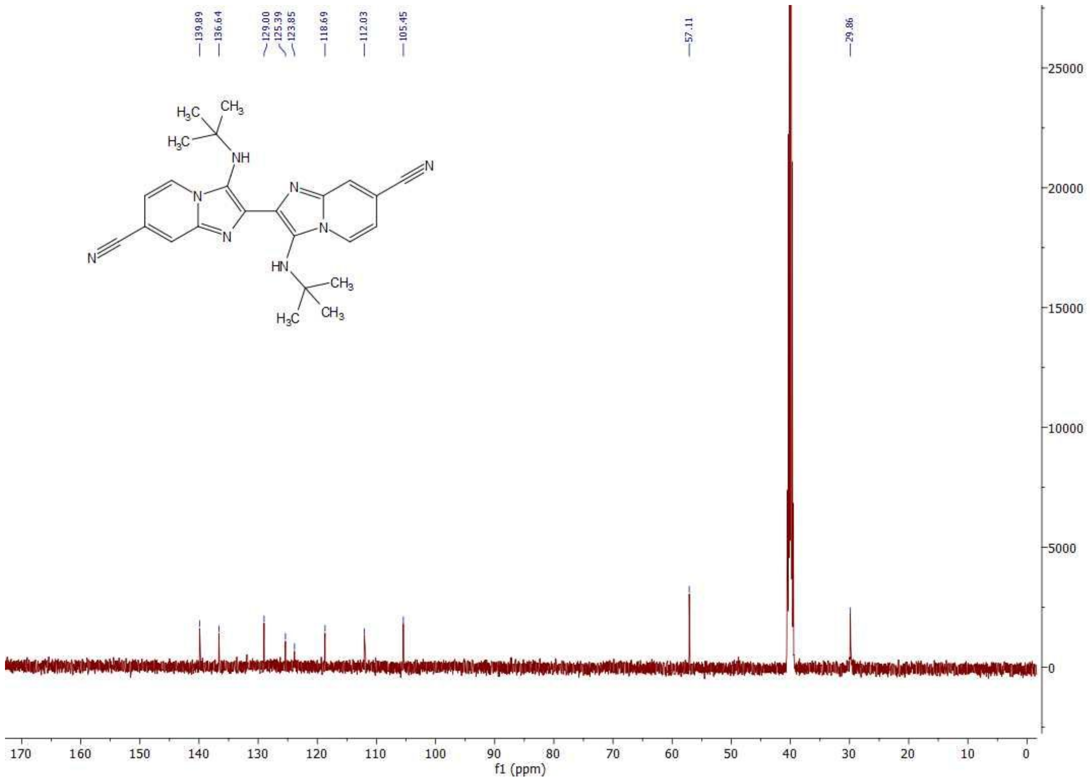

^1H NMR spectrum of **3ae** (500 MHz, $CDCl_3$)

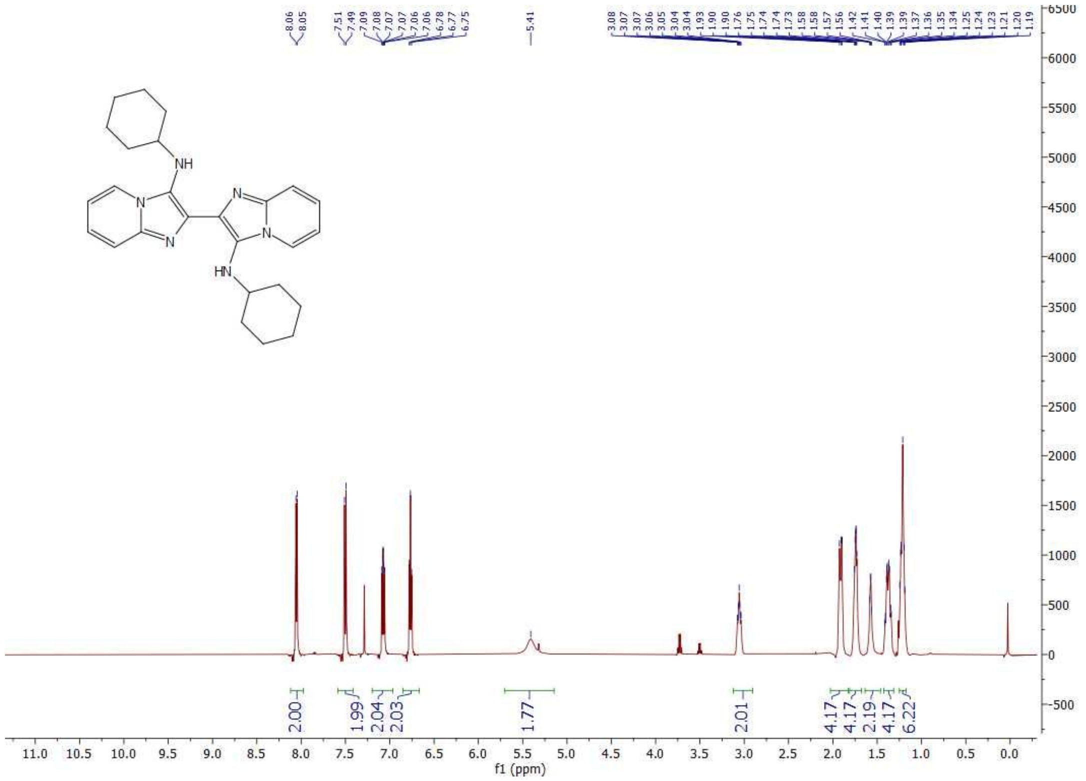

$^{13}C\{^{1}H\}$ NMR spectrum of **3ae** (126 MHz, $CDCl_3$)

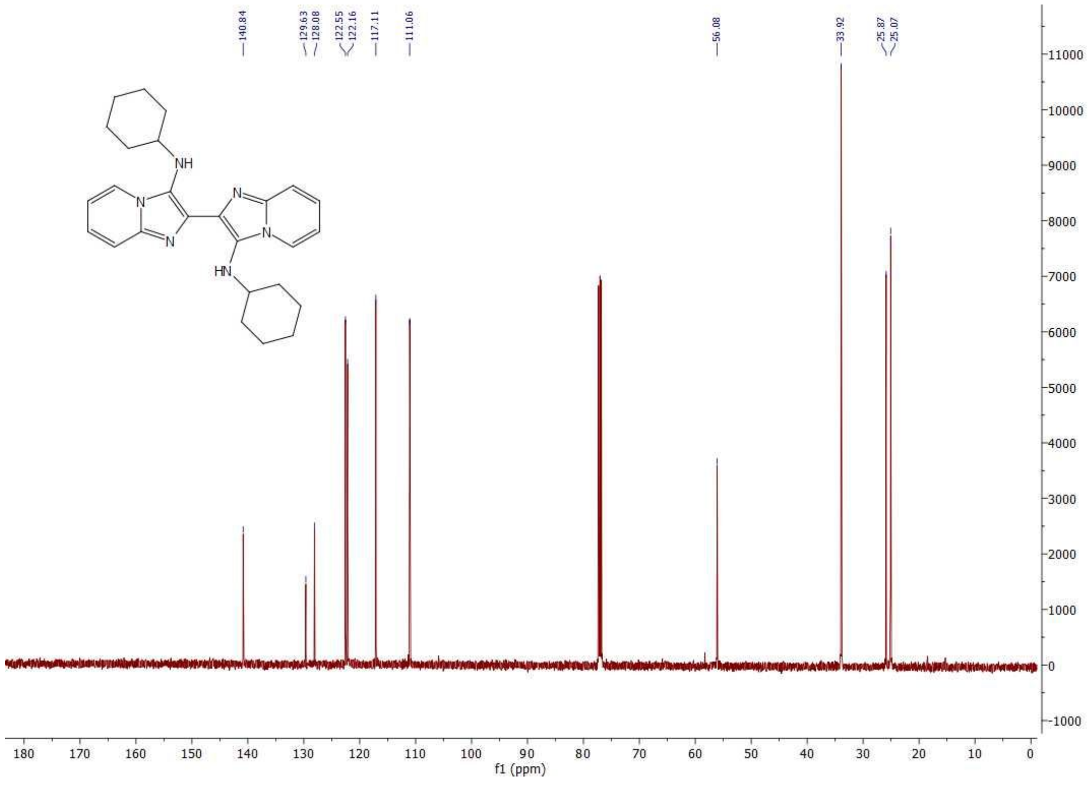

^{1}H NMR spectrum of **3af** (500 MHz, $CDCl_3$)

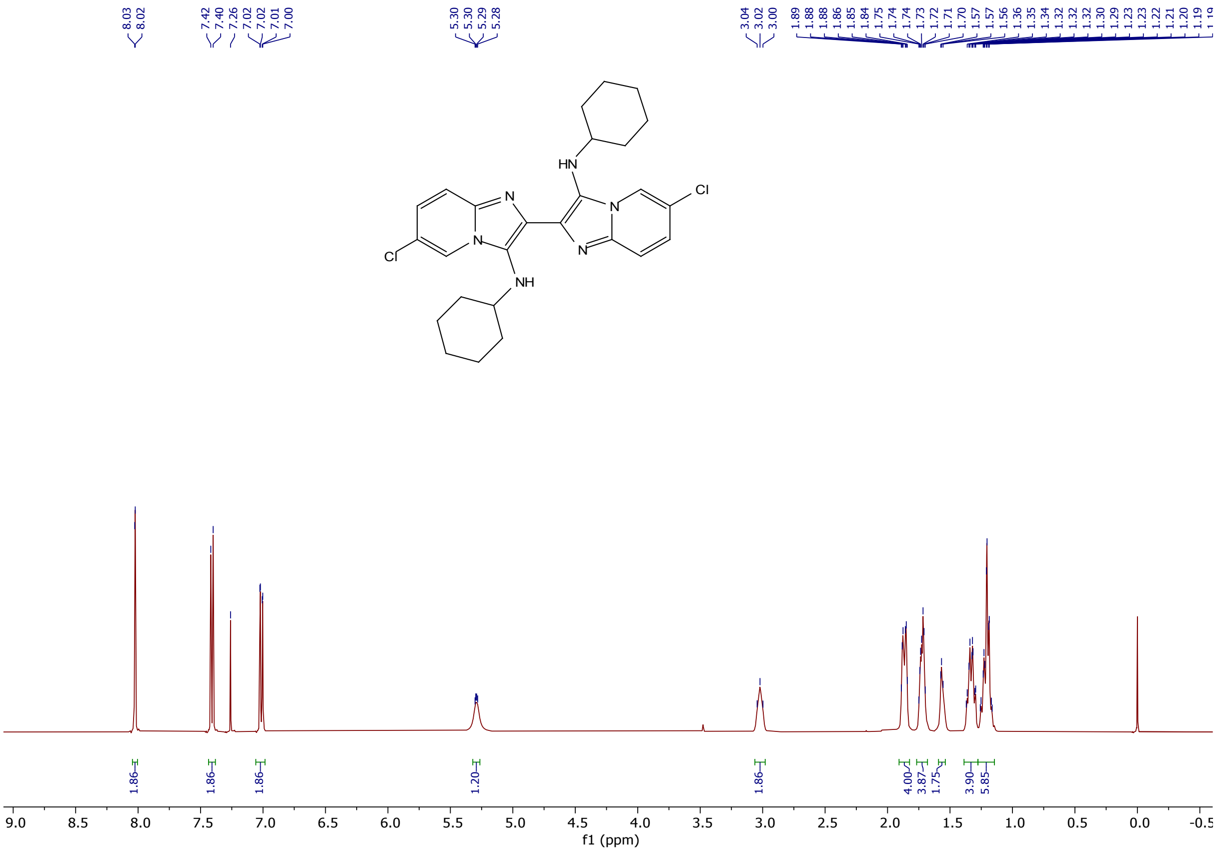

$^{13}C\{^{1}H\}$ NMR spectrum of **3af** (126 MHz, $CDCl_3$)

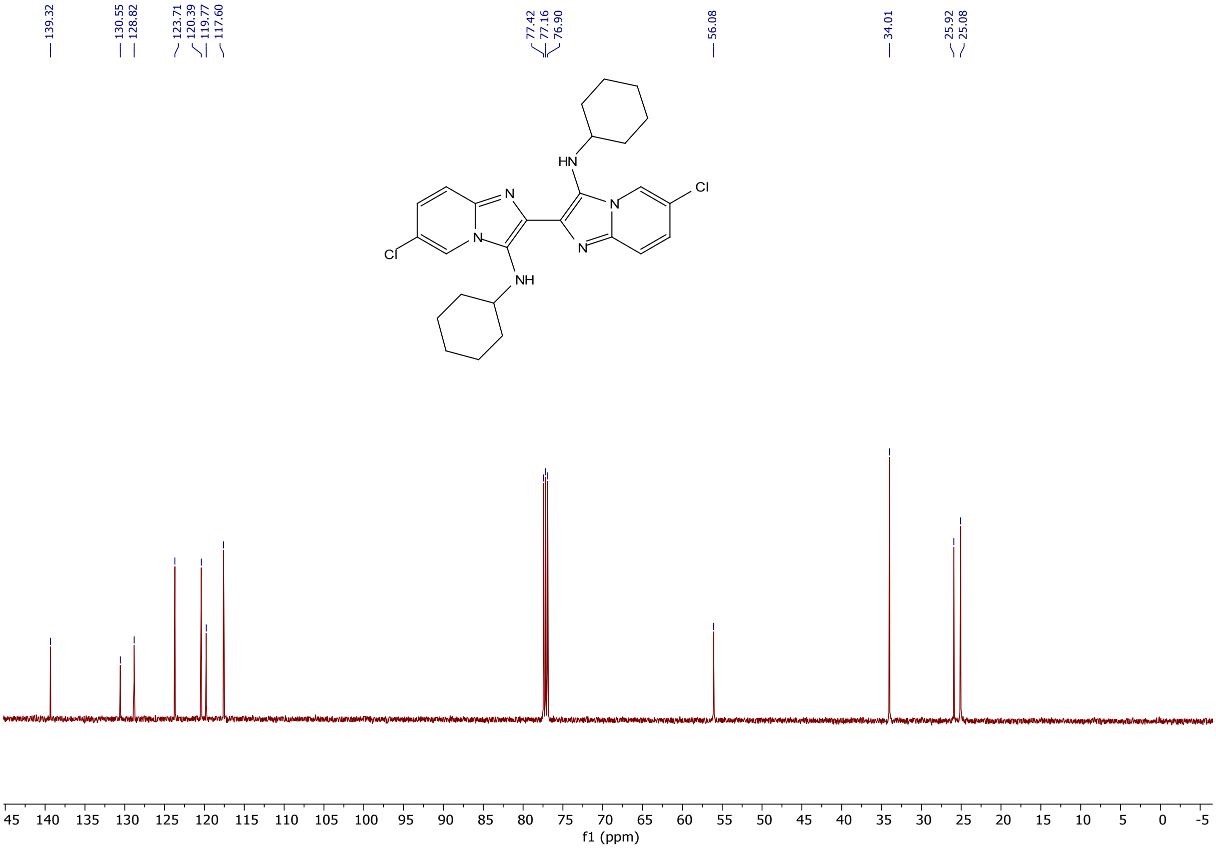

^{1}H NMR spectrum of **3ag** (500 MHz, $CDCl_3$)

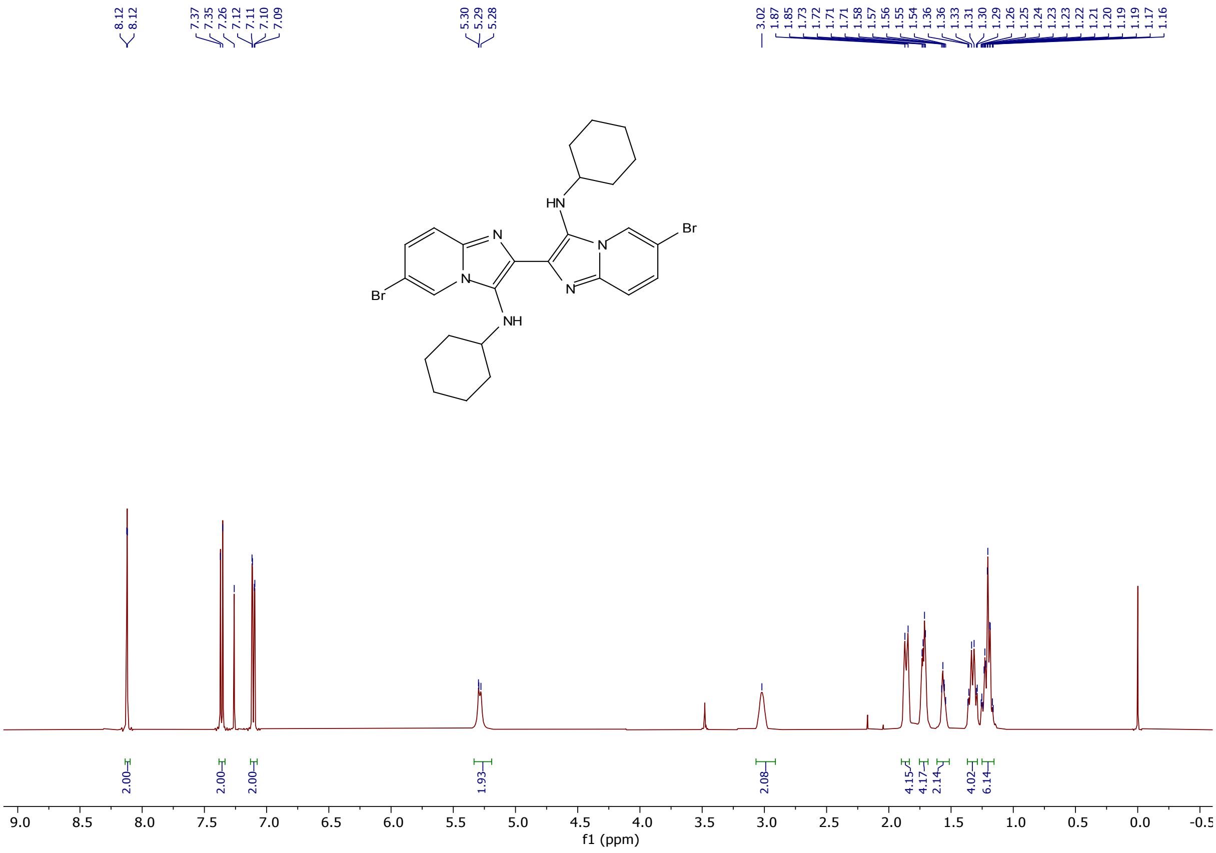

$^{13}C\{^1H\}$ NMR spectrum of **3ag** (126 MHz, $CDCl_3$)

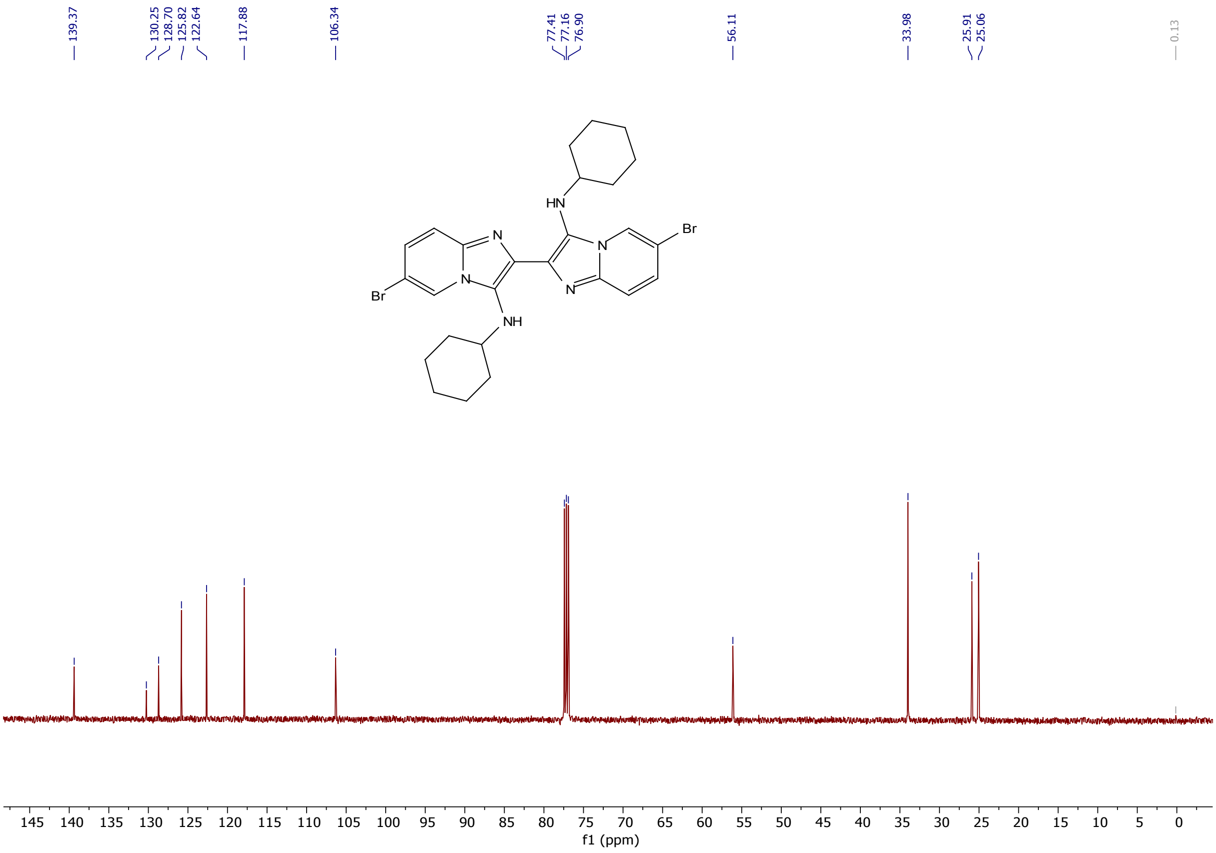

^1H NMR spectrum of **3ah** (500 MHz, $CDCl_3$)

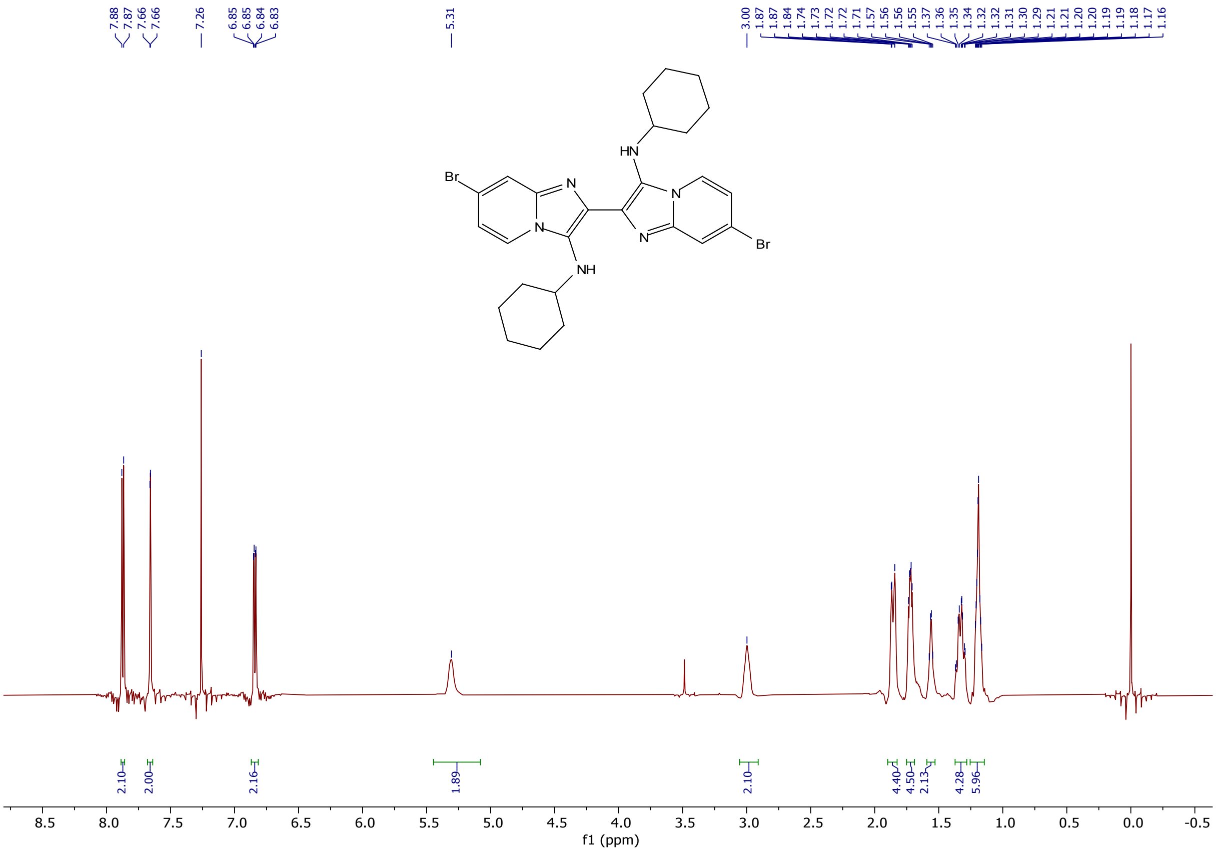

$^{13}C\{^{1}H\}$ NMR spectrum of **3ah** (126 MHz, $CDCl_3$)

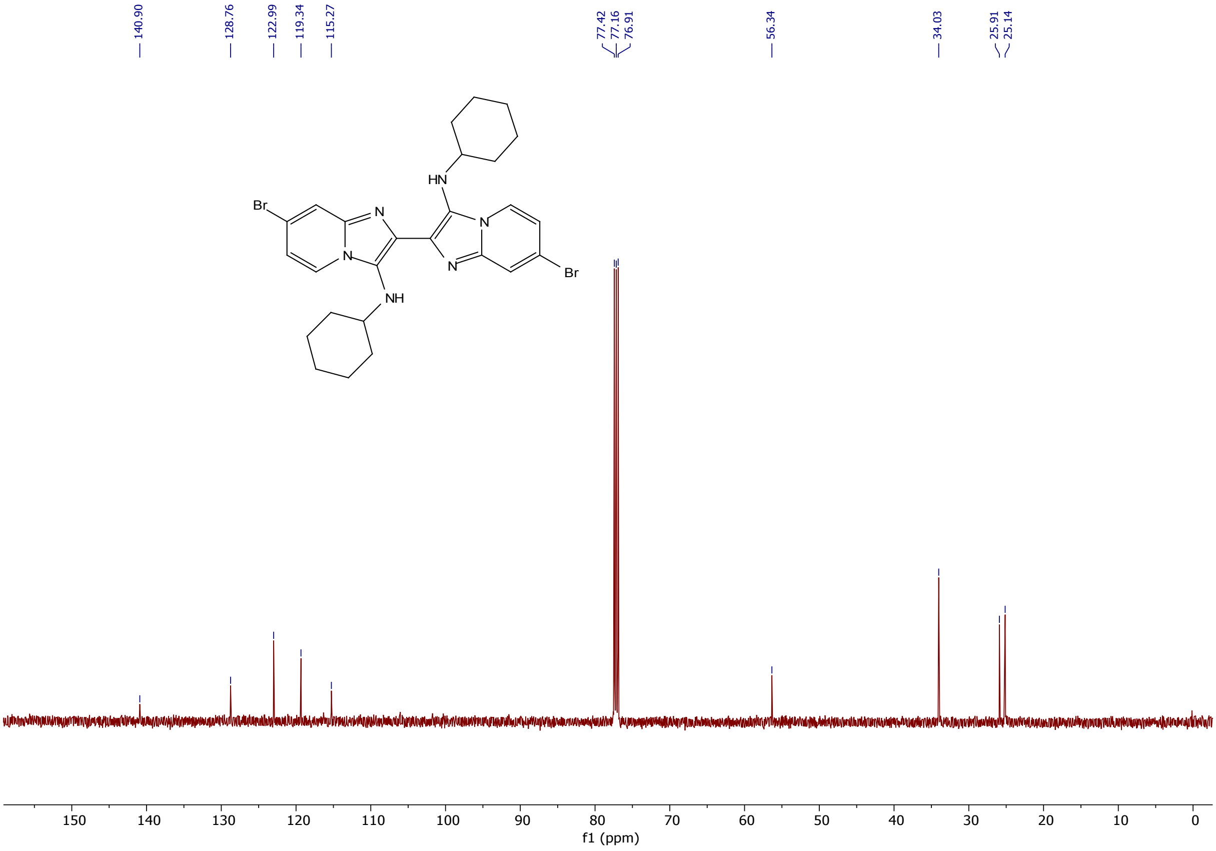

^{1}H NMR spectrum of **3ai** (500 MHz, $CDCl_3$)

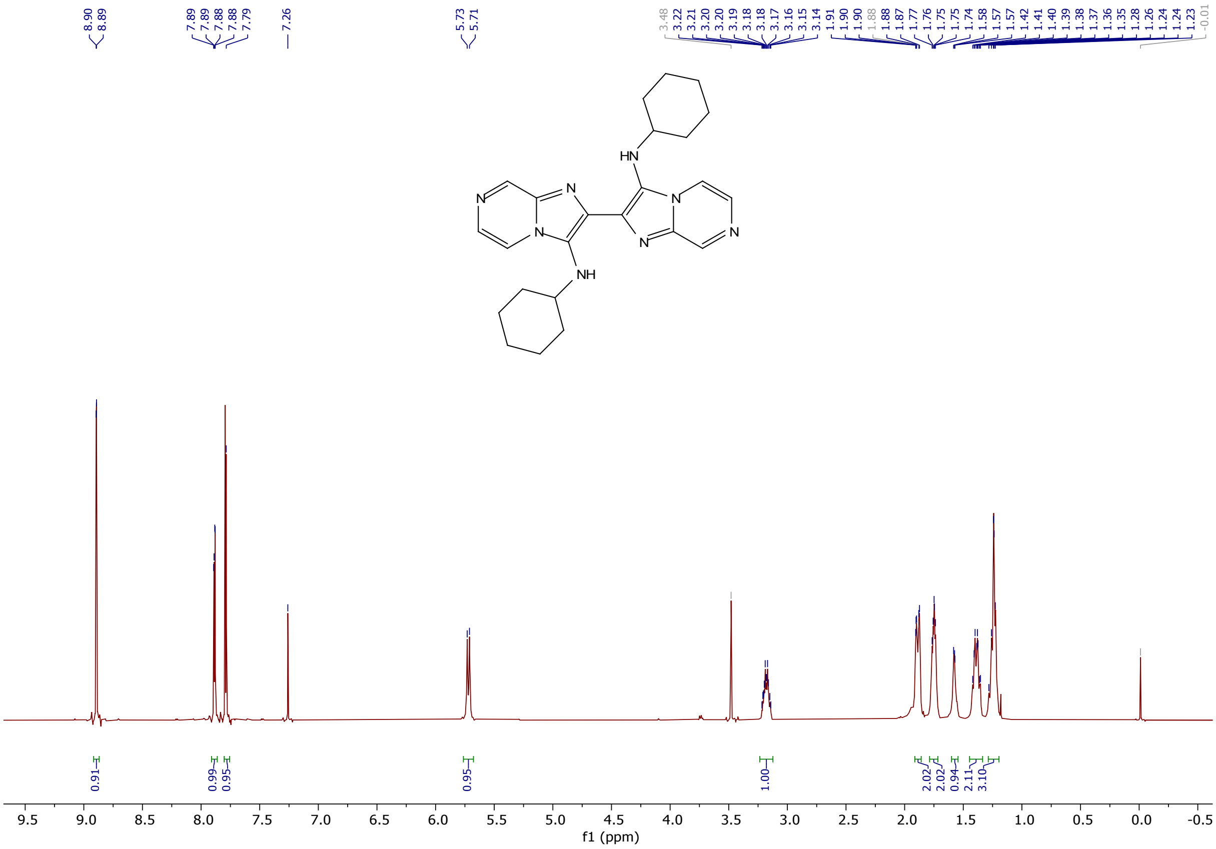

$^{13}C\{^{1}H\}$ NMR spectrum of **3ai** (126 MHz, $CDCl_3$)

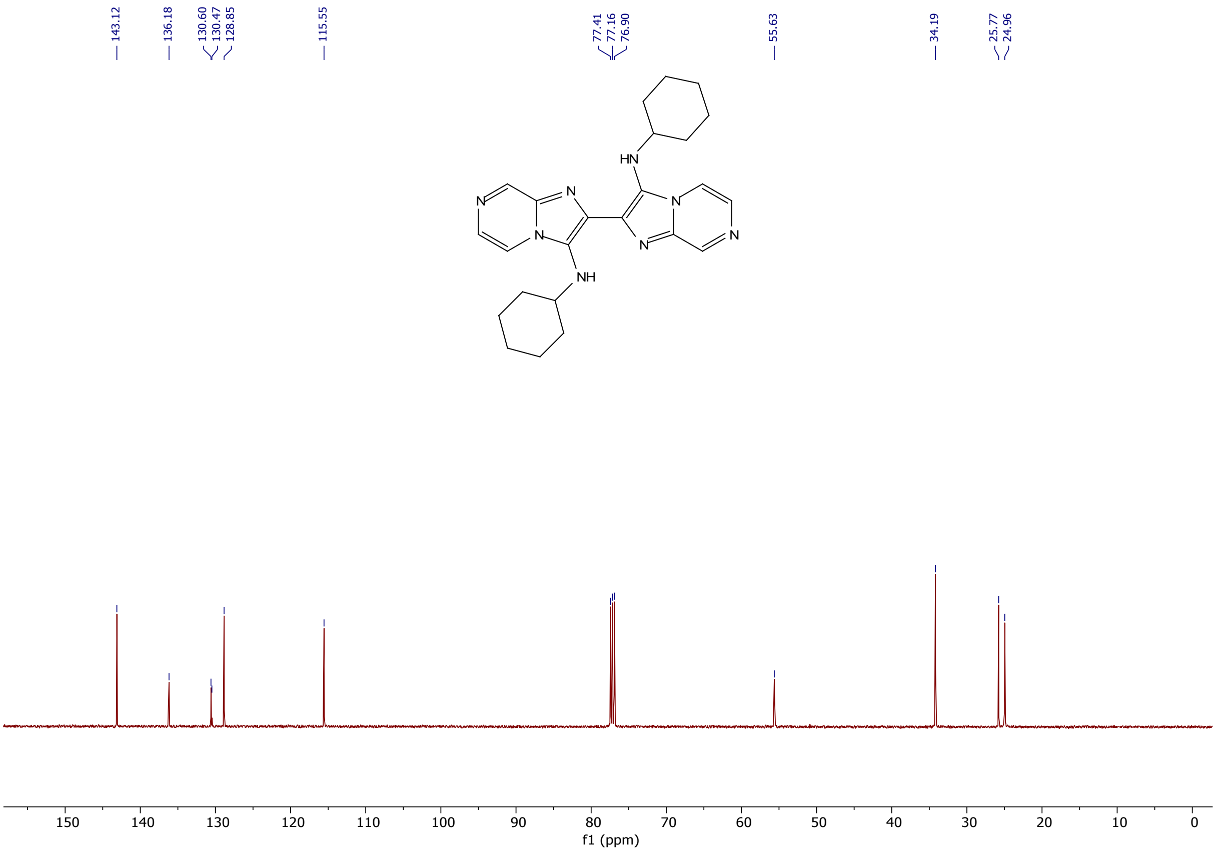

^{1}H NMR spectrum of **3aj** (500 MHz, $CDCl_3$)

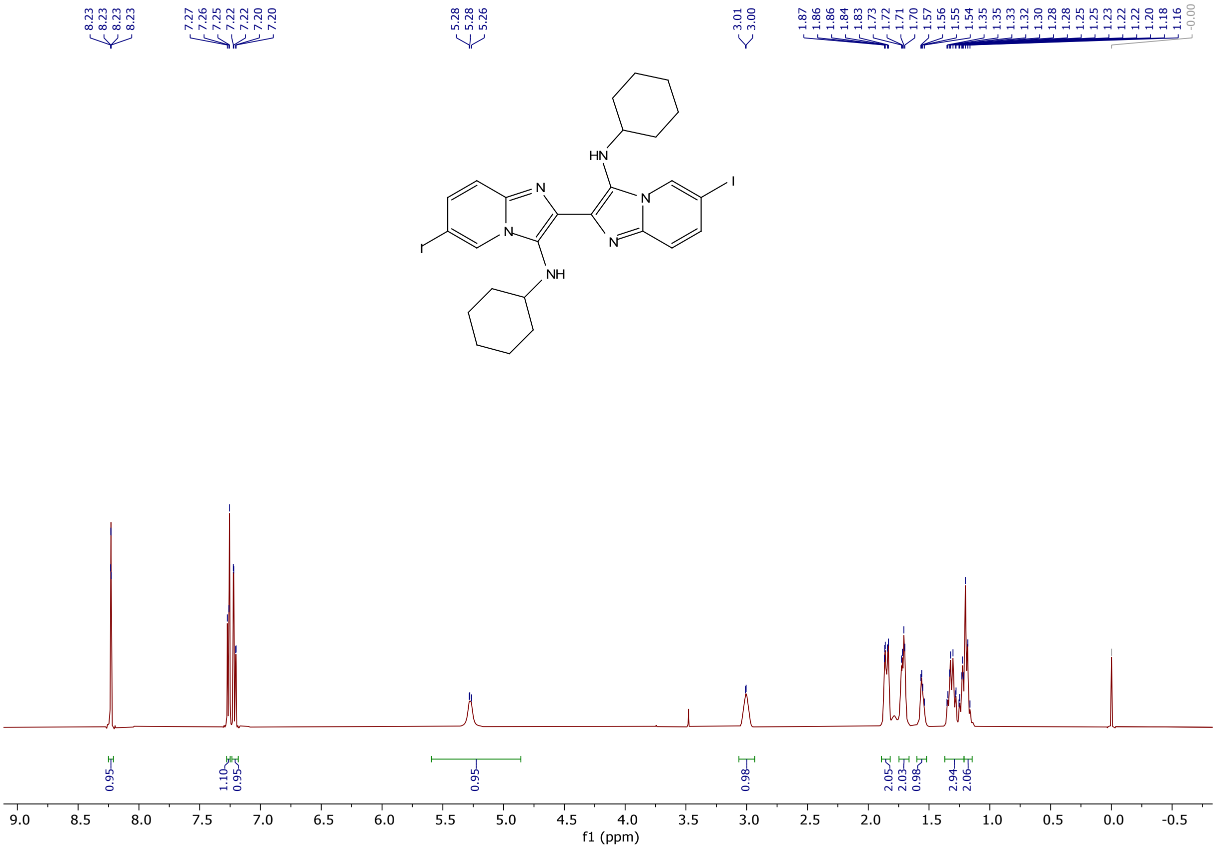

$^{13}C\{^1H\}$ NMR spectrum of **3aj** (126 MHz, $CDCl_3$)

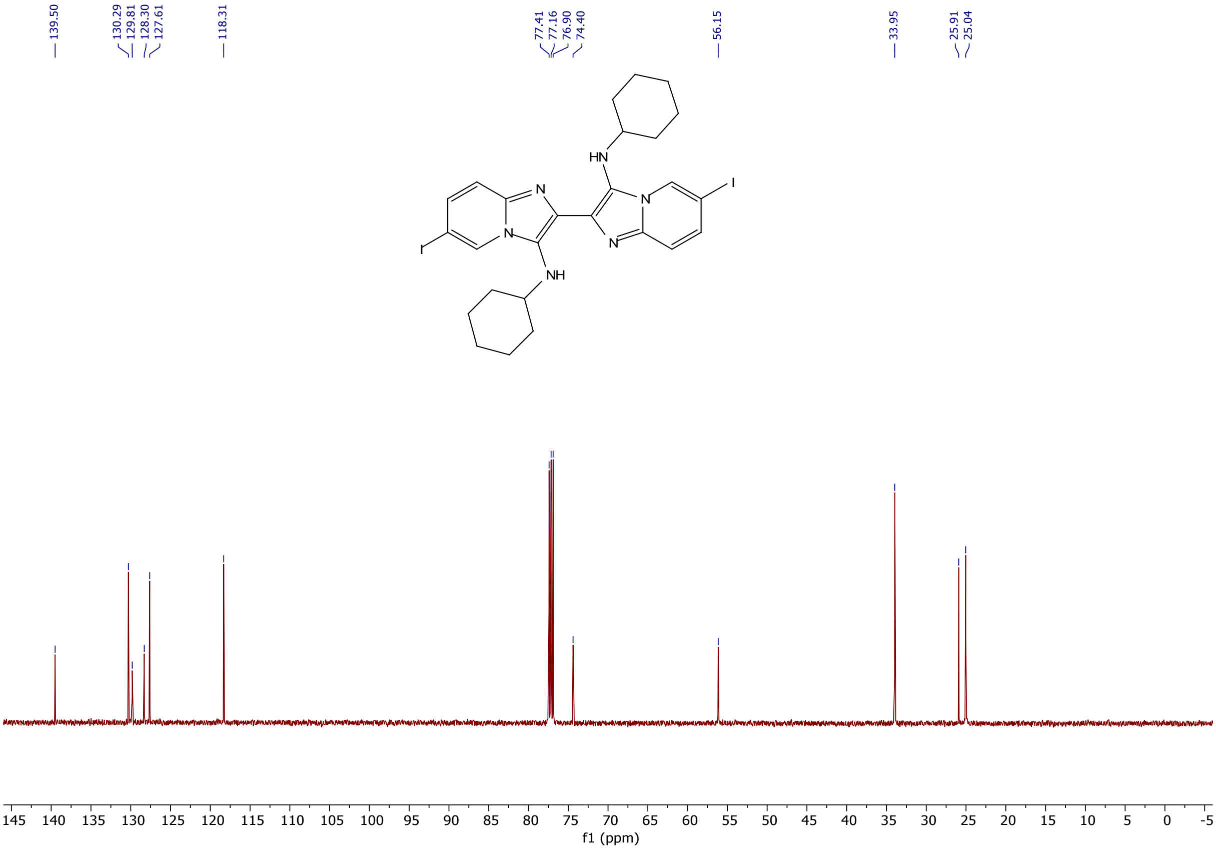

^1H NMR spectrum of **3ak** (500 MHz, $CDCl_3$)

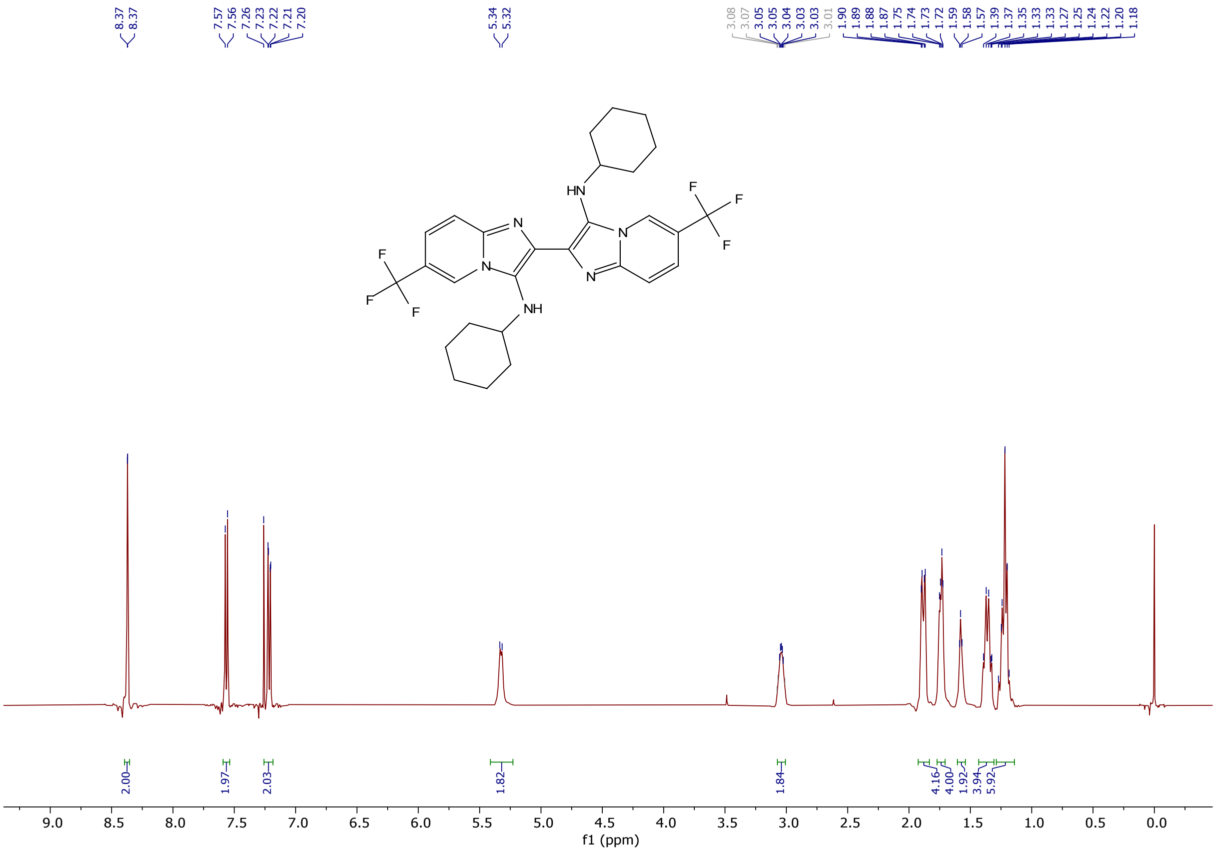

$^{13}C\{^{1}H\}$ NMR spectrum of **3ak** (126 MHz, $CDCl_3$)

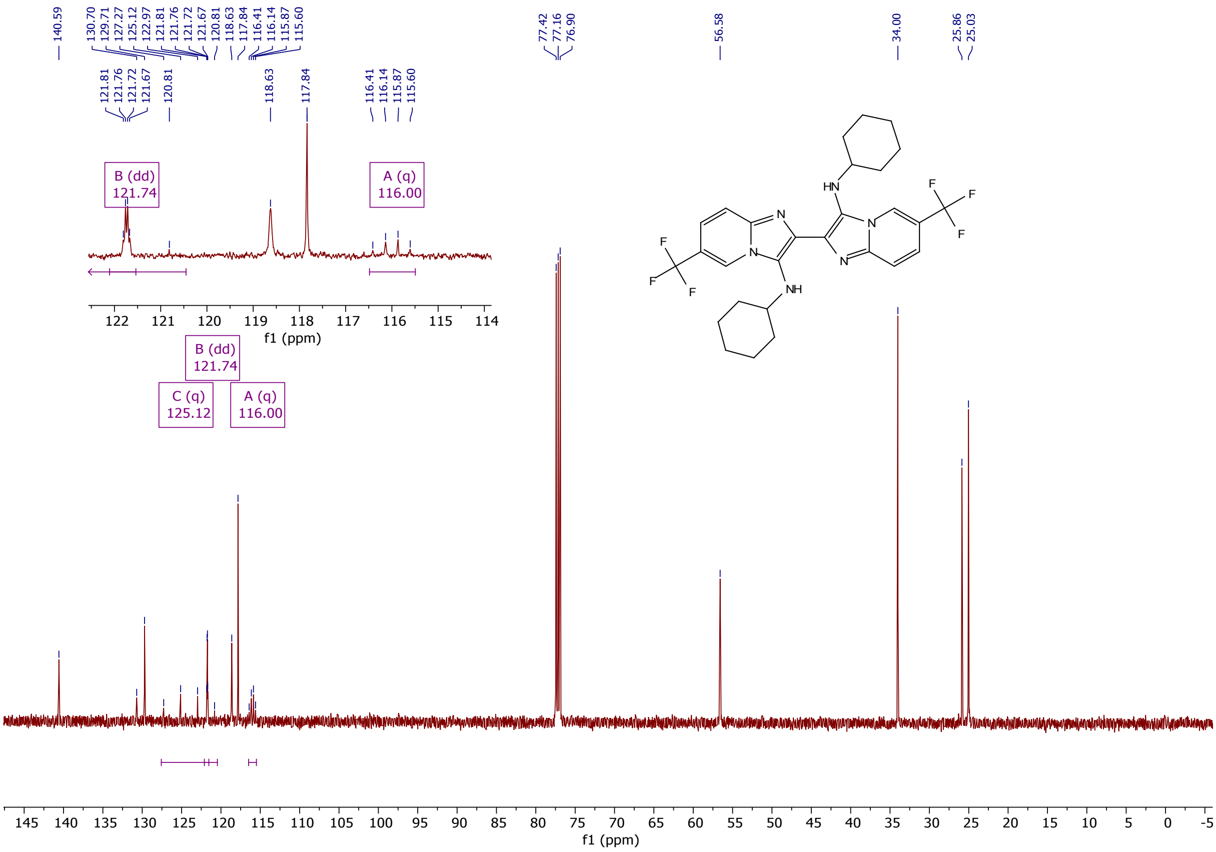

^{1}H NMR spectrum of **3al** (500 MHz, $CDCl_3$)

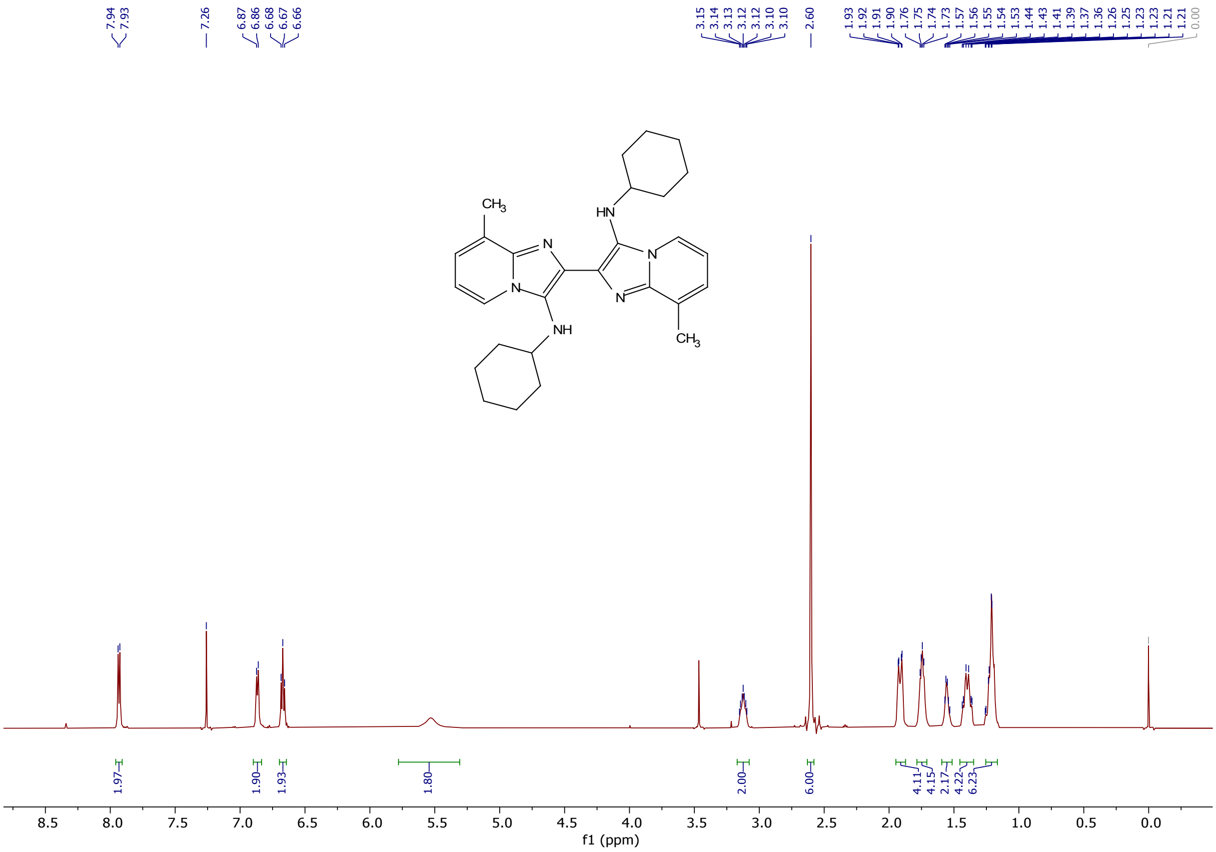

$^{13}C\{^{1}H\}$ NMR spectrum of **3al** (126 MHz, $CDCl_3$)

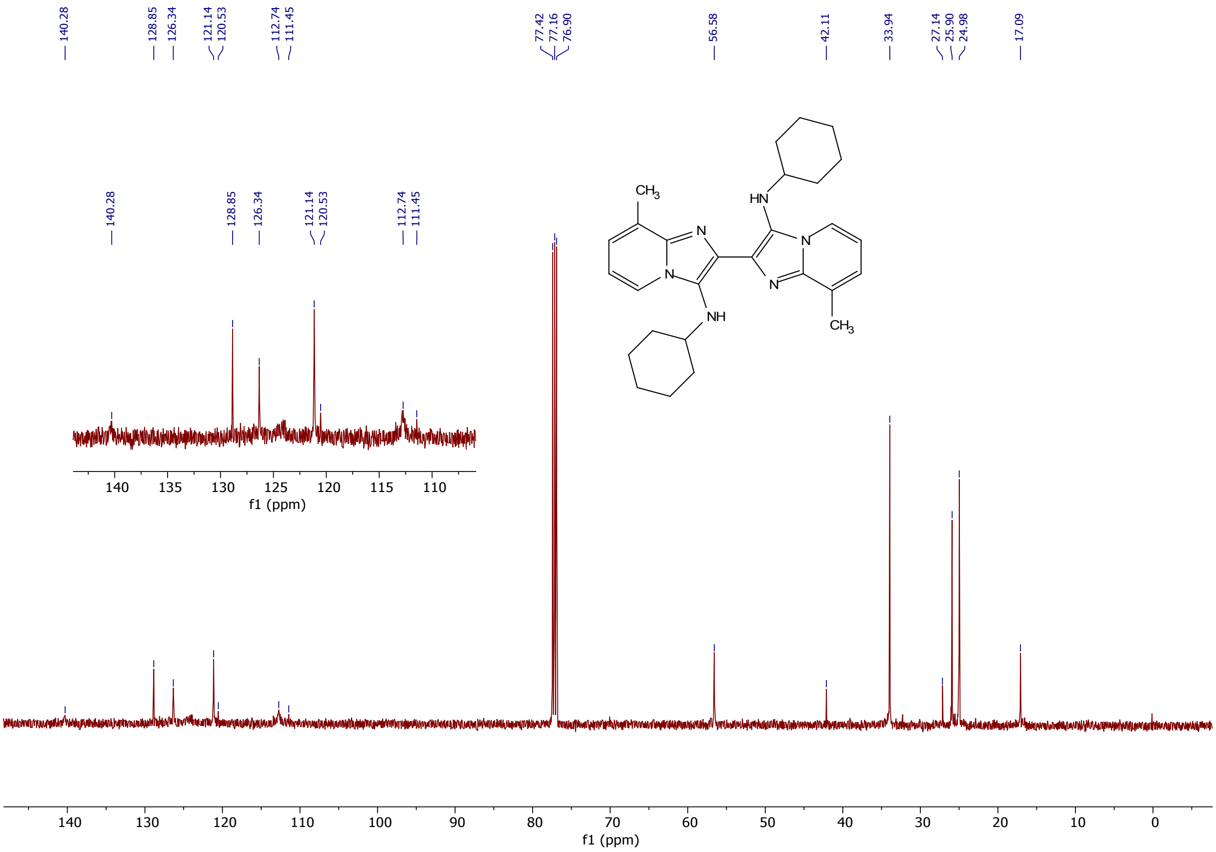

^{1}H NMR spectrum of **3am** (500 MHz, $CDCl_3$)

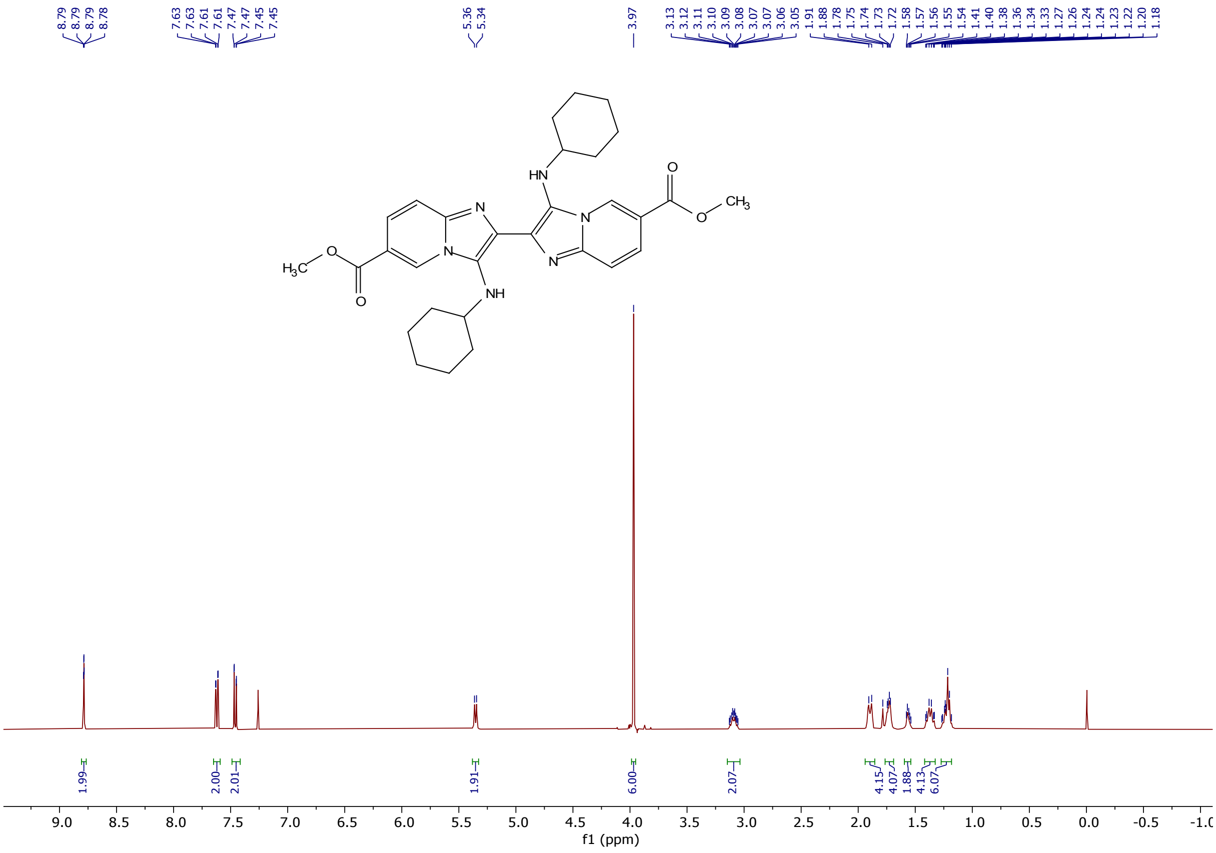

$^{13}C\{^{1}H\}$ NMR spectrum of **3am** (126 MHz, $CDCl_3$)

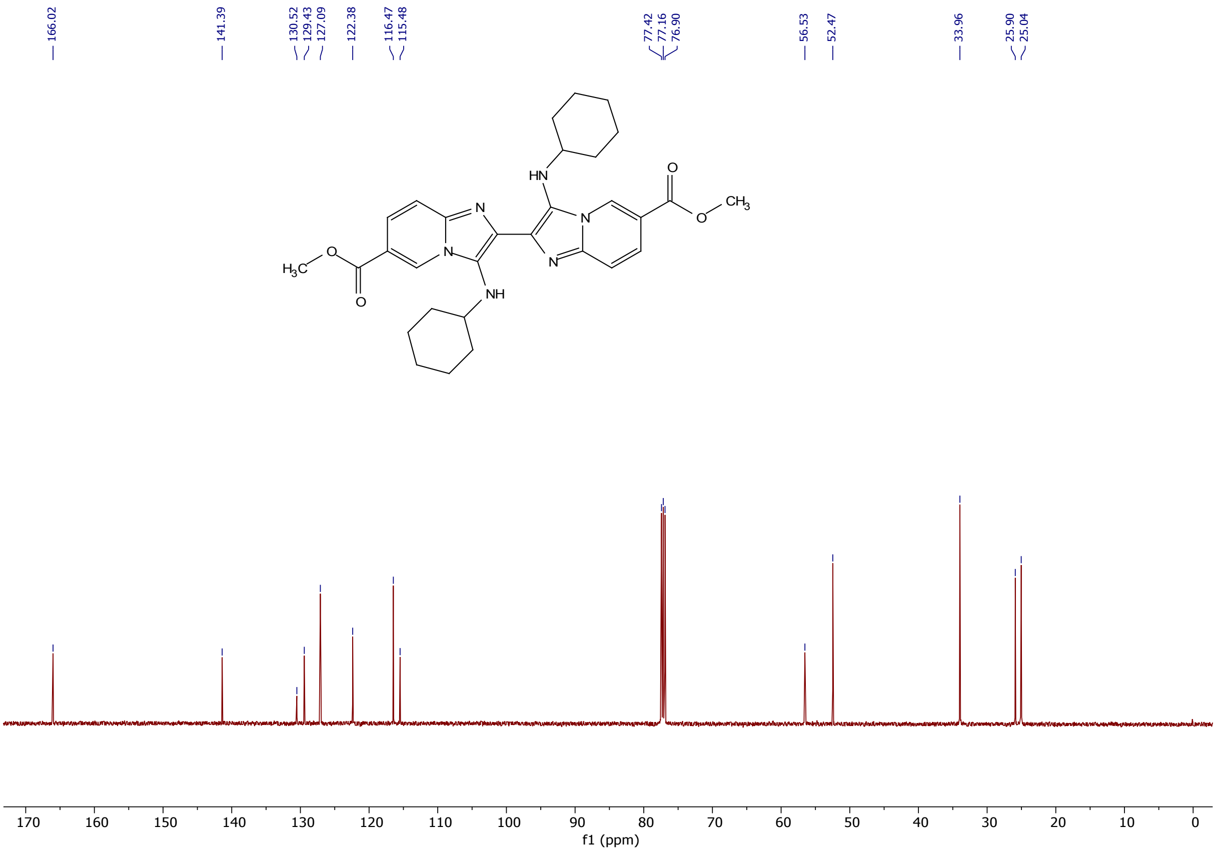

^{1}H NMR spectrum of **3an** (500 MHz, $CDCl_3$)

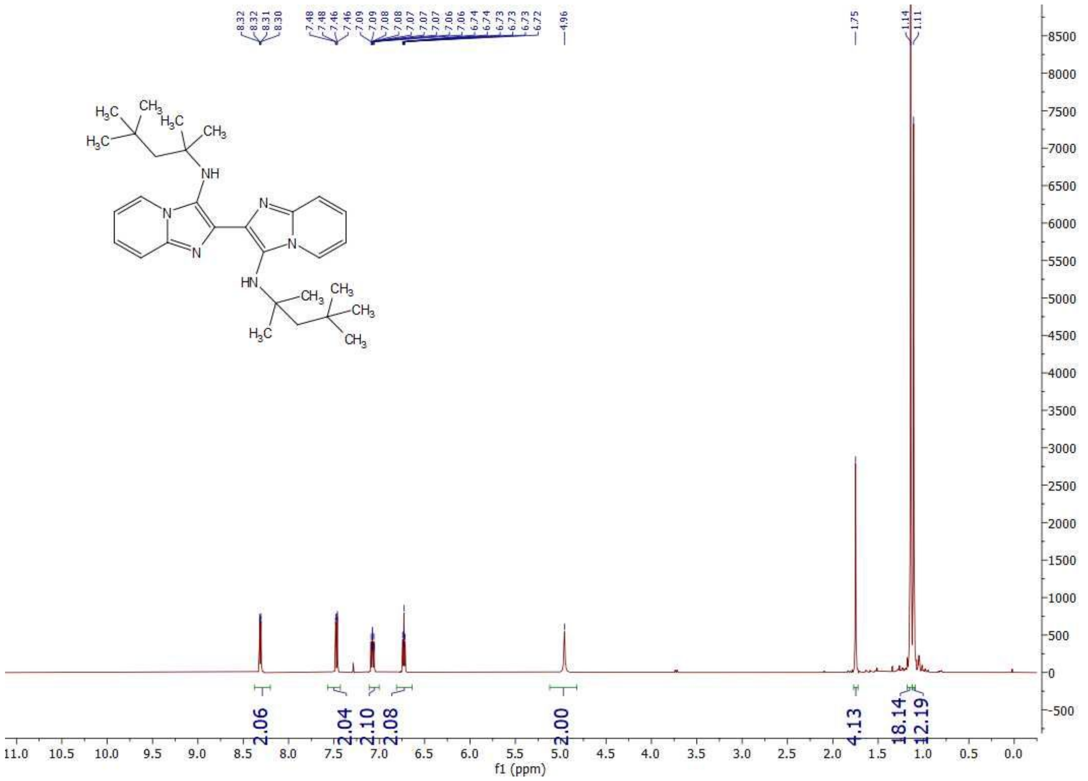

$^{13}C\{^{1}H\}$ NMR spectrum of **3an** (126 MHz, $CDCl_3$)

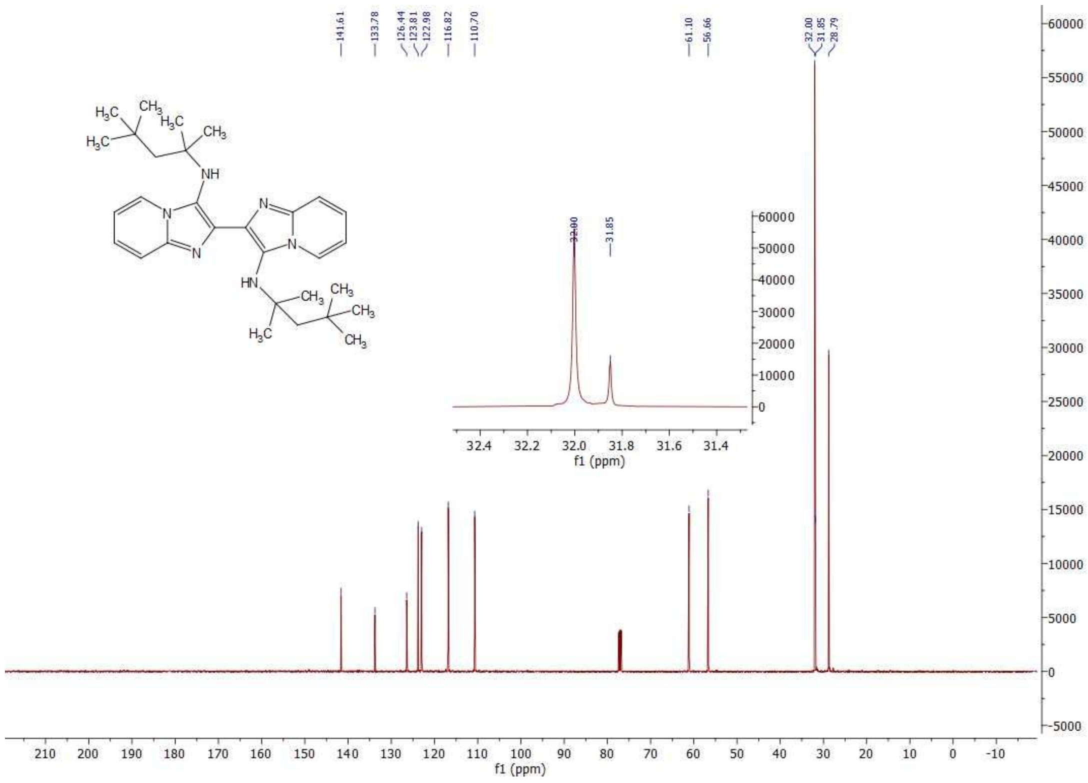

^{1}H NMR spectrum of **3ao** (500 MHz, $CDCl_3$)

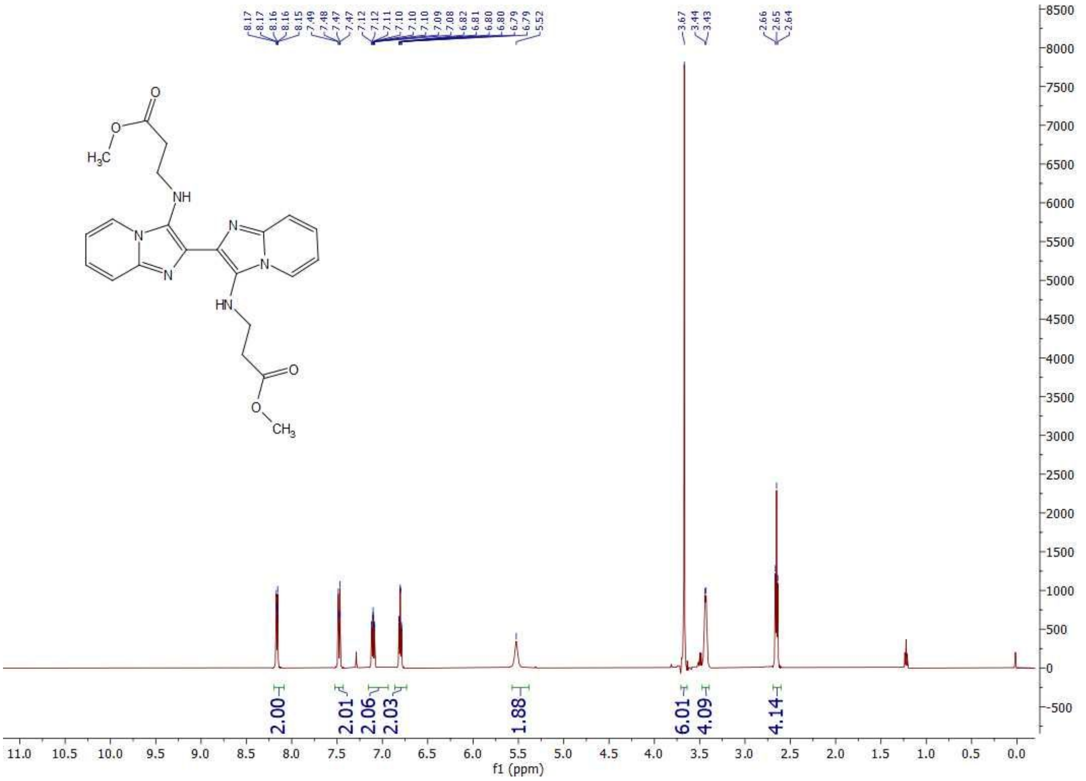

$^{13}C\{^{1}H\}$ NMR spectrum of **3ao** (126 MHz, $CDCl_3$)

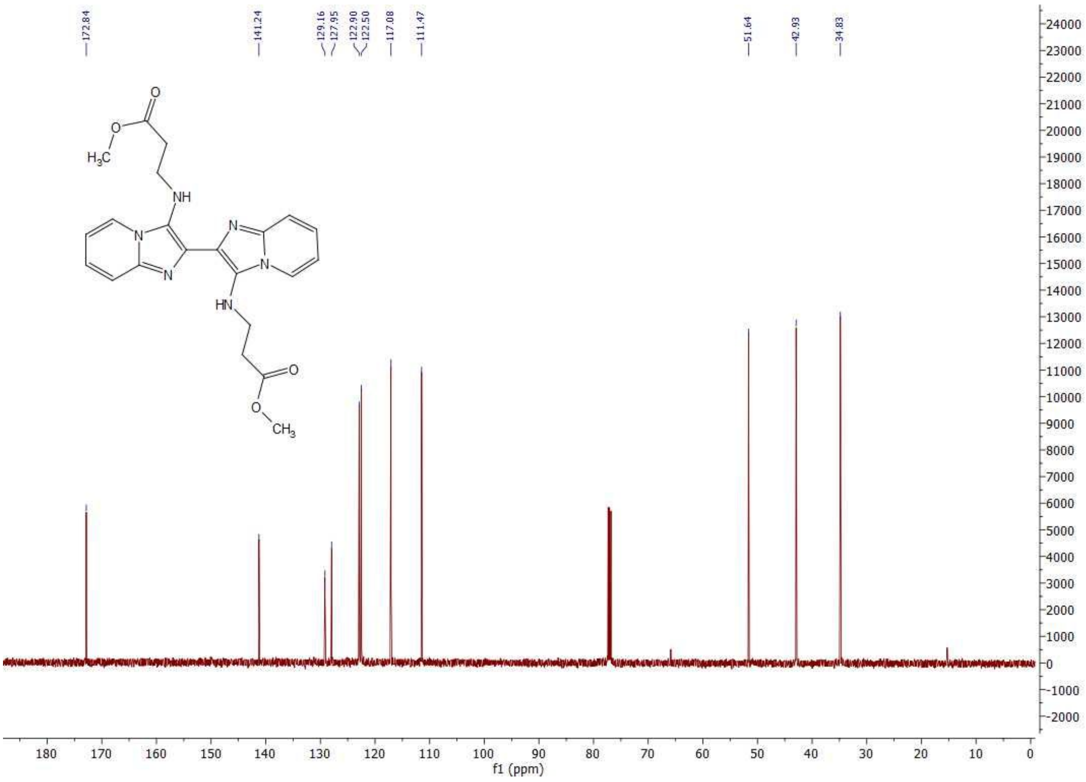

^{1}H NMR spectrum of **3ap** (500 MHz, $CDCl_3$)

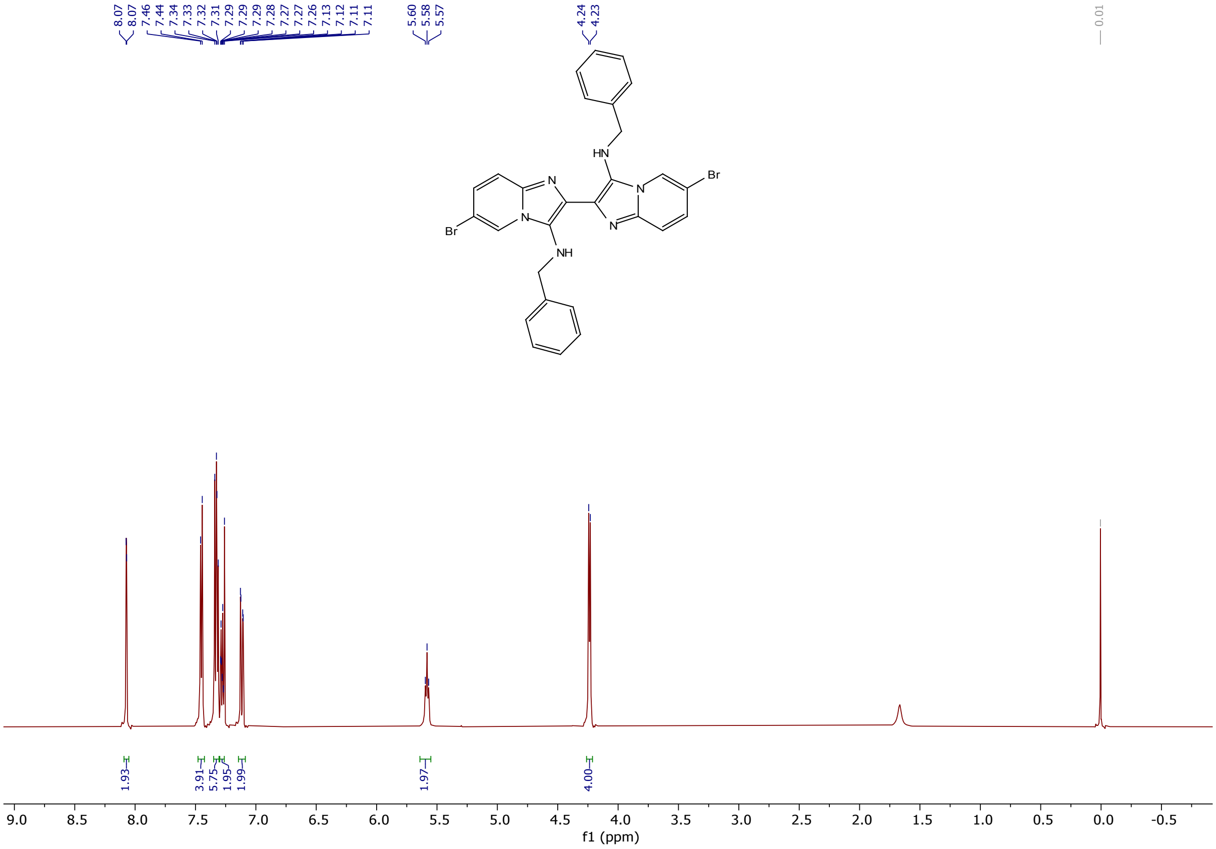

$^{13}C\{^{1}H\}$ NMR spectrum of **3ap** (126 MHz, $CDCl_3$)

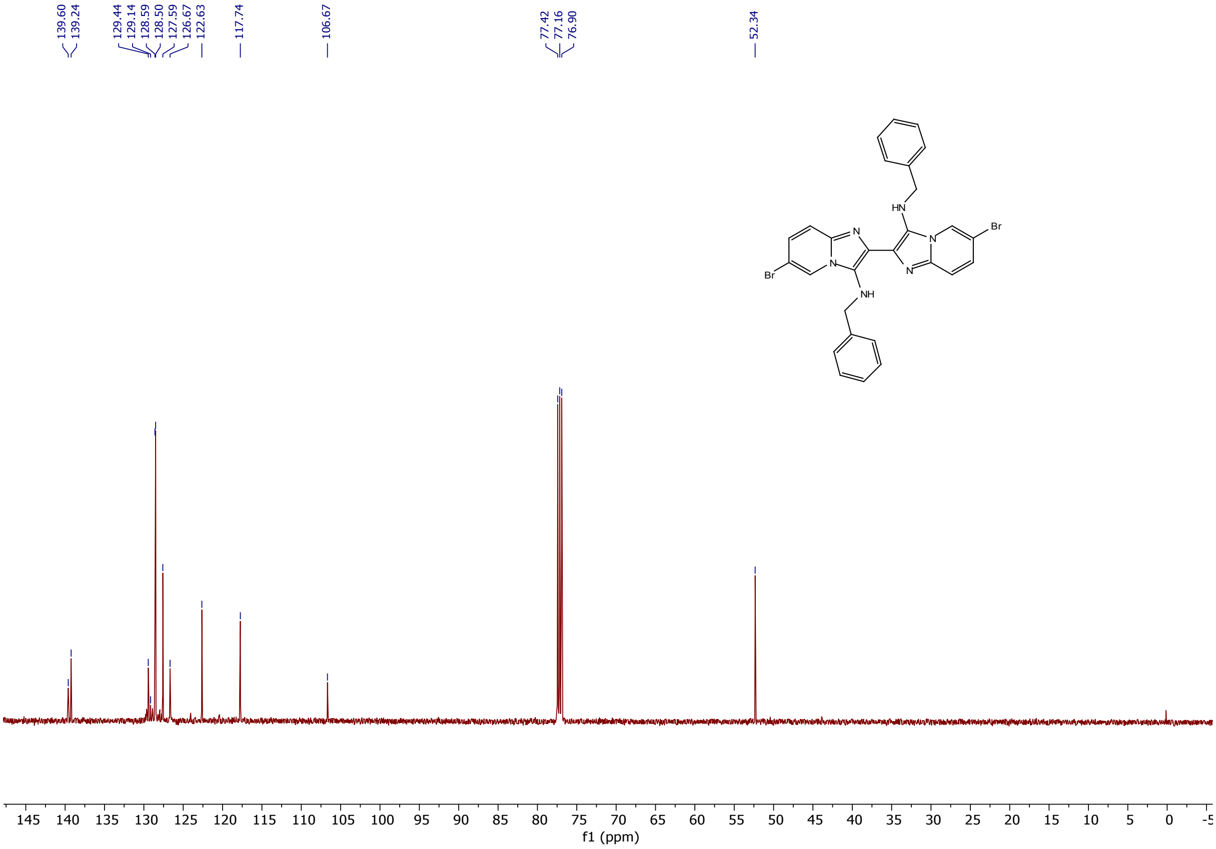

^{1}H NMR spectrum of **3aq** (500 MHz, $CDCl_3$)

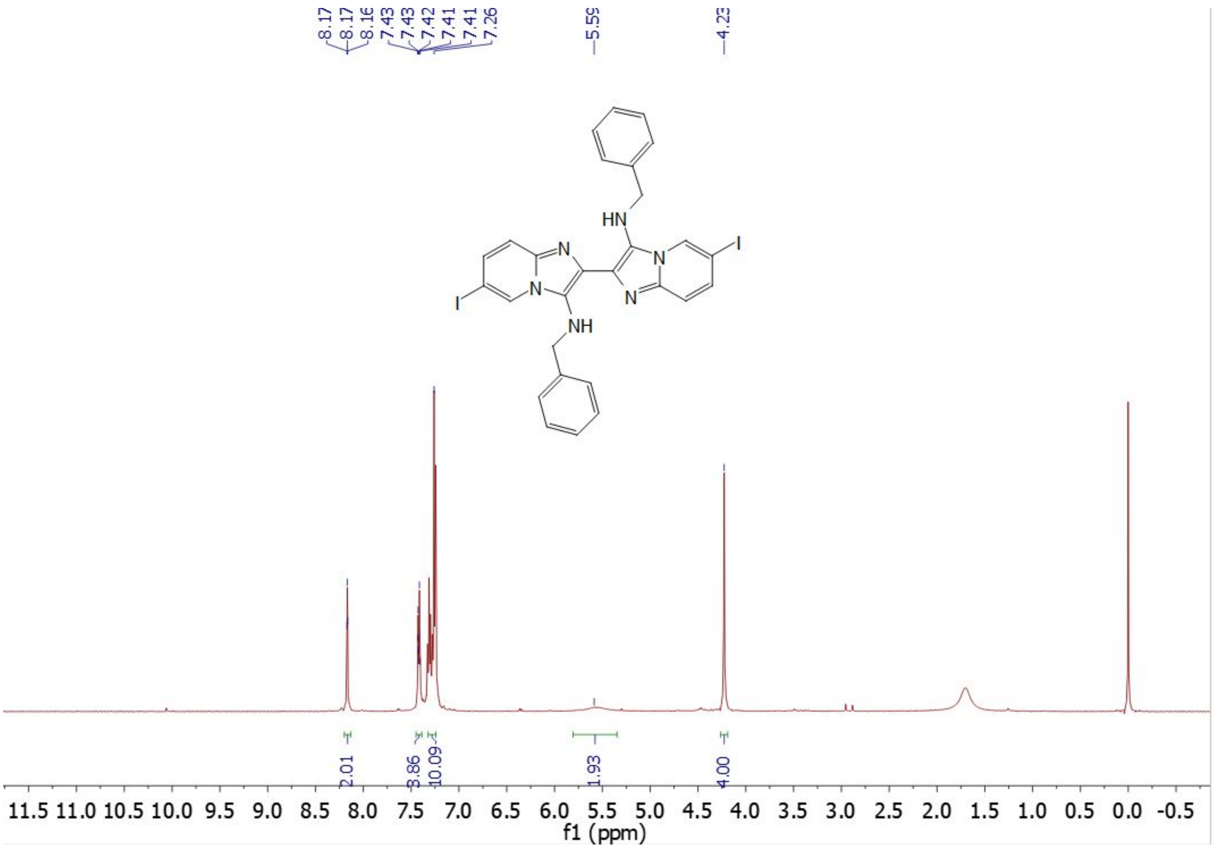

$^{13}C\{^{1}H\}$ NMR spectrum of **3aq** (126 MHz, $CDCl_3$)

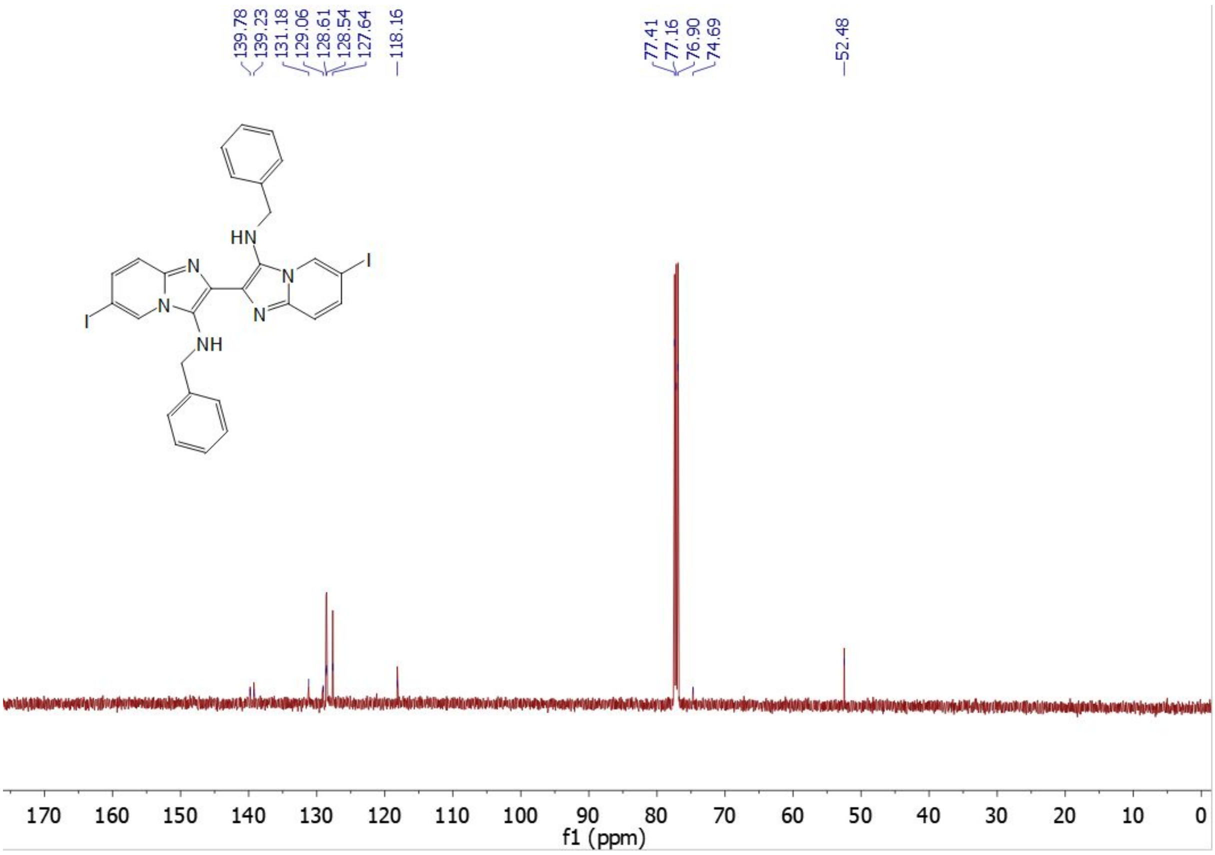

^{1}H NMR spectrum of **3ar** (500 MHz, DMSO-d_6)

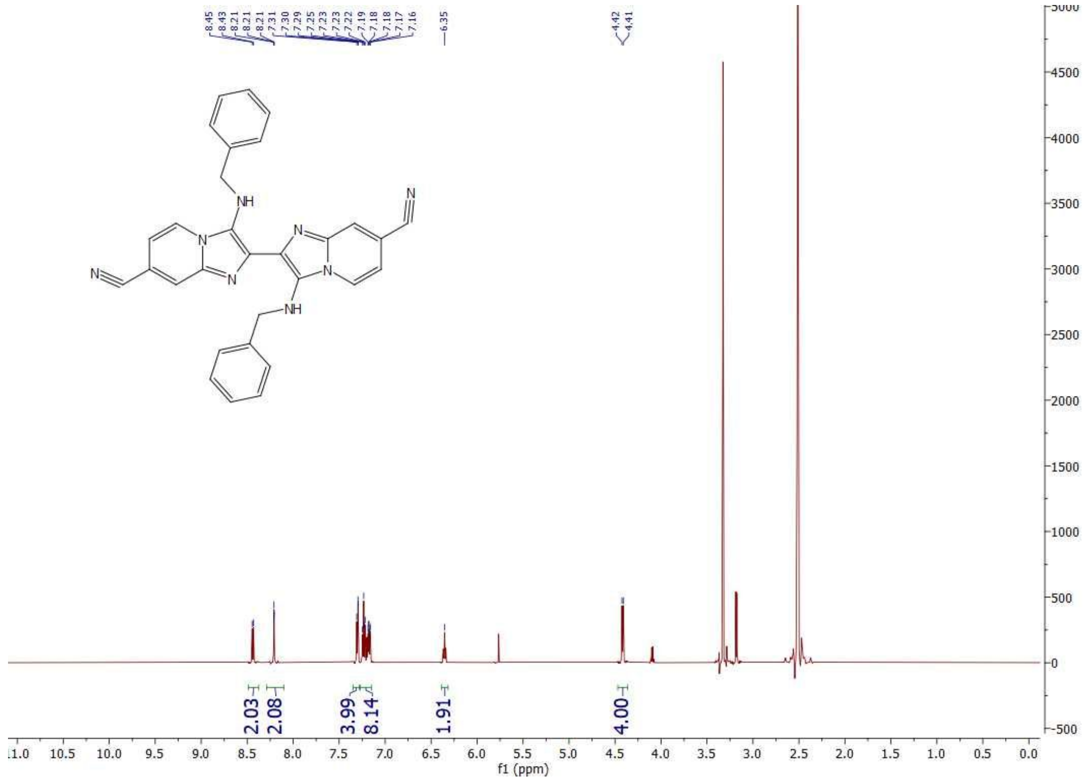

^{1}H NMR spectrum of **3ar** (500 MHz, DMSO-d_6)

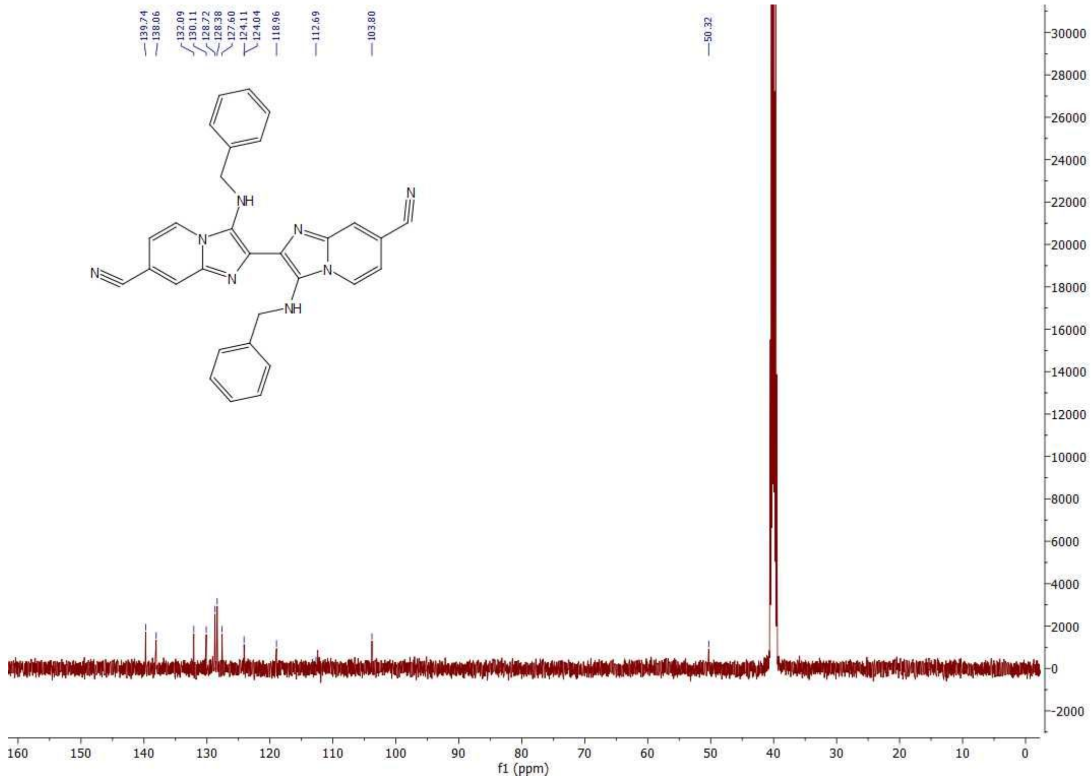

^{1}H NMR spectrum of **3as** (500 MHz, DMSO-d_6)

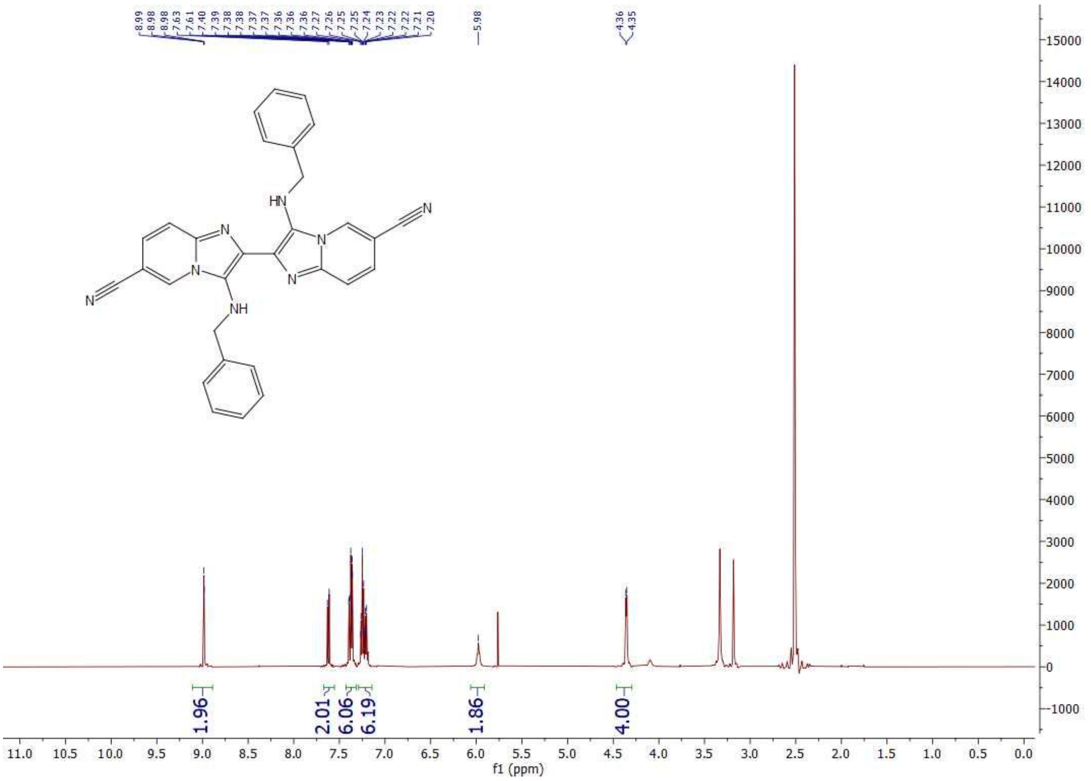

^{1}H NMR spectrum of **3as** (500 MHz, DMSO-d_6)

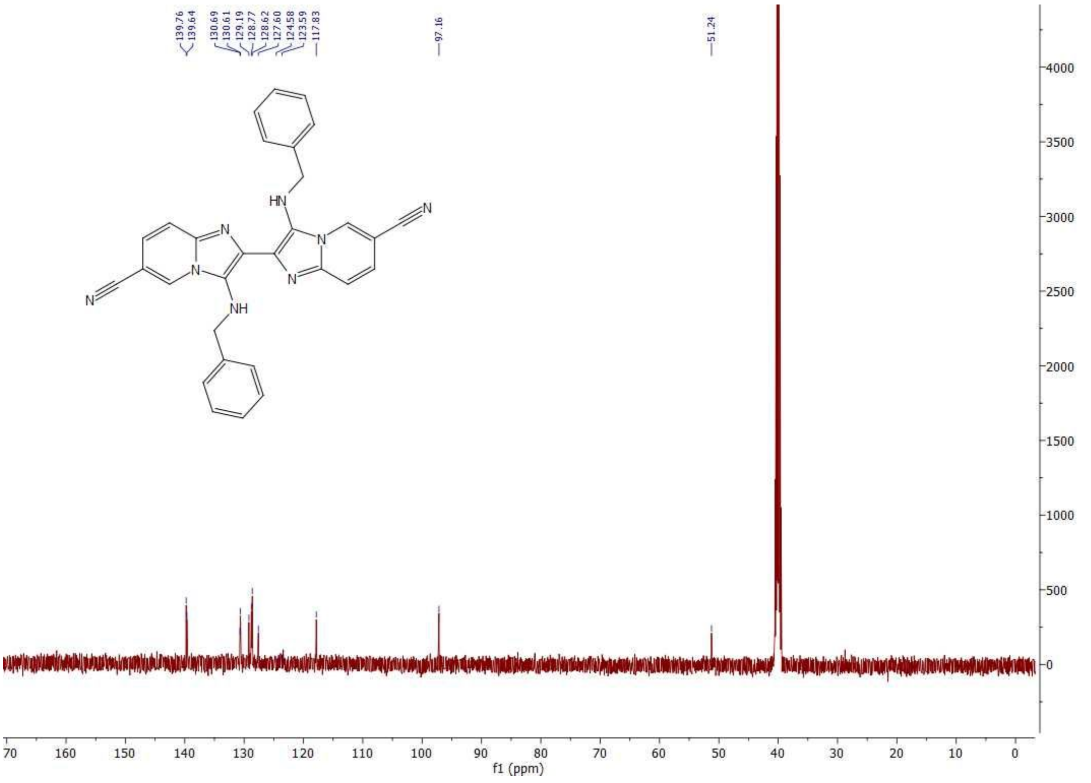

^{1}H NMR spectrum of **3at** (500 MHz, $CDCl_3$)

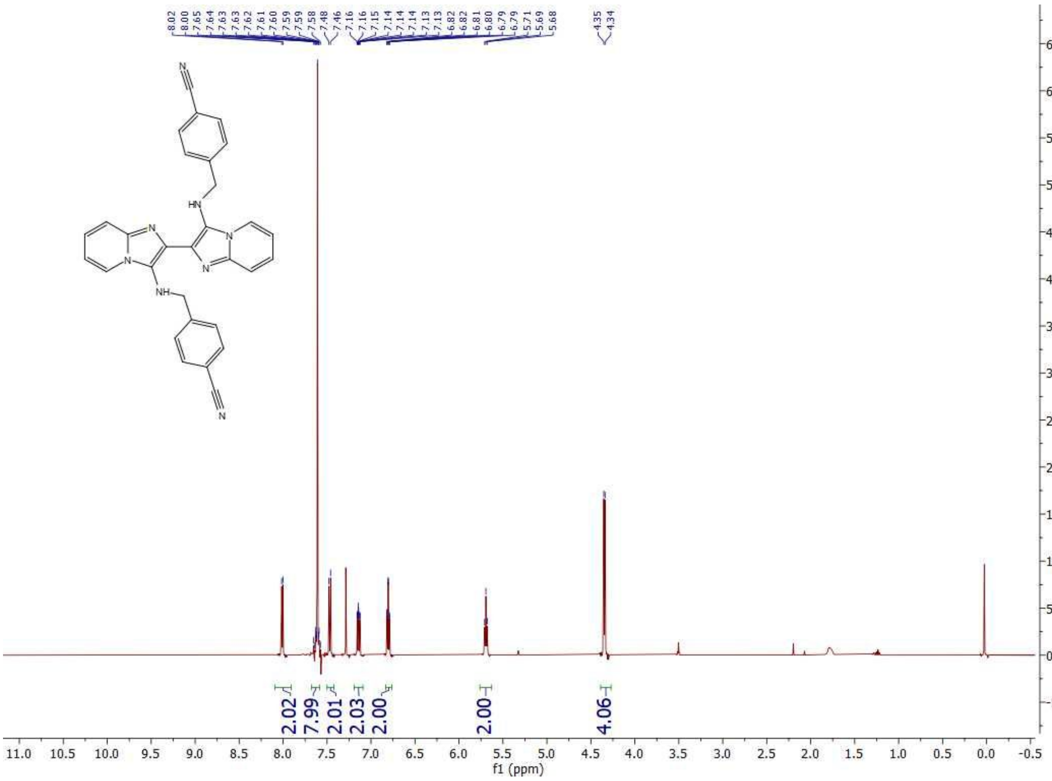

$^{13}C\{^1H\}$ NMR spectrum of **3at** (126 MHz, $CDCl_3$)

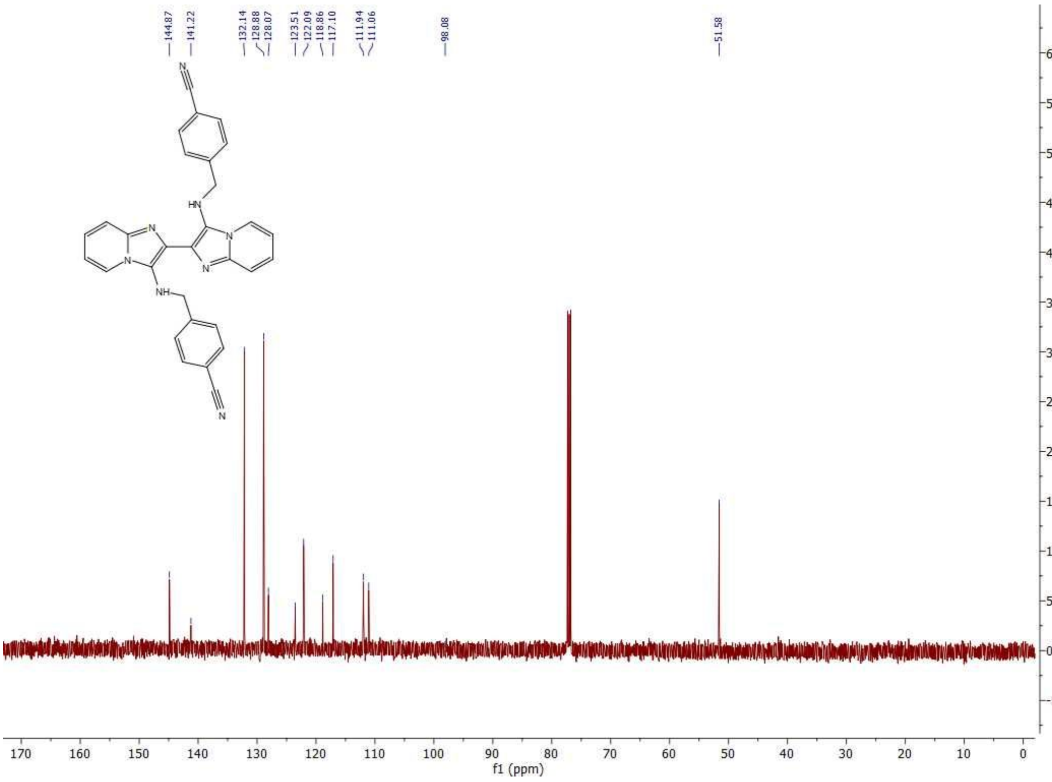

^1H NMR spectrum of **3au** (500 MHz, $CDCl_3$)

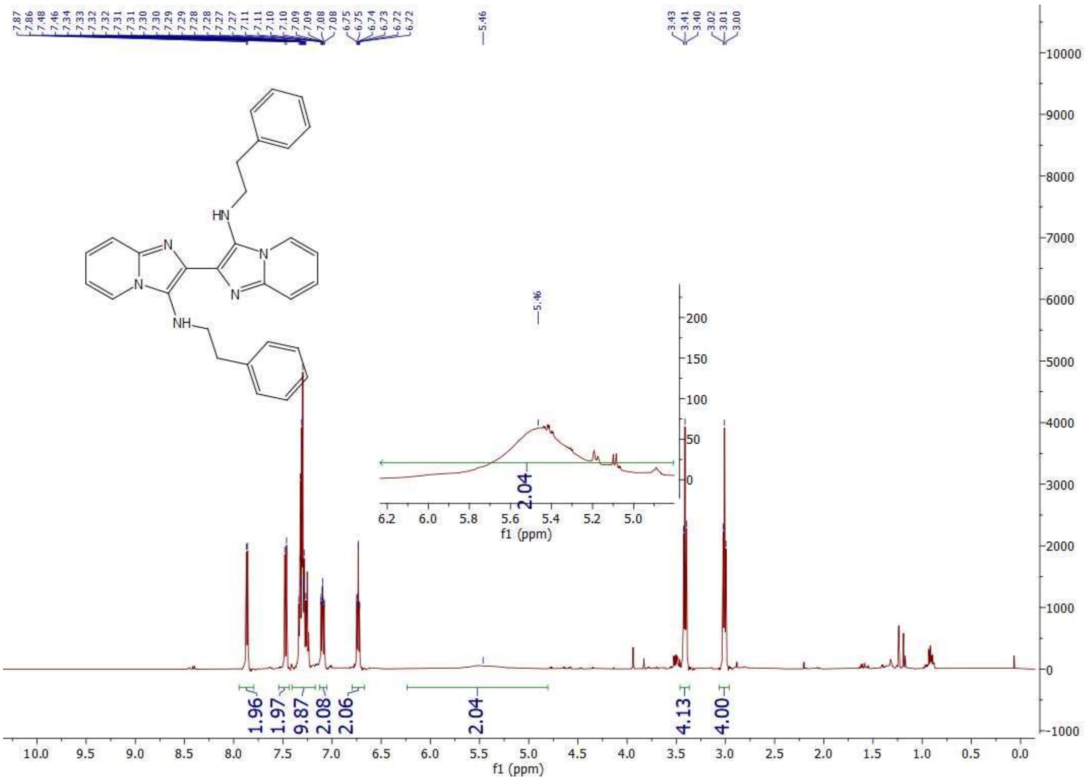

$^{13}C\{^1H\}$ NMR spectrum of **3au** (126 MHz, $CDCl_3$)

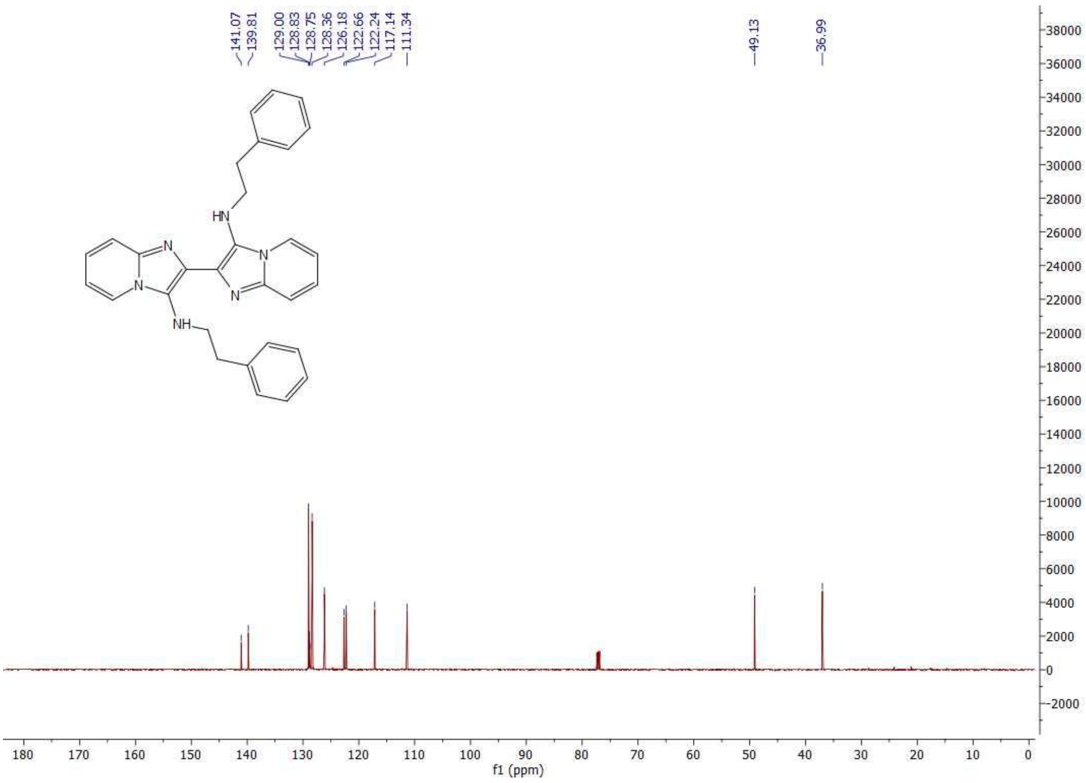

^1H NMR spectrum of **3av** (500 MHz, $CDCl_3$)

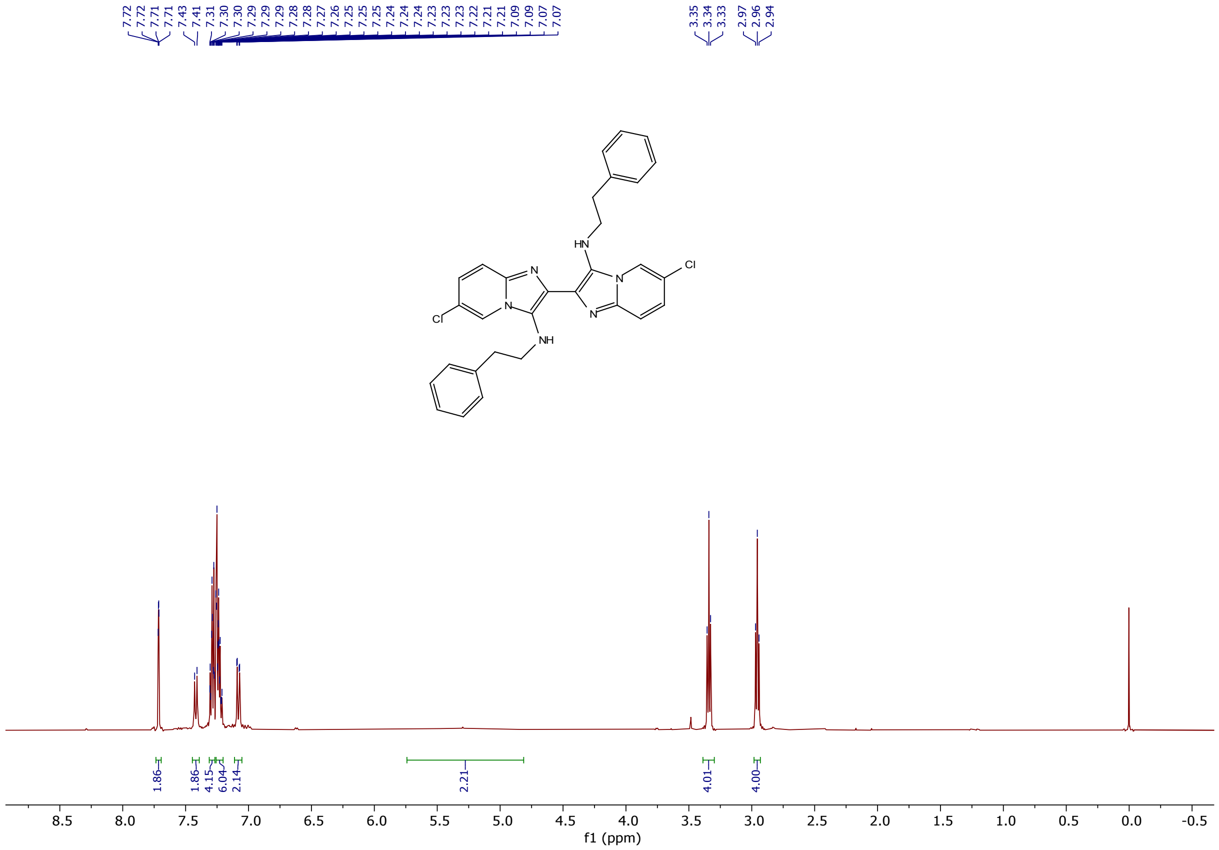

$^{13}C\{^{1}H\}$ NMR spectrum of **3av** (126 MHz, $CDCl_3$)

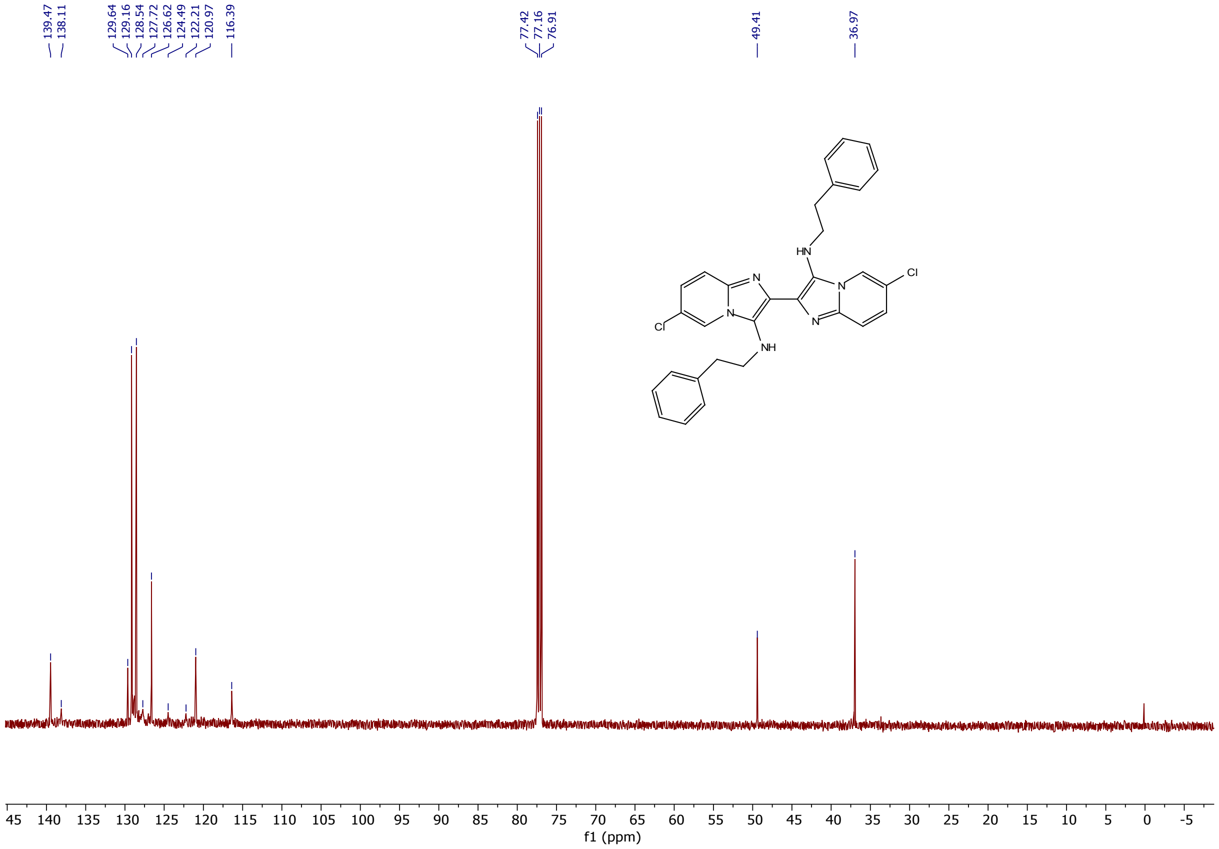

^{1}H NMR spectrum of **3aw** (500 MHz, $CDCl_3$)

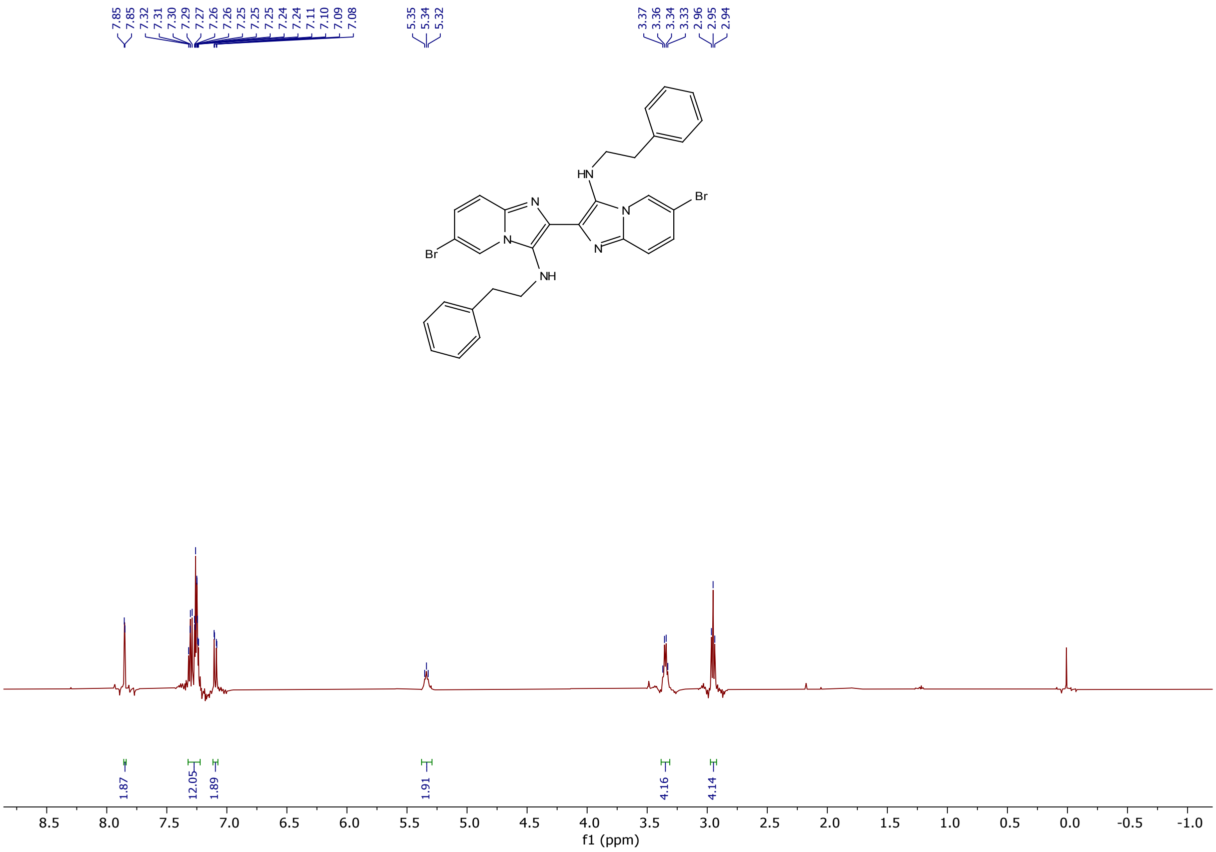

$^{13}C\{^{1}H\}$ NMR spectrum of **3aw** (126 MHz, $CDCl_3$)

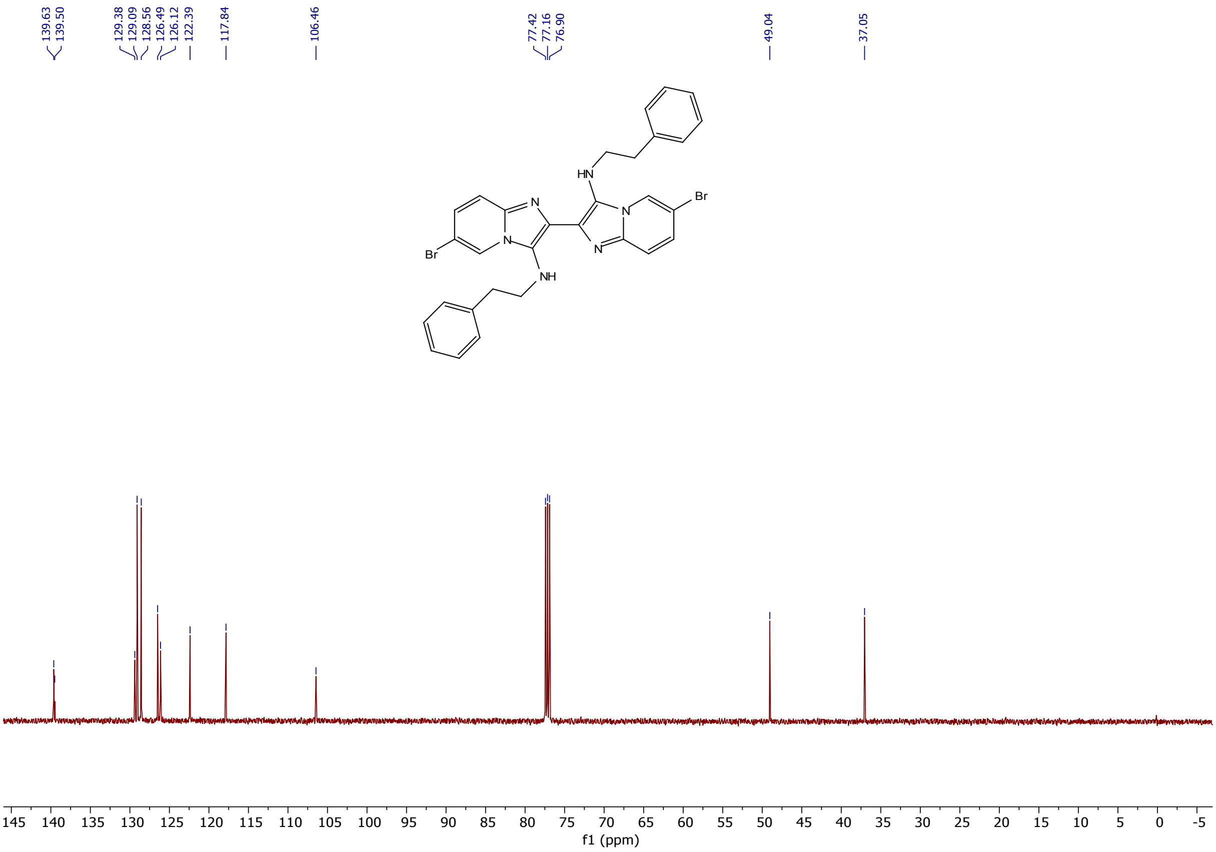

^{1}H NMR spectrum of **3ax** (500 MHz, $CDCl_3$)

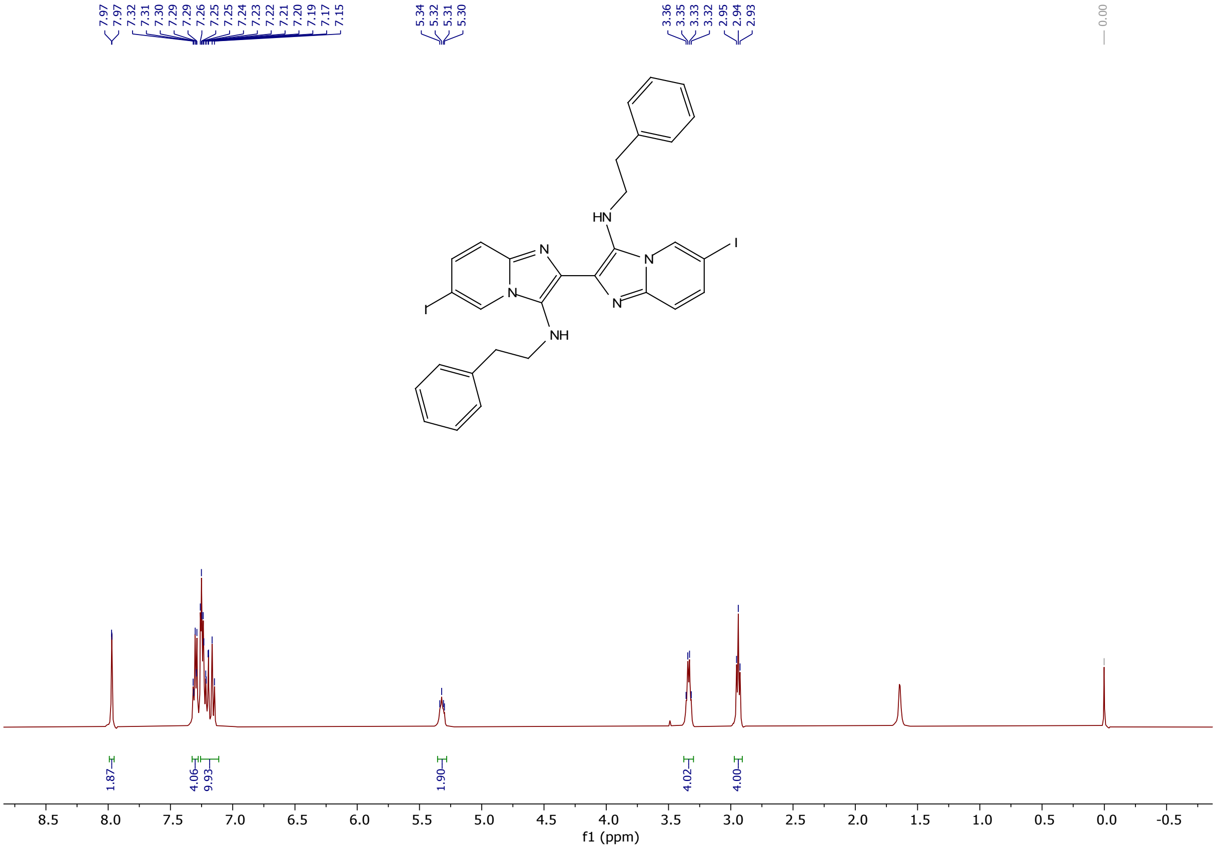

$^{13}C\{^{1}H\}$ NMR spectrum of **3ax** (126 MHz, $CDCl_3$)

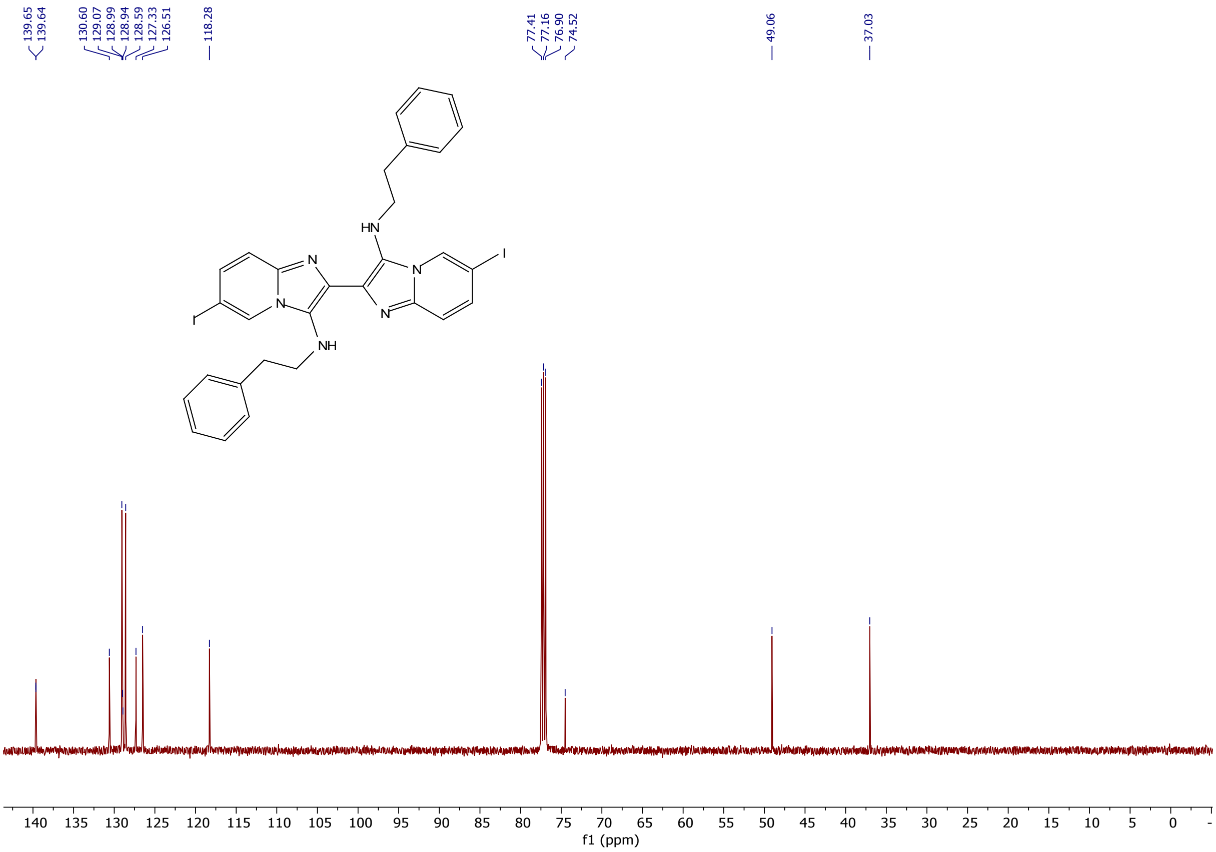

^{1}H NMR spectrum of **3ay** (500 MHz, $CDCl_3$)

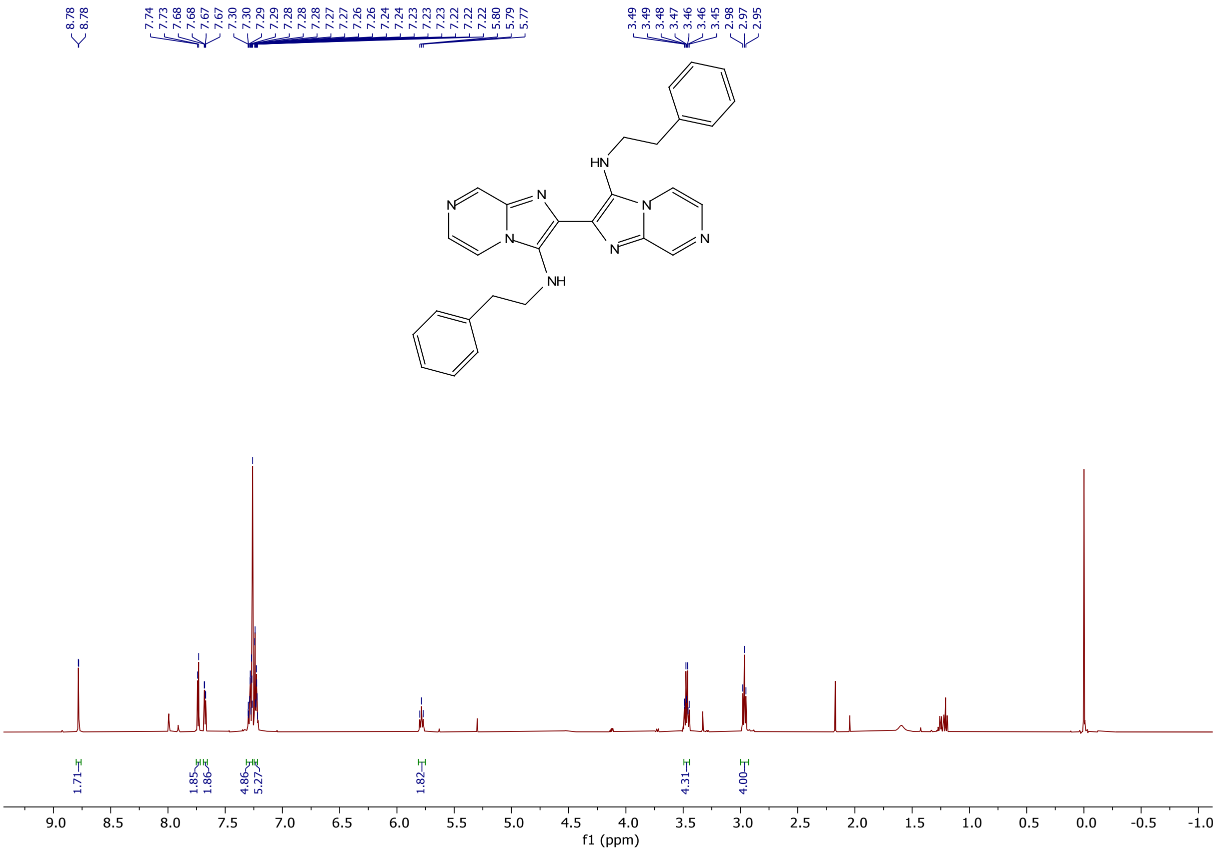

$^{13}C\{^{1}H\}$ NMR spectrum of **3ay** (126 MHz, $CDCl_3$)

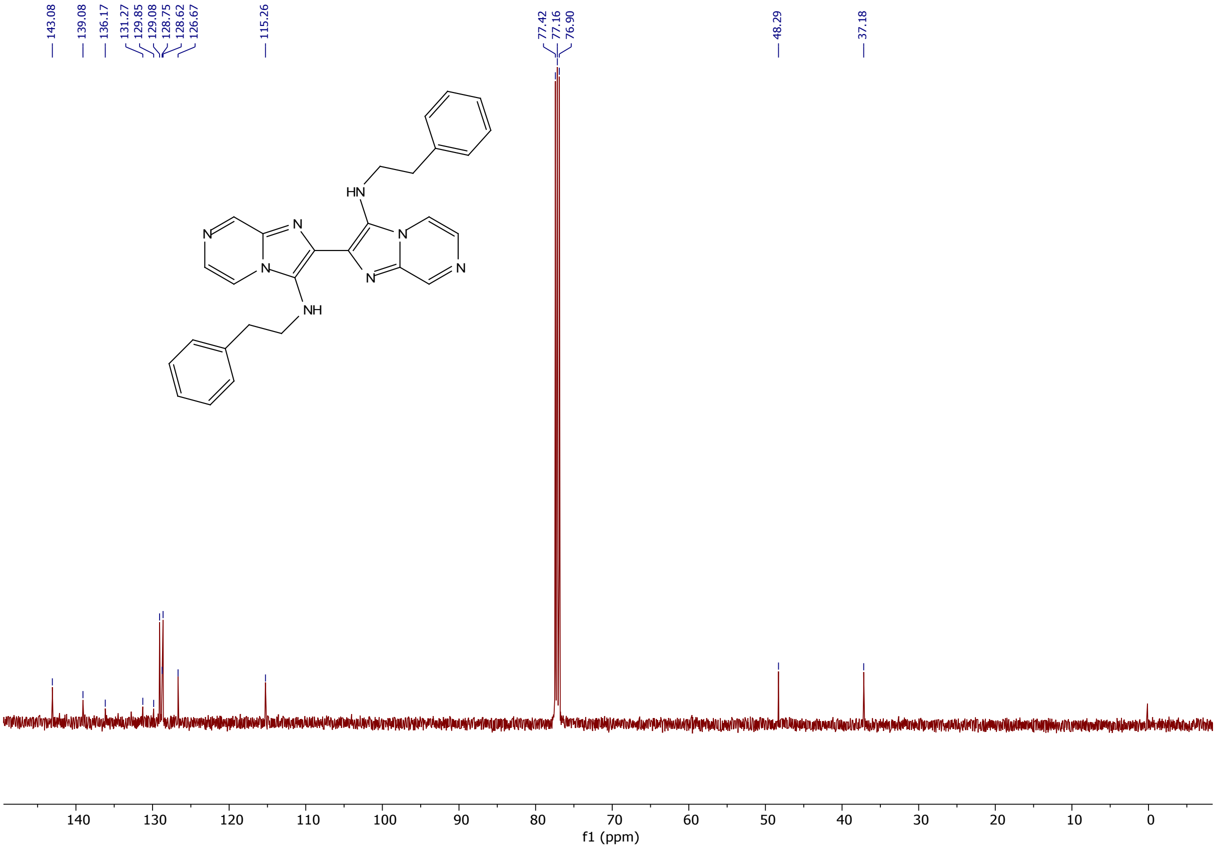

^{1}H NMR spectrum of **3ba** (500 MHz, $CDCl_3$)

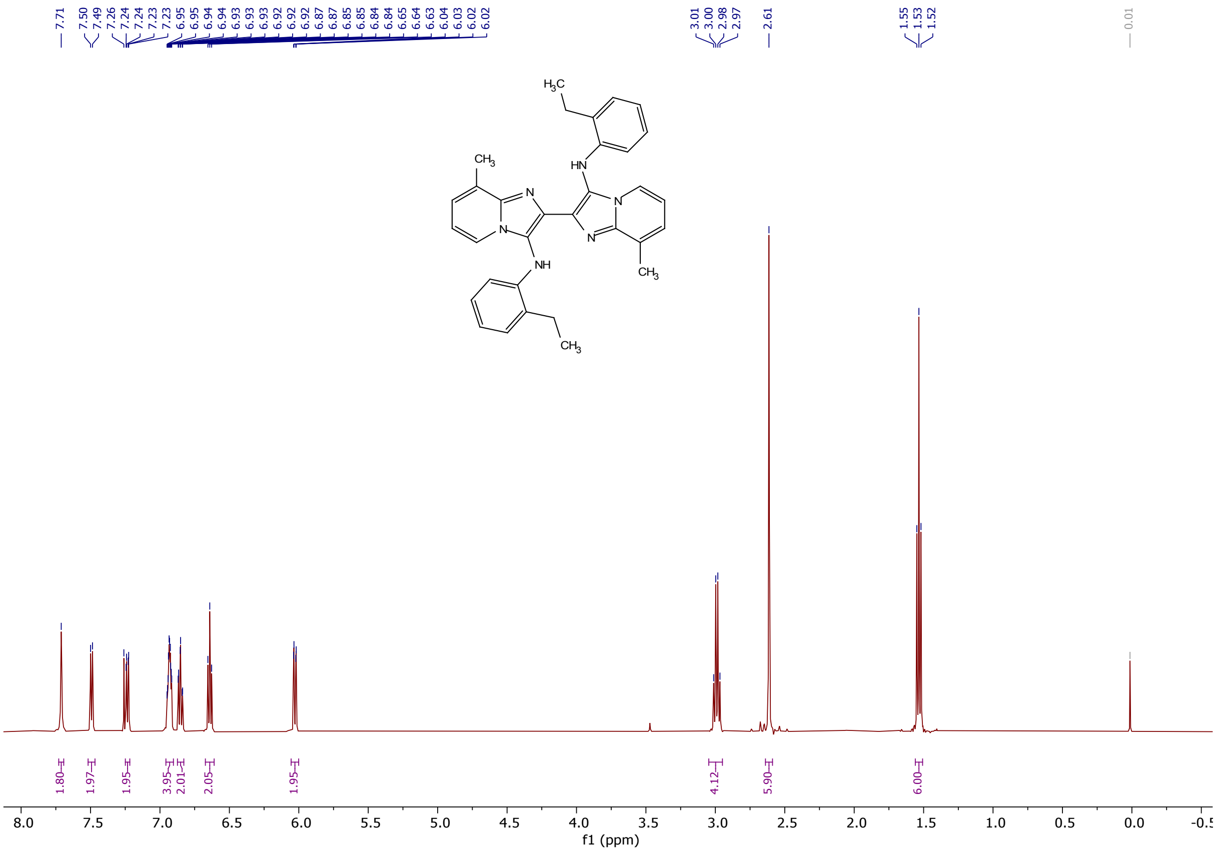

$^{13}C\{^1H\}$ NMR spectrum of **3ba** (126 MHz, $CDCl_3$)

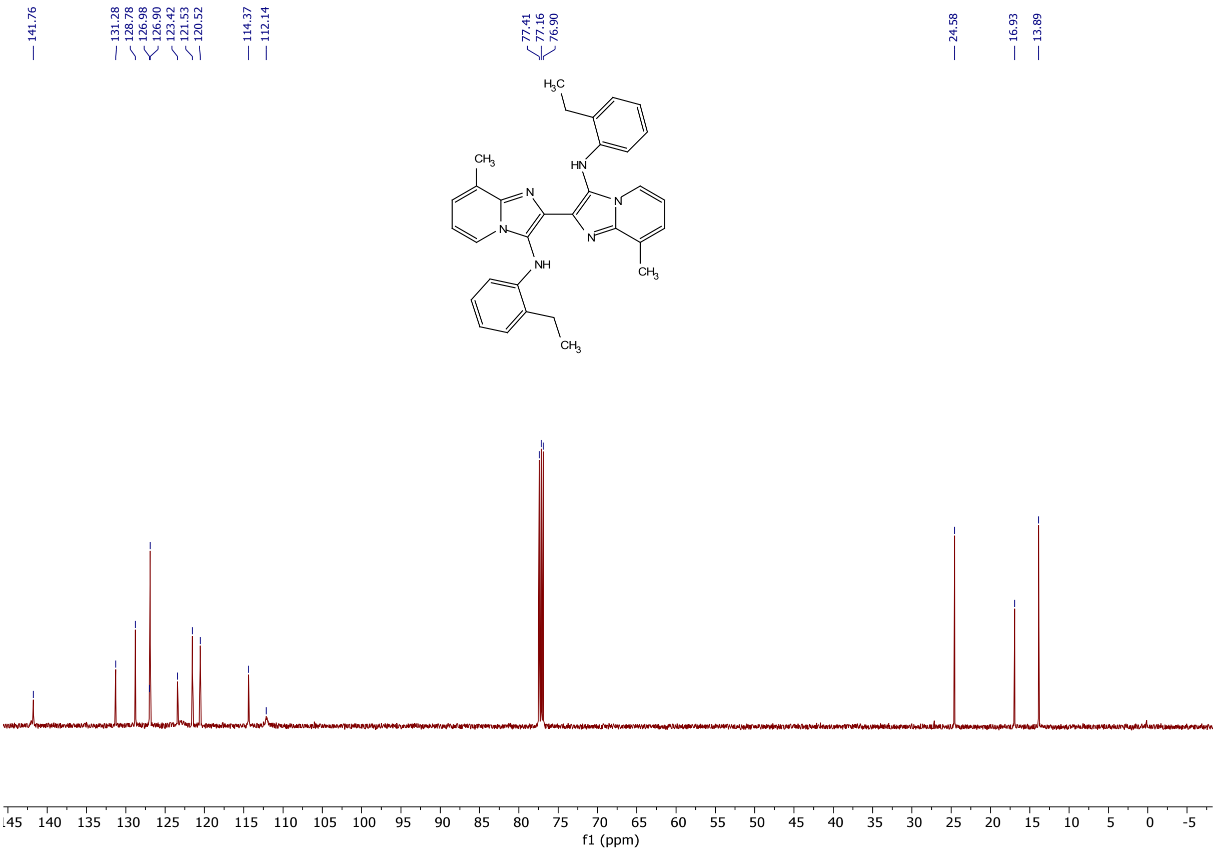

^1H NMR spectrum of **3bb** (500 MHz, $CDCl_3$)

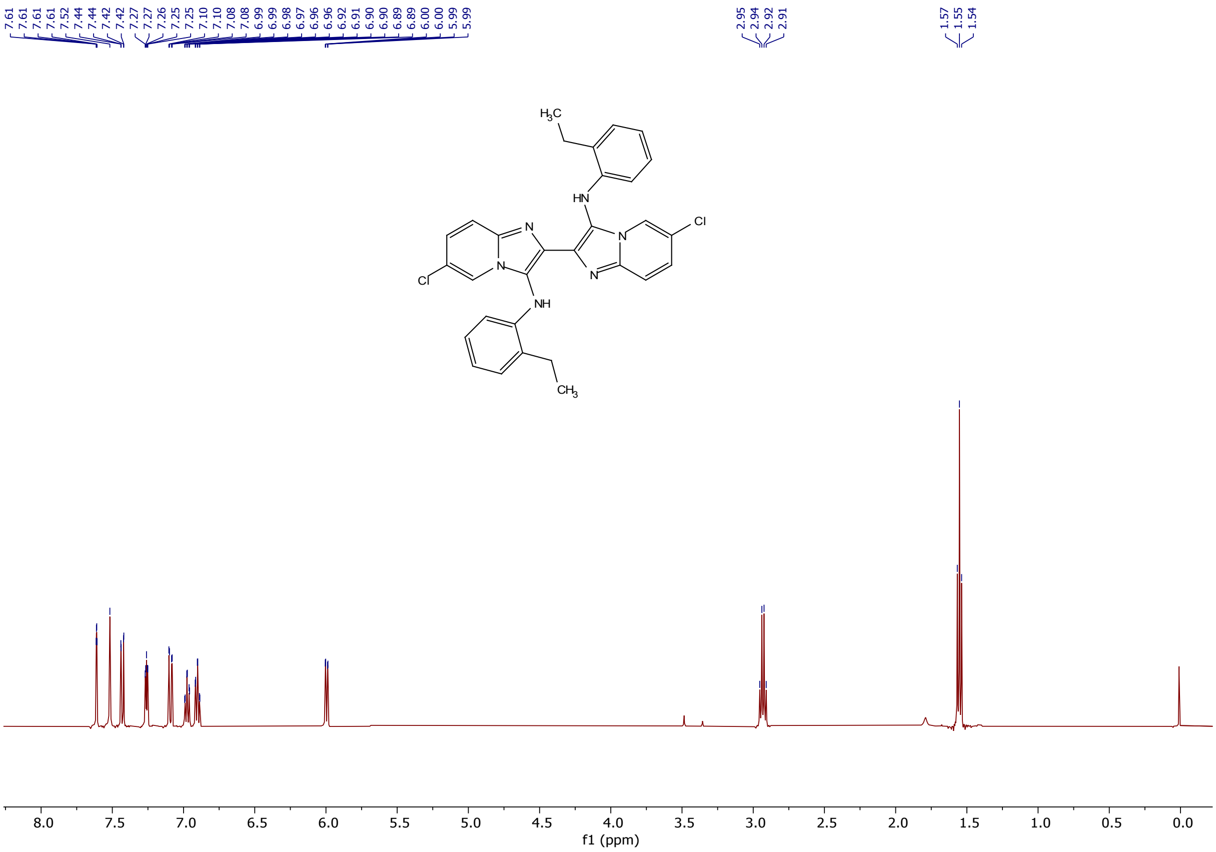

$^{13}C\{^{1}H\}$ NMR spectrum of **3bb** (126 MHz, $CDCl_3$)

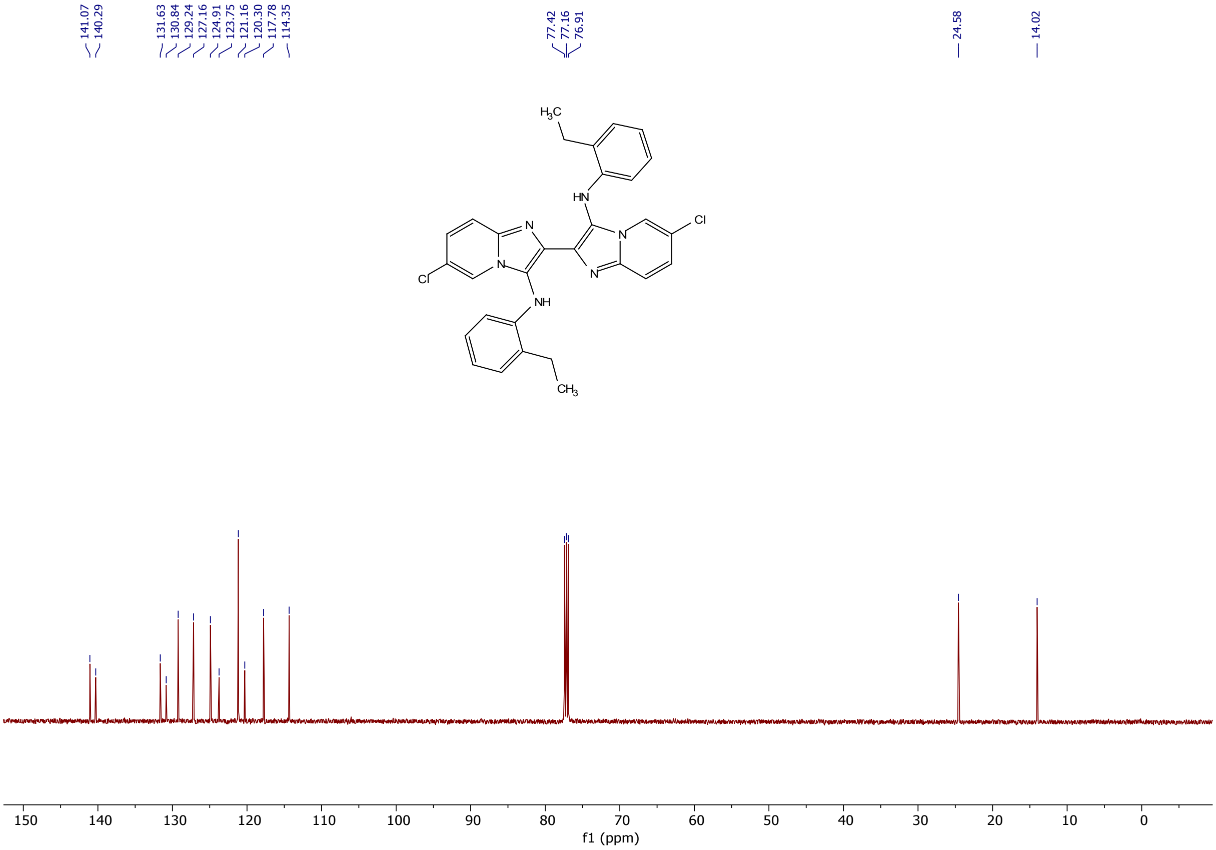

^{1}H NMR spectrum of **3bc** (500 MHz, $CDCl_3$)

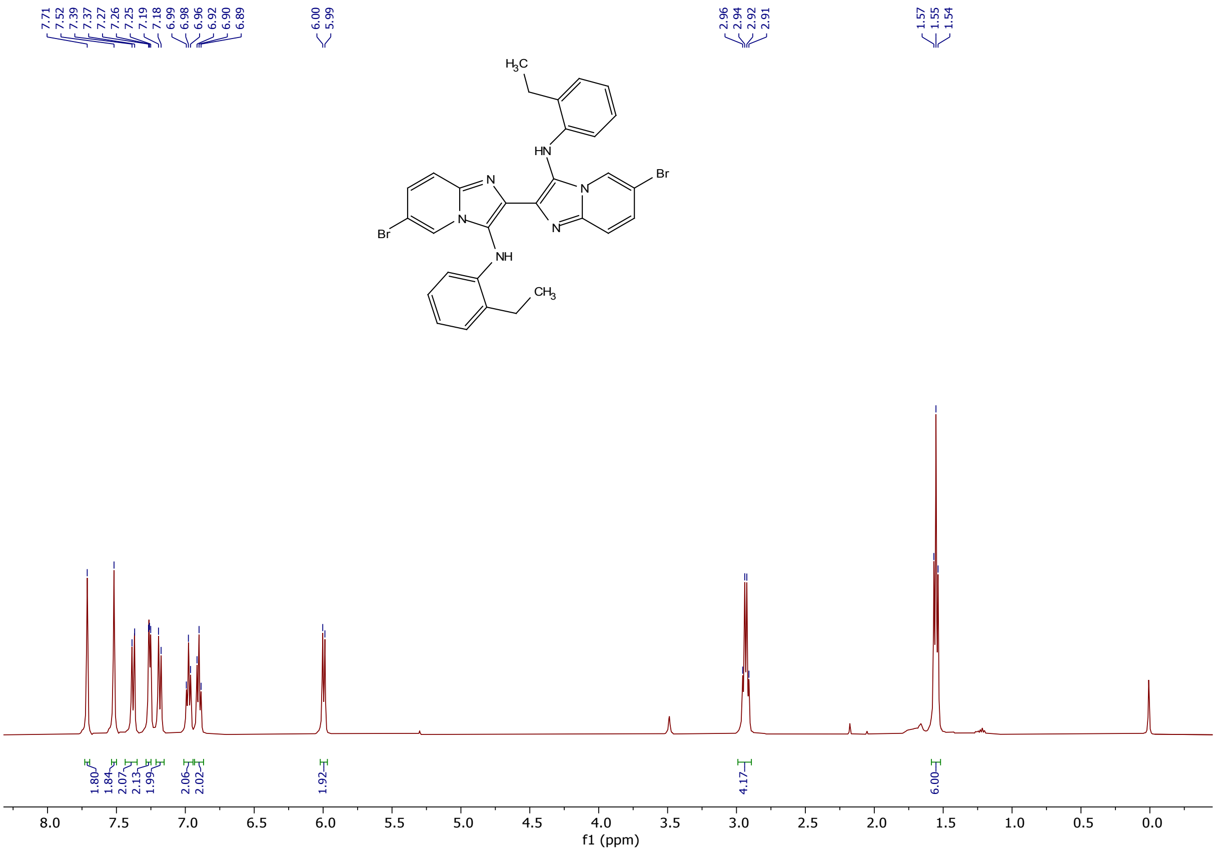

$^{13}C\{^{1}H\}$ NMR spectrum of **3bc** (126 MHz, $CDCl_3$)

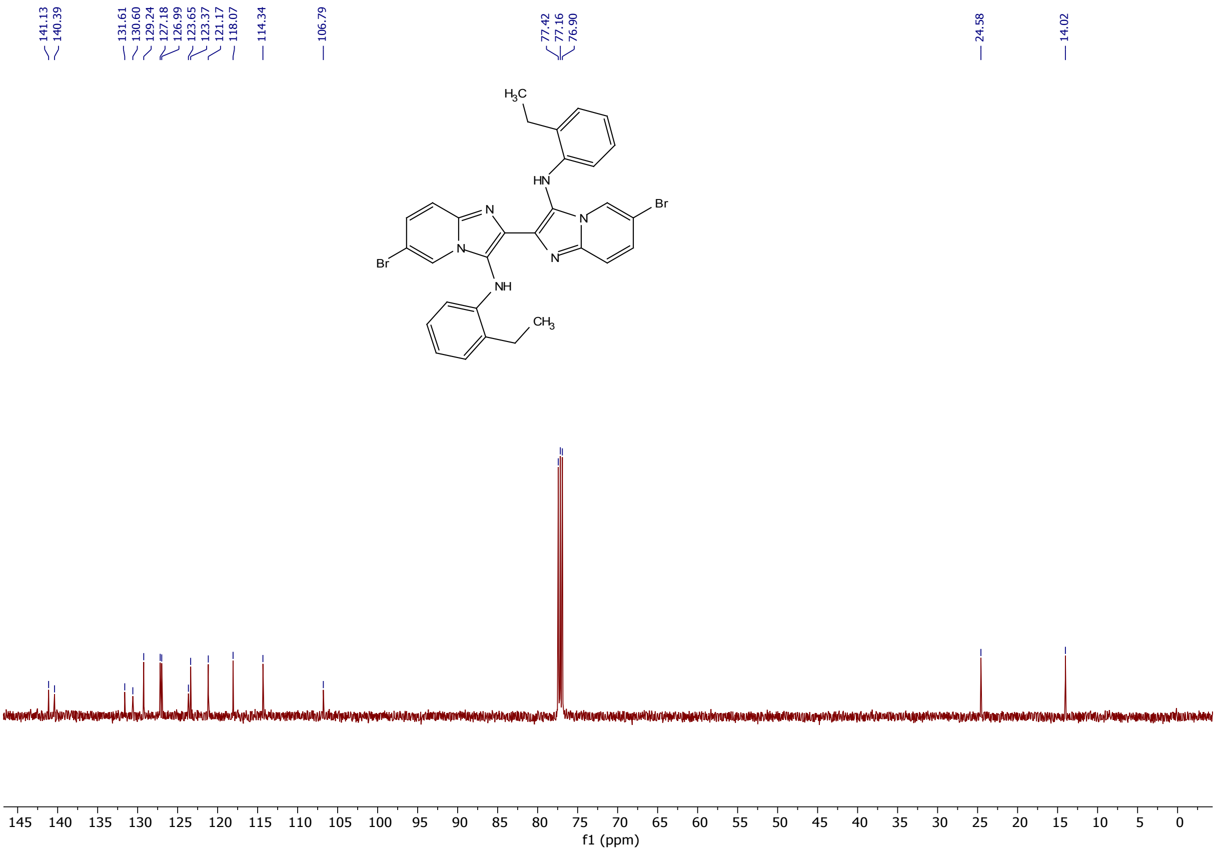

^{1}H NMR spectrum of **3bd** (500 MHz, $CDCl_3$)

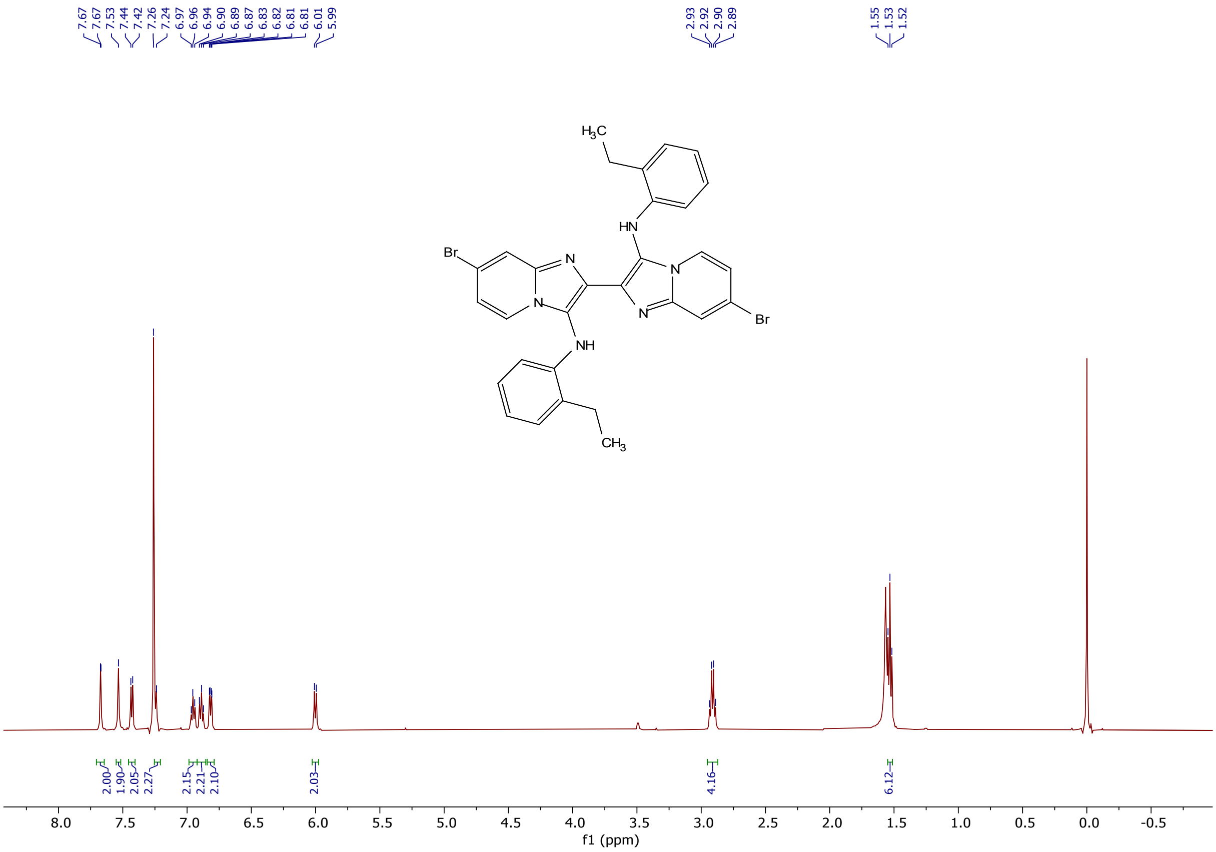

$^{13}C\{^{1}H\}$ NMR spectrum of **3bd** (126 MHz, $CDCl_3$)

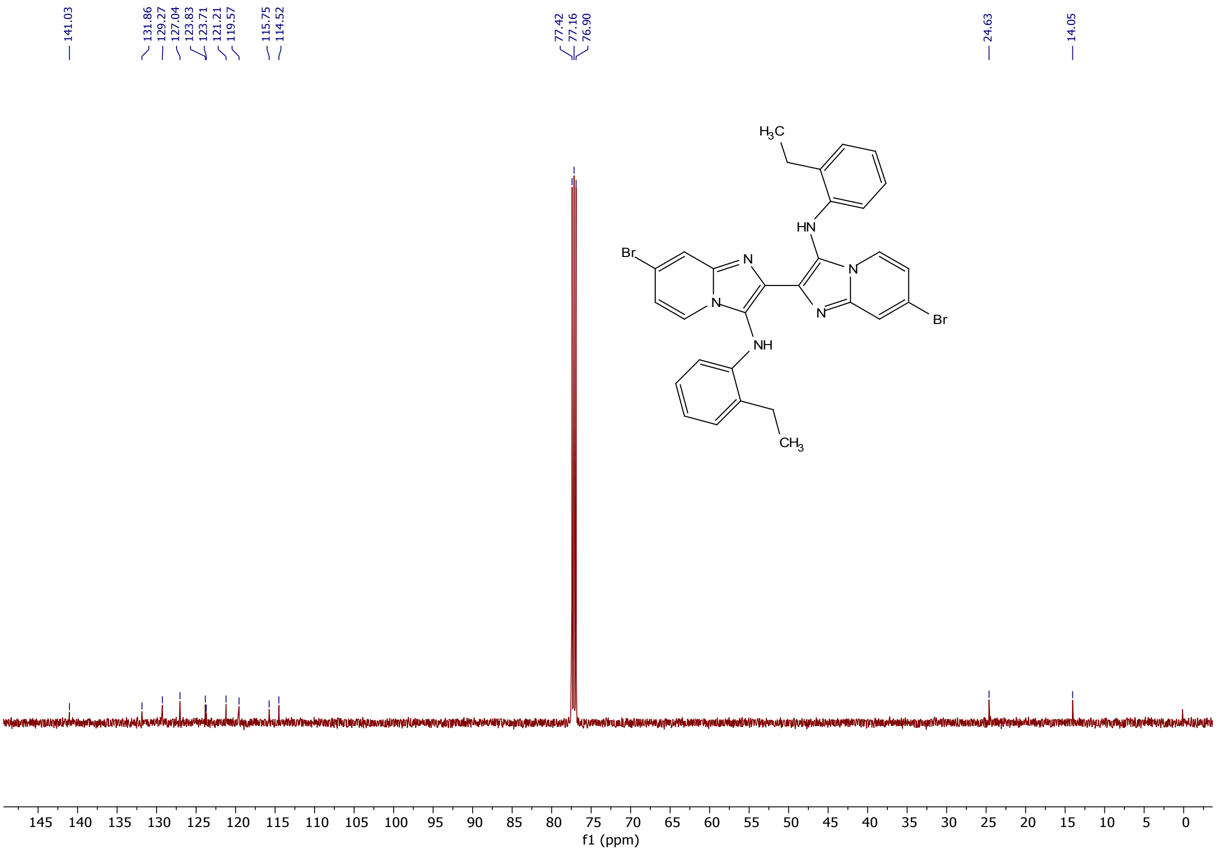

^{1}H NMR spectrum of **3be** (500 MHz, $CDCl_3$)

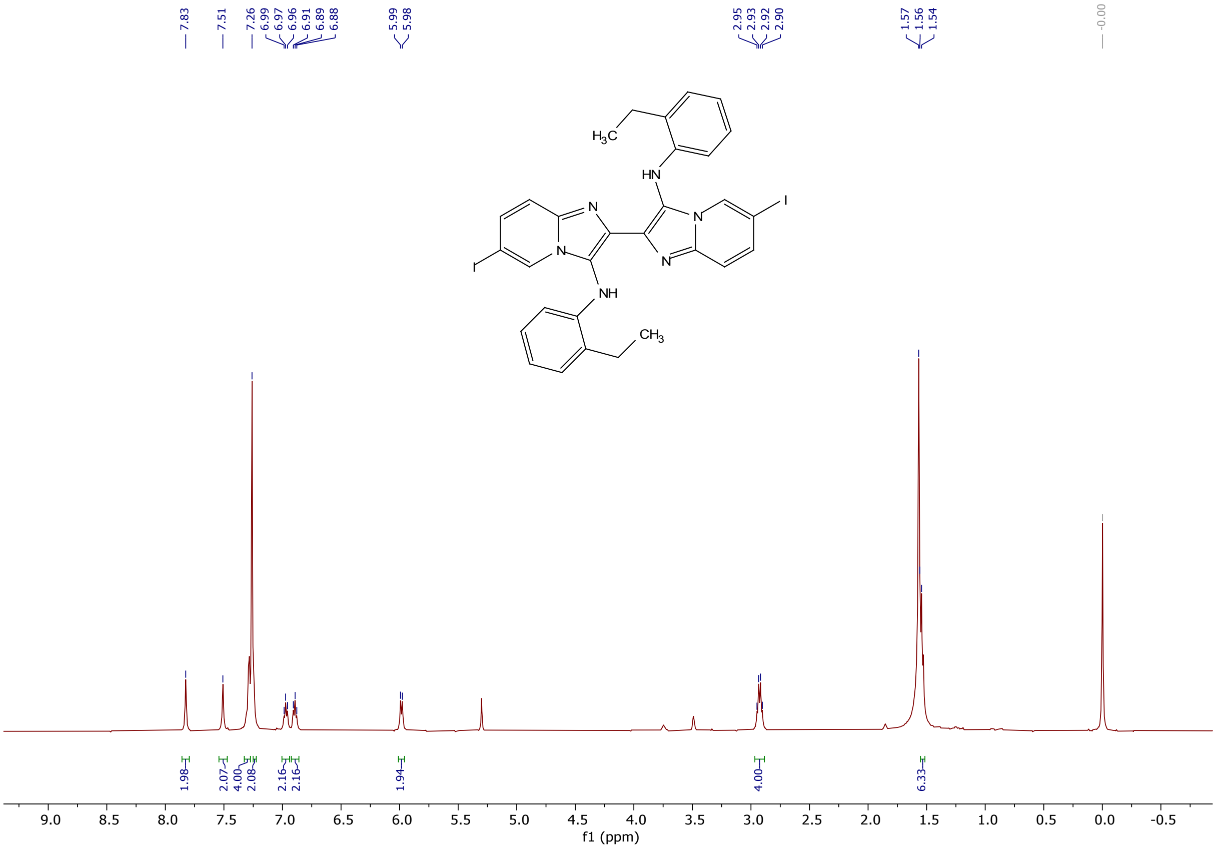

$^{13}C\{^1H\}$ NMR spectrum of **3be** (126 MHz, $CDCl_3$)

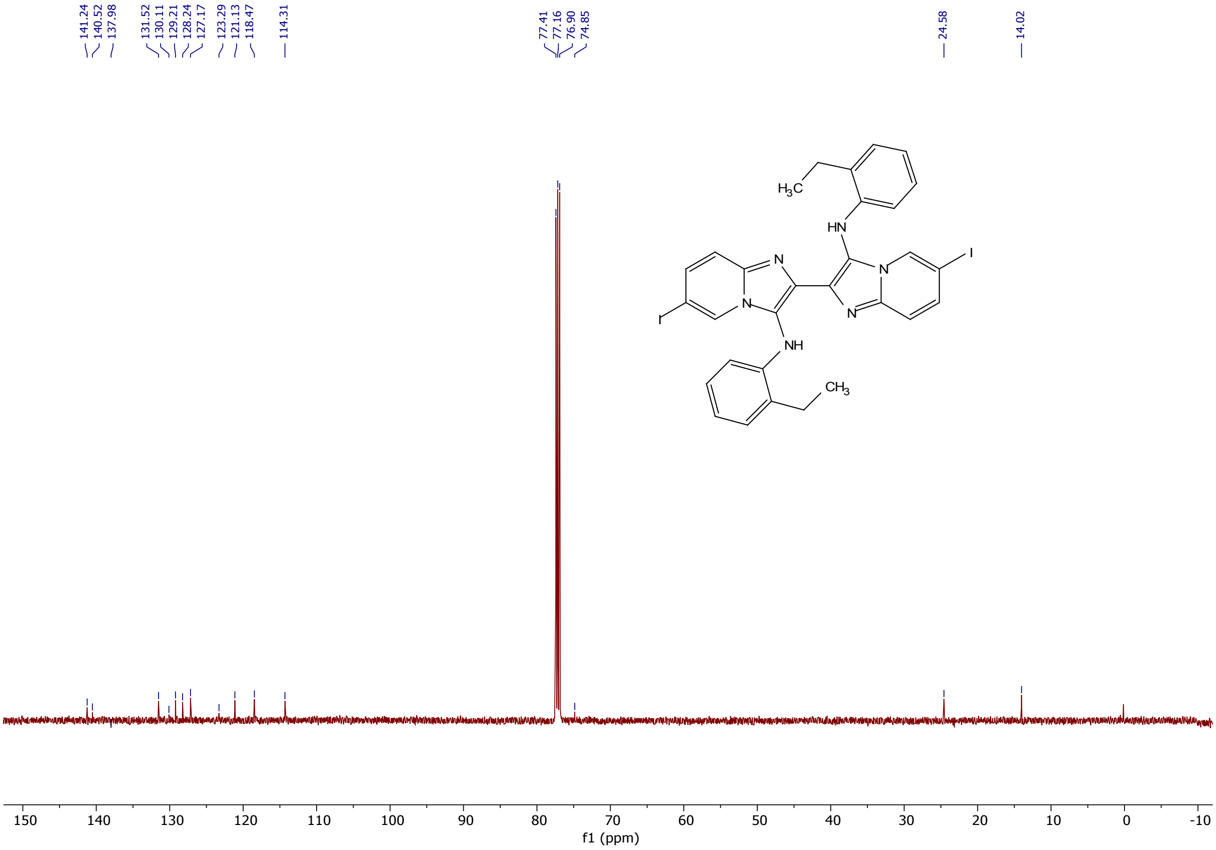

^1H NMR spectrum of **3bf** (500 MHz, $CDCl_3$)

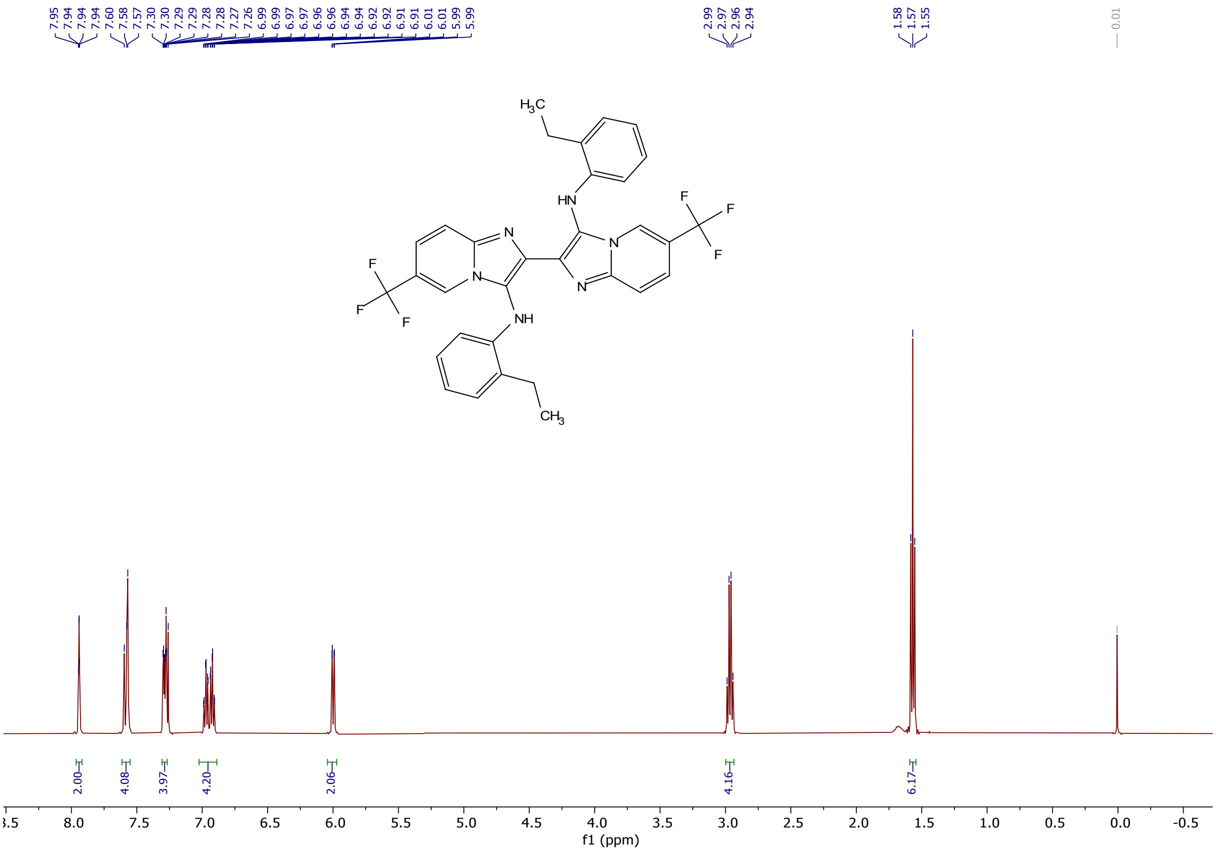

$^{13}C\{^1H\}$ NMR spectrum of **3bf** (126 MHz, $CDCl_3$)

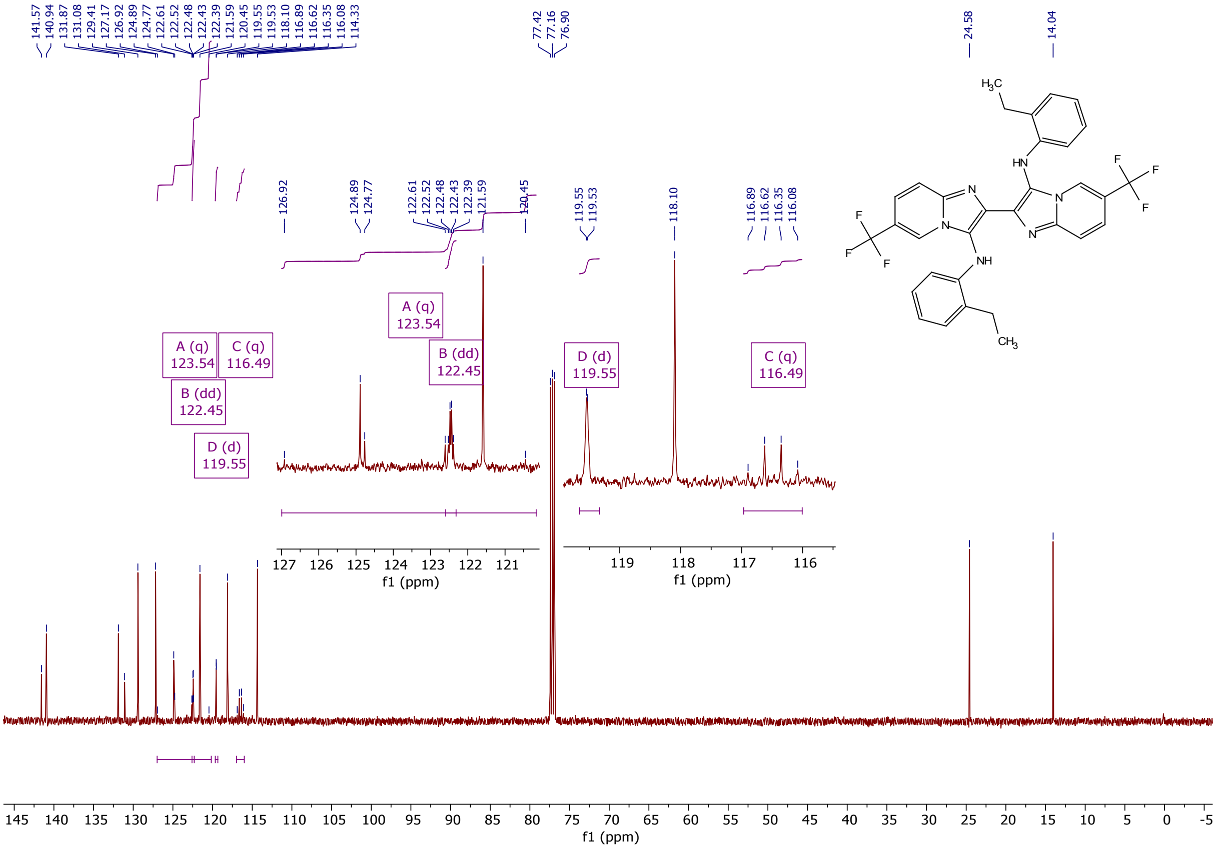

^1H NMR spectrum of **3bg** (500 MHz, $CDCl_3$)

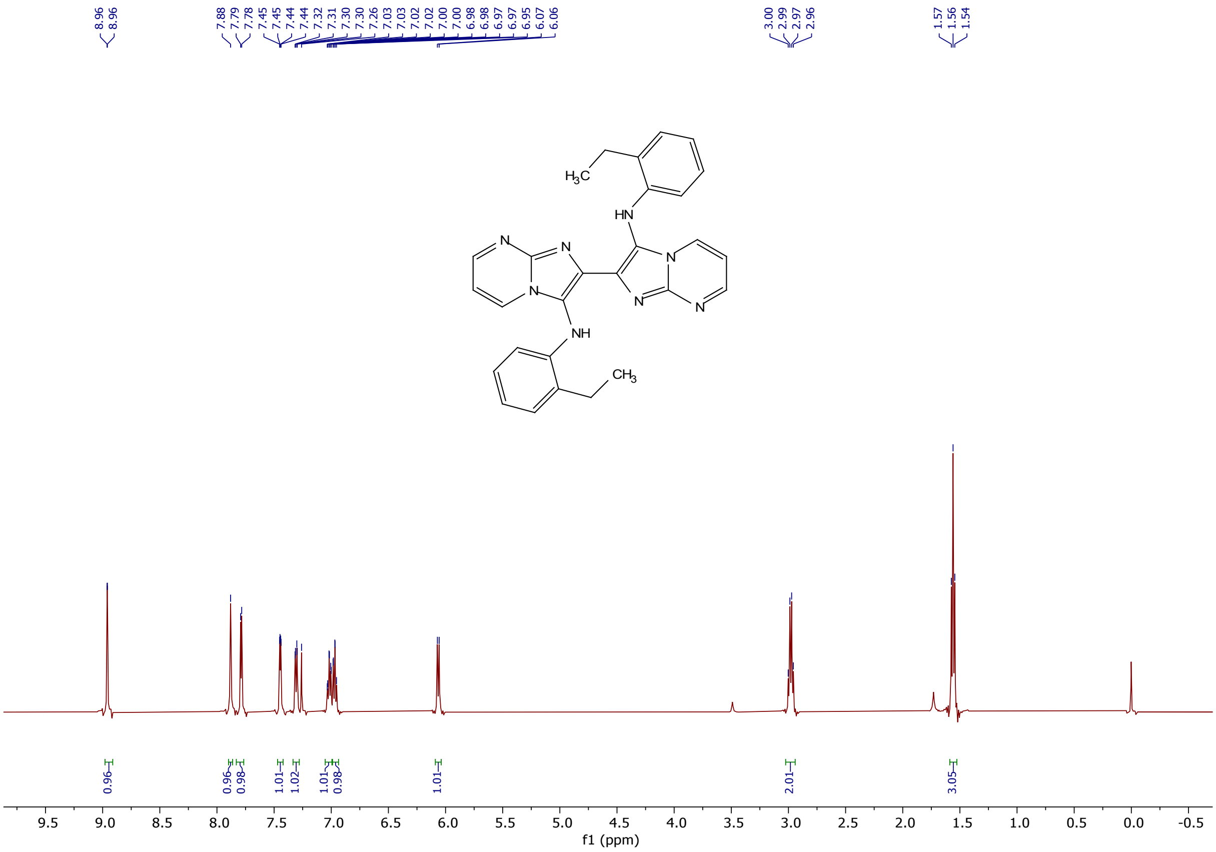

$^{13}C\{^{1}H\}$ NMR spectrum of **3bg** (126 MHz, $CDCl_3$)

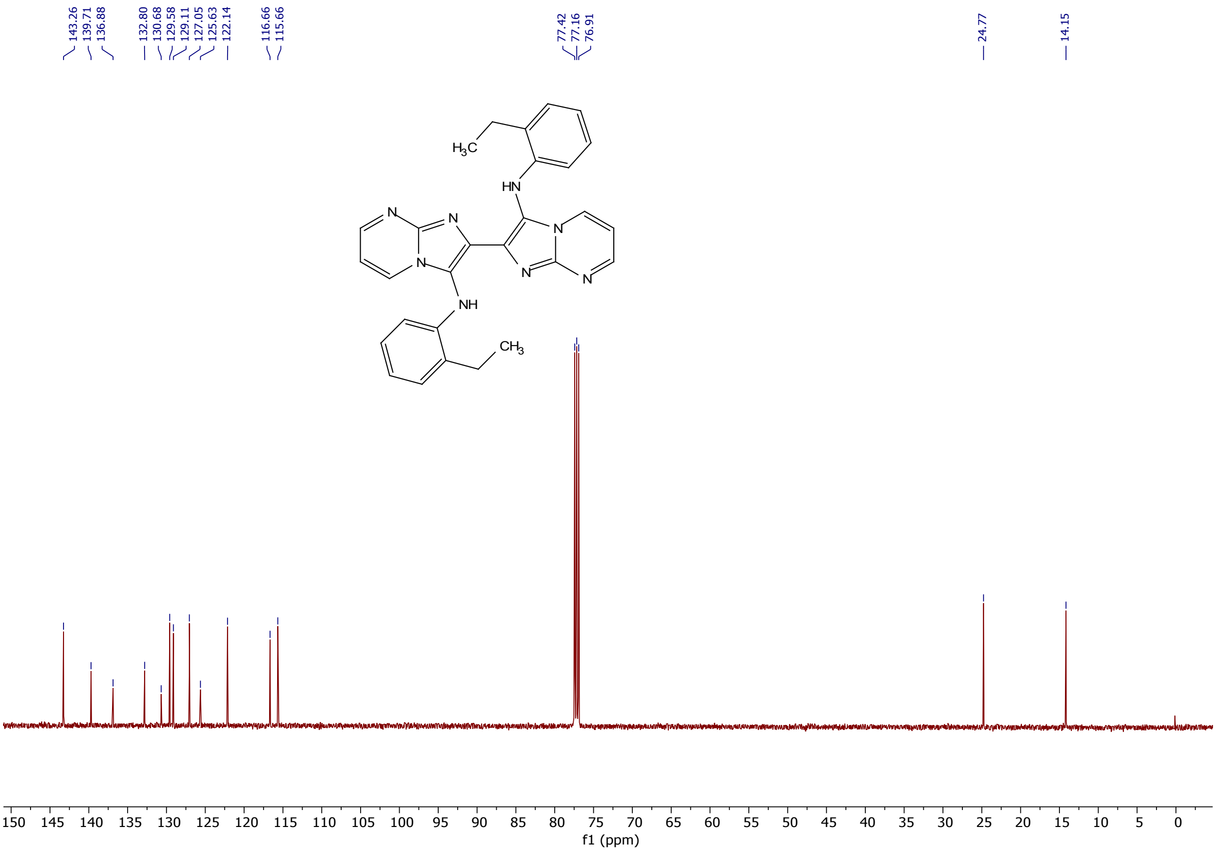

^{1}H NMR spectrum of **3bh** (500 MHz, $CDCl_3$)

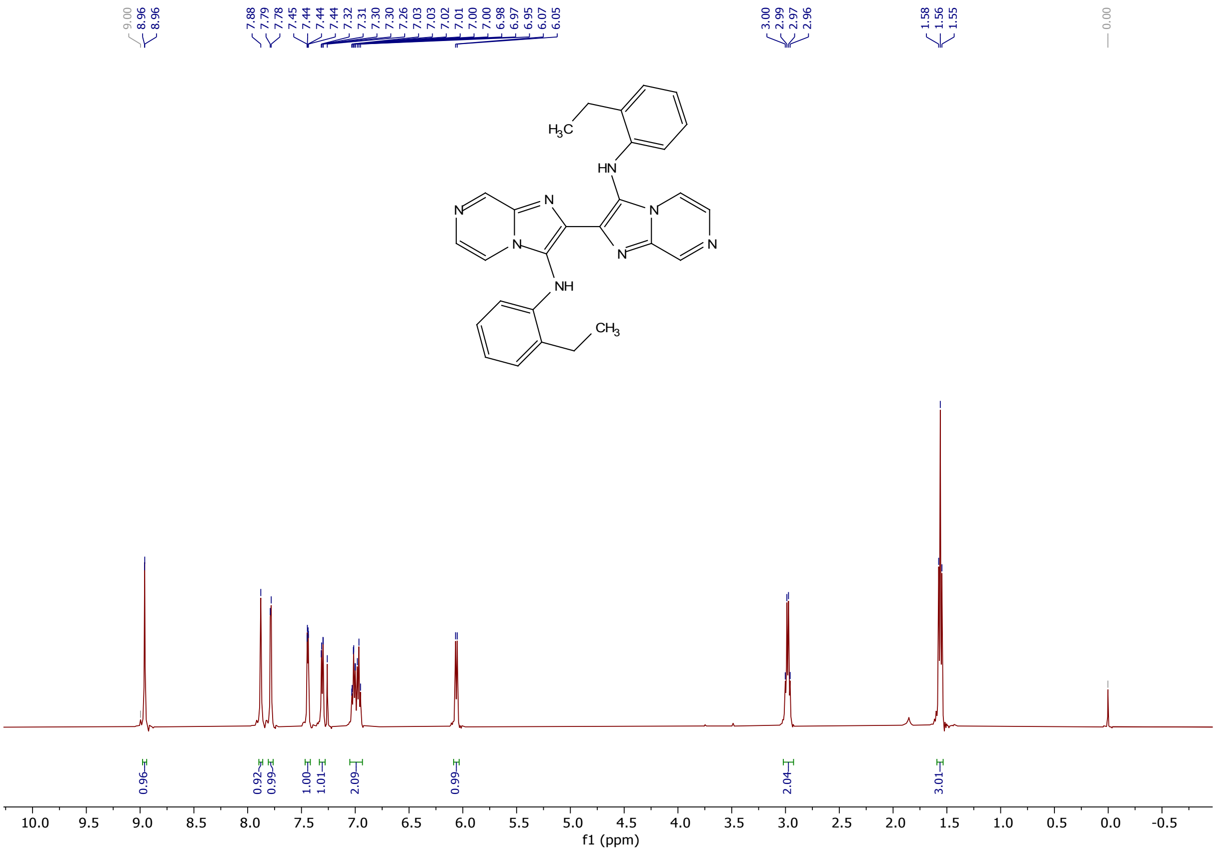

$^{13}C\{^{1}H\}$ NMR spectrum of **3bh** (126 MHz, $CDCl_3$)

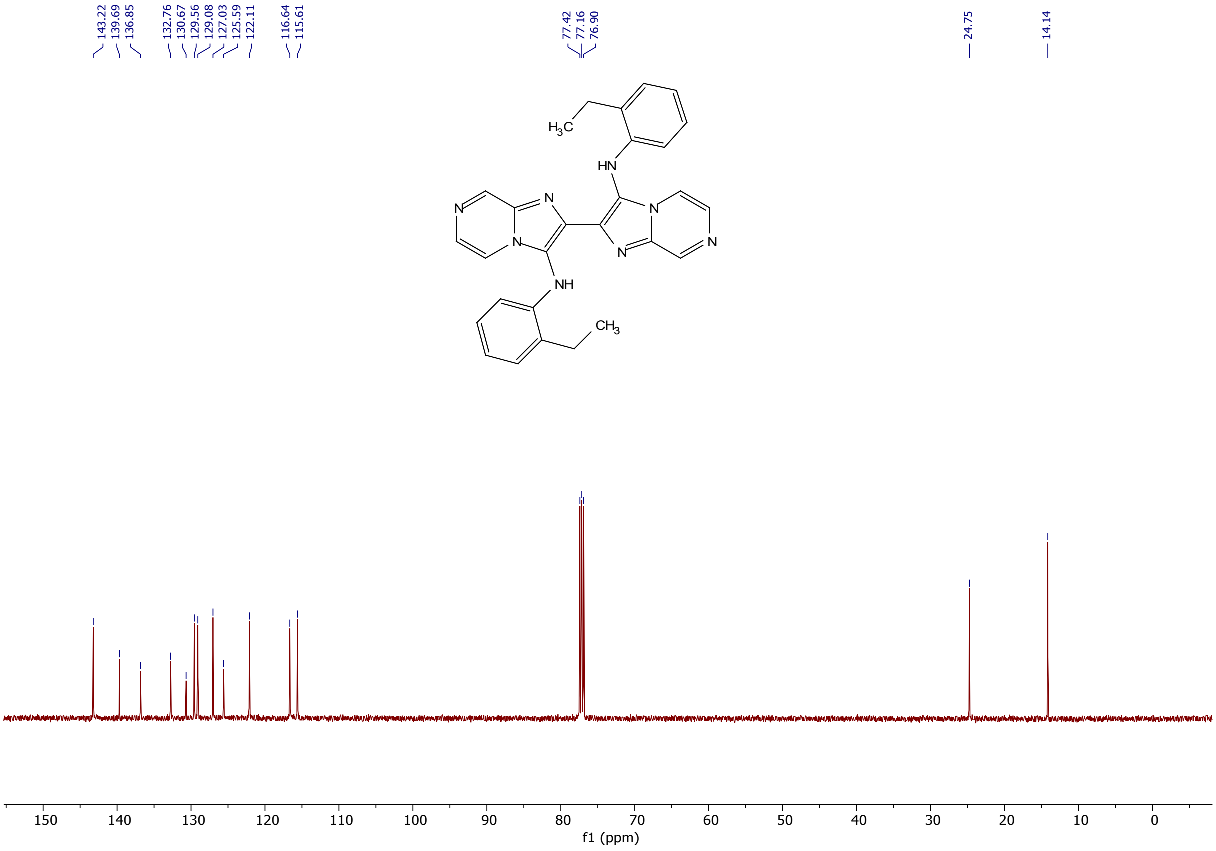

^{1}H NMR spectrum of **3bi** (500 MHz, $CDCl_3$)

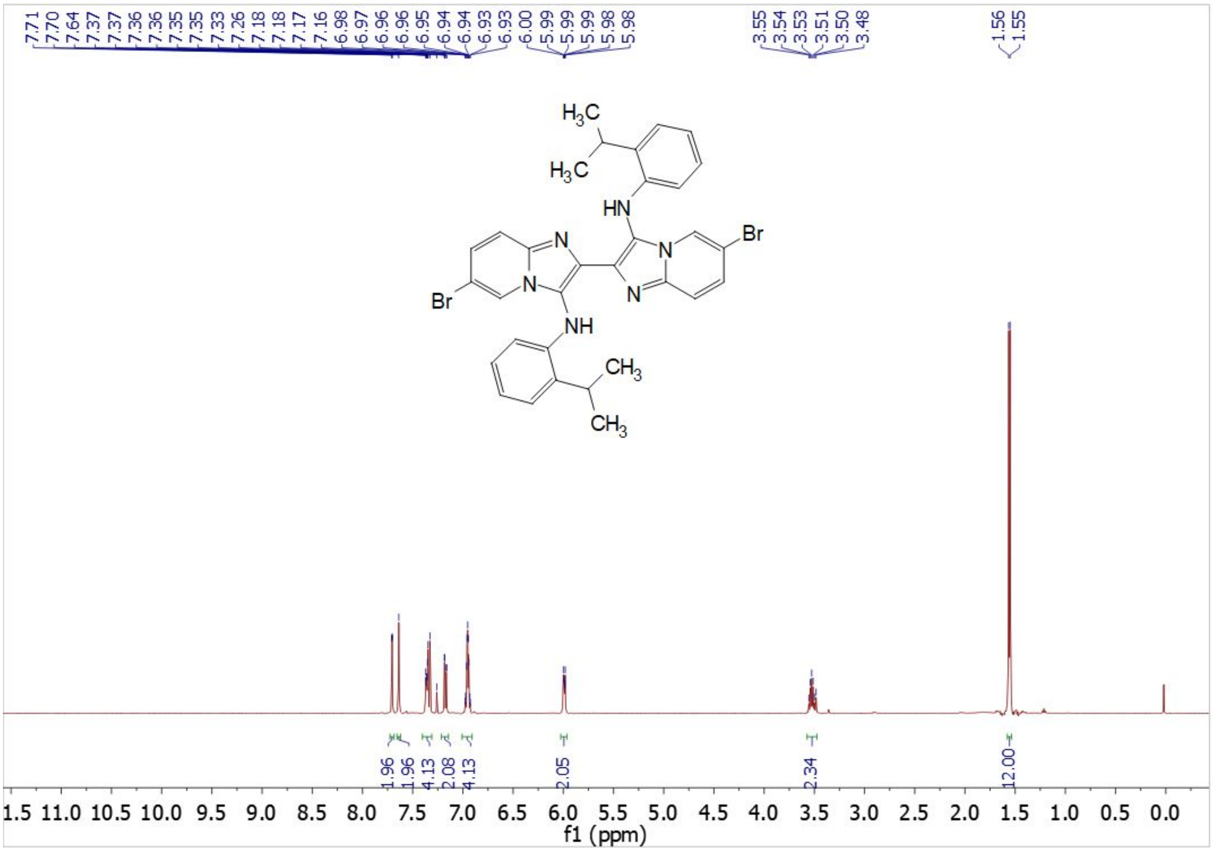

$^{13}C\{^1H\}$ NMR spectrum of **3bi** (126 MHz, $CDCl_3$)

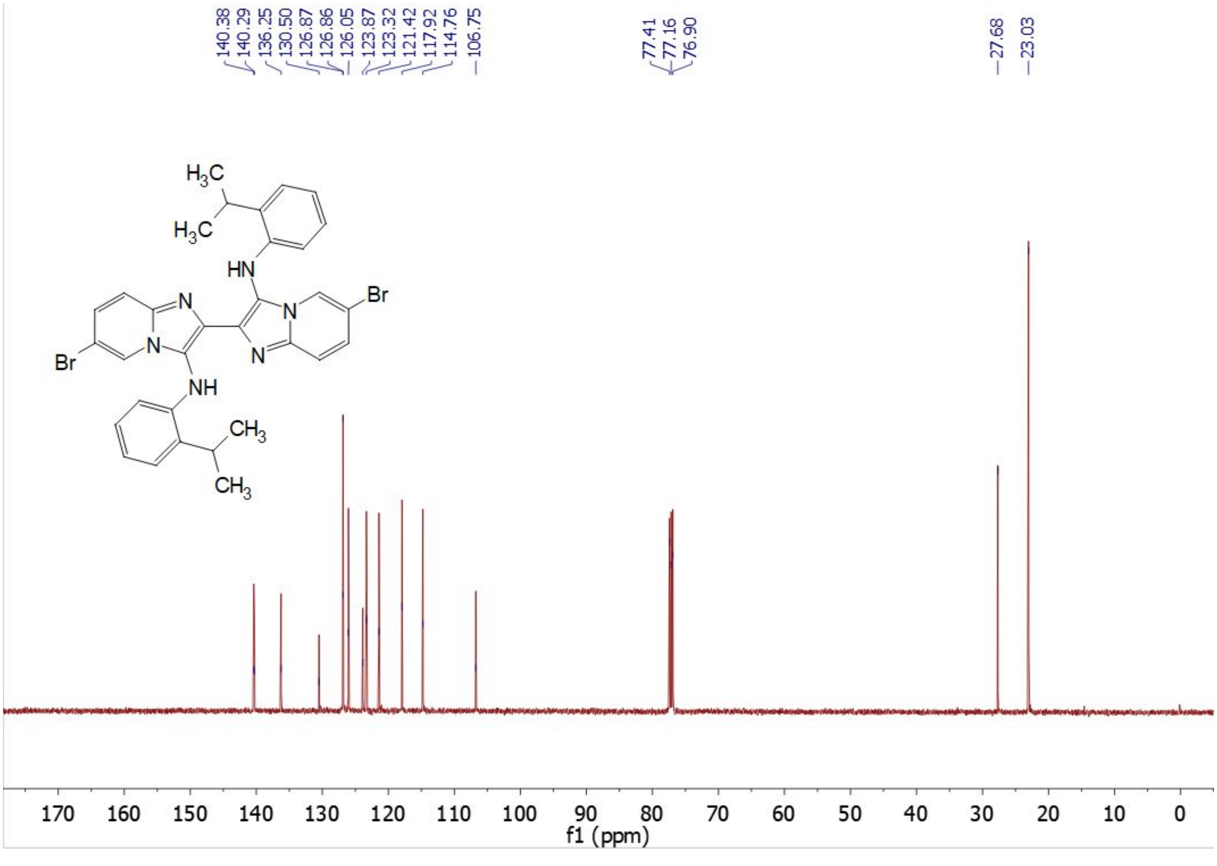

^1H NMR spectrum of **3bj** (500 MHz, $CDCl_3$)

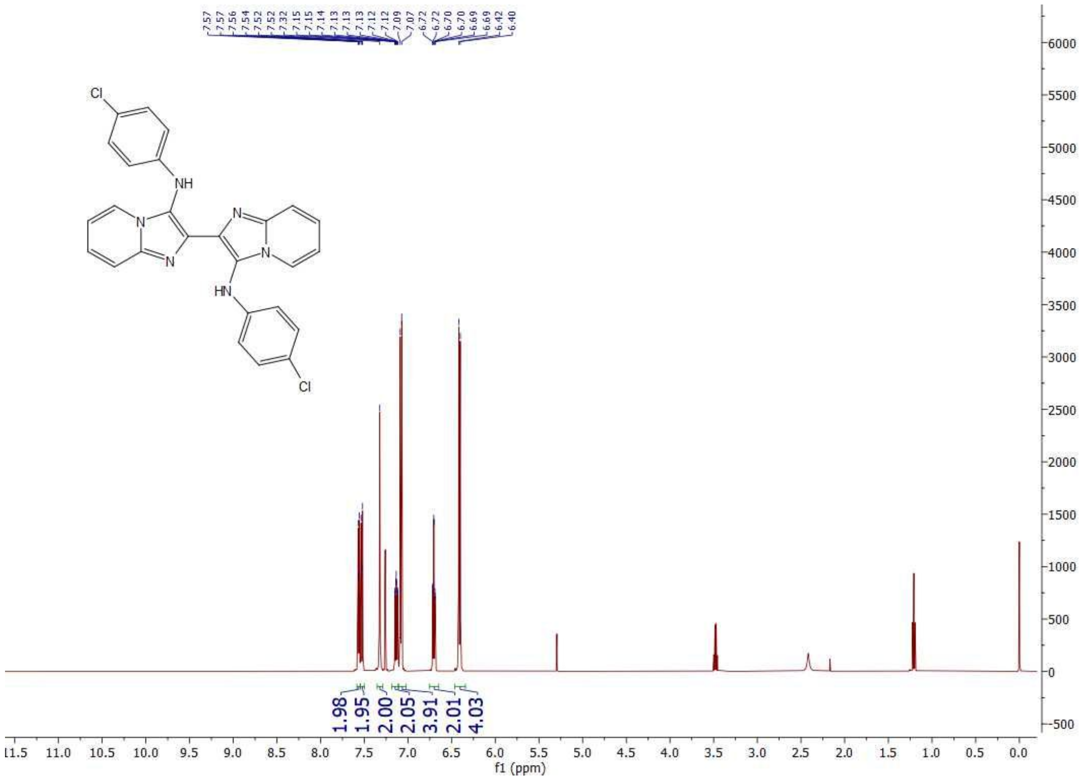

$^{13}C\{^{1}H\}$ NMR spectrum of **3bj** (126 MHz, $CDCl_3$)

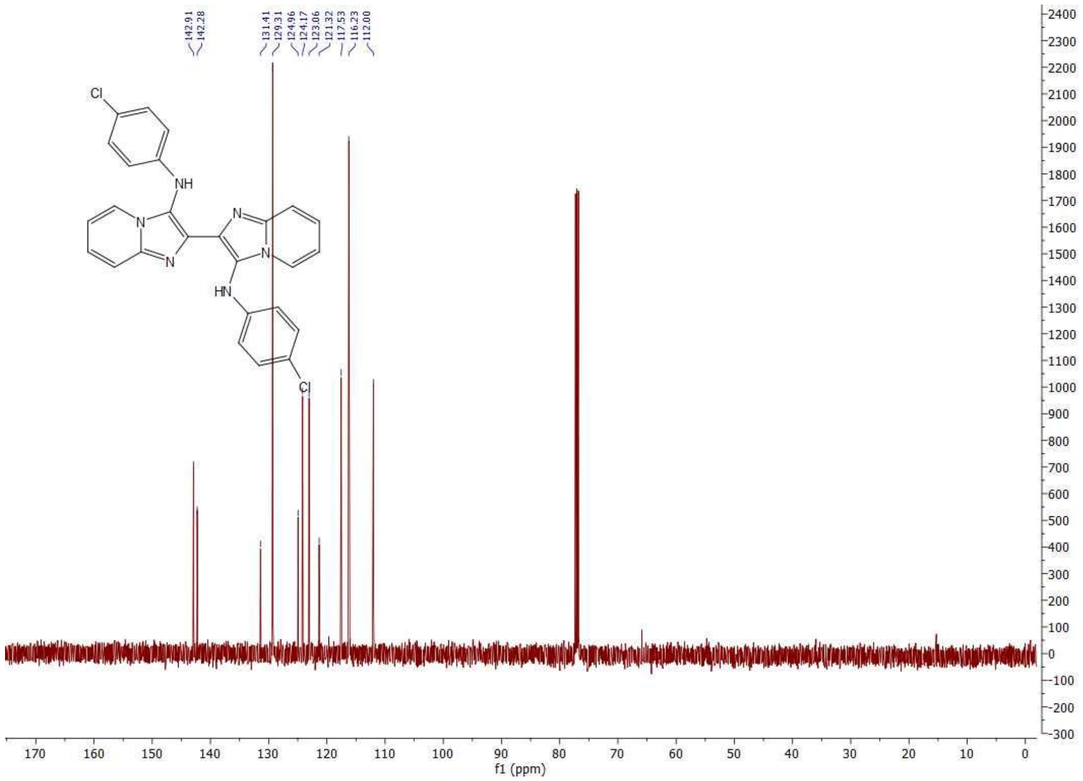

^{1}H NMR spectrum of **3bk** (500 MHz, $CDCl_3$)

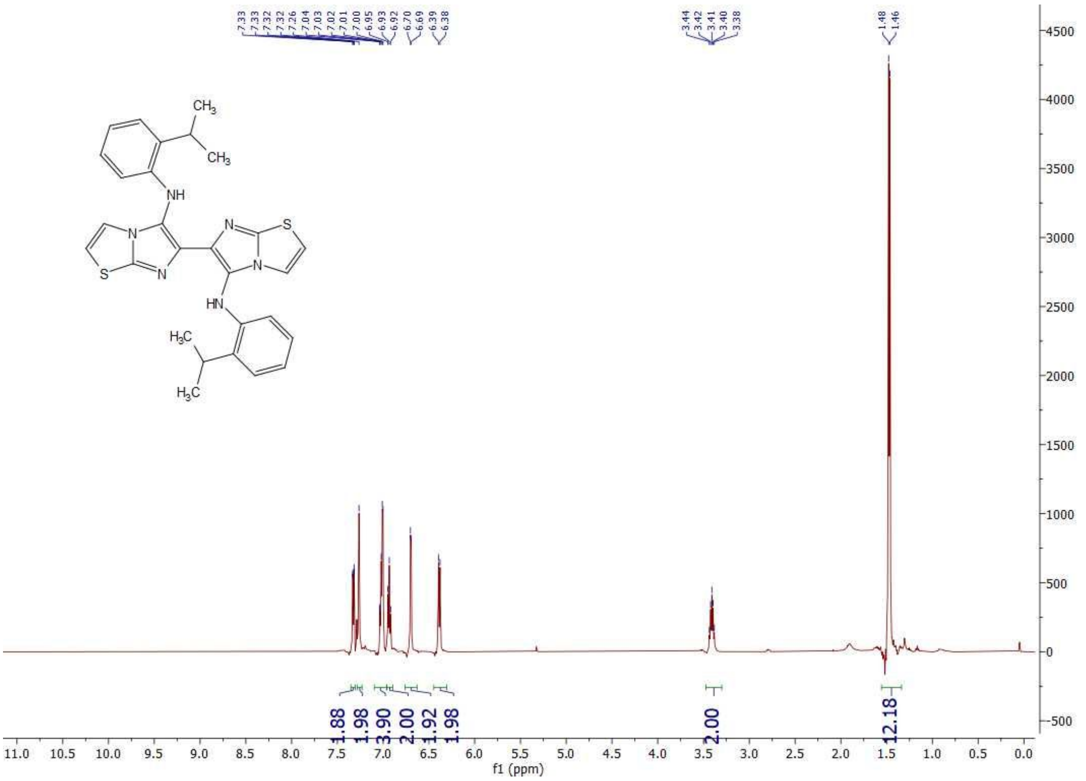

$^{13}C\{^{1}H\}$ NMR spectrum of **3bk** (126 MHz, $CDCl_3$)

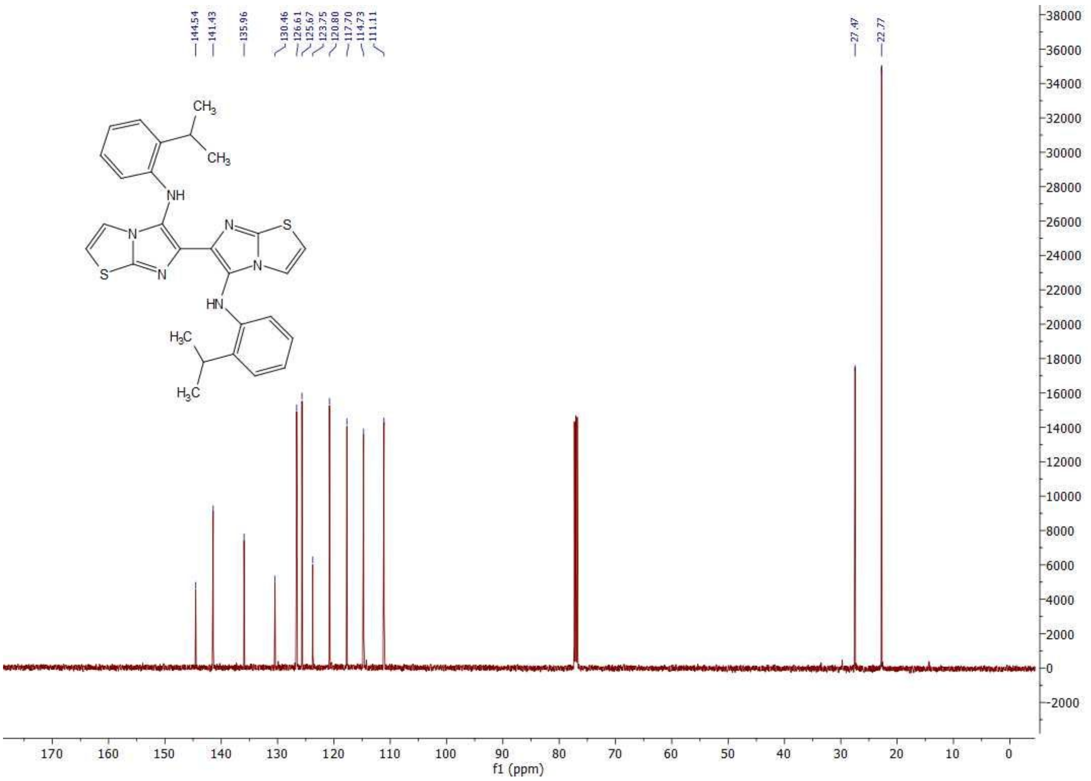

^{1}H NMR spectrum of **3bl** (500 MHz, DMSO-d_6)

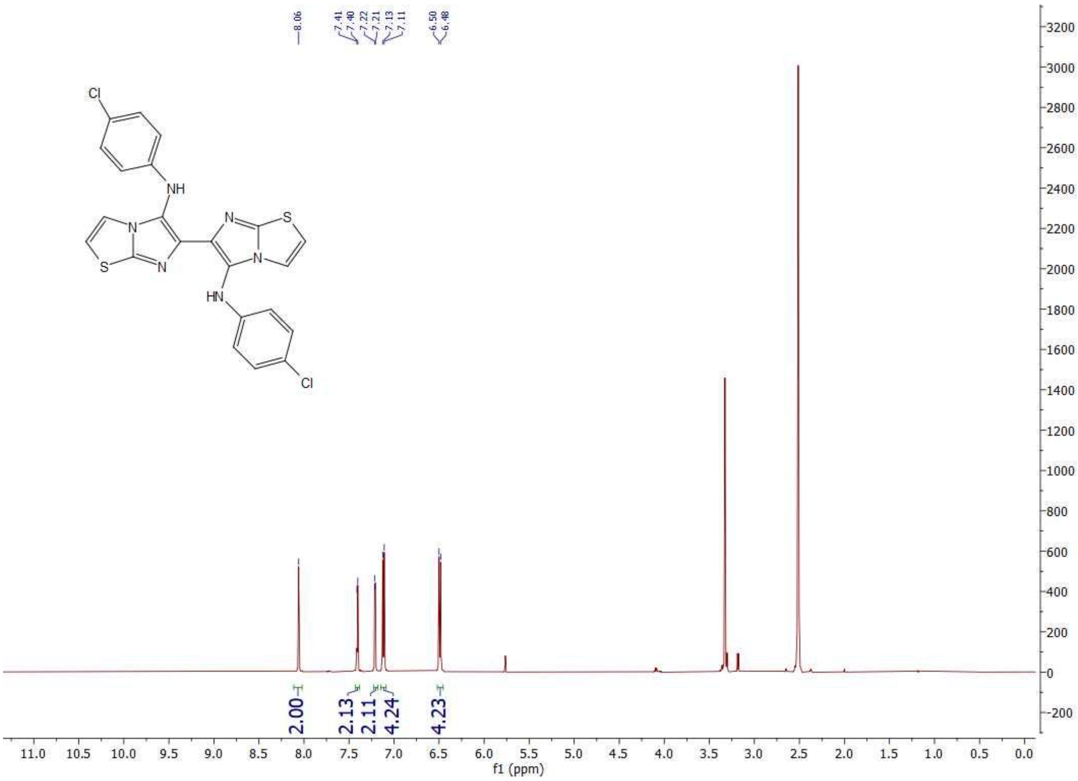

$^{13}C\{^{1}H\}$ NMR spectrum of **3bl** (126 MHz, DMSO-d_6)

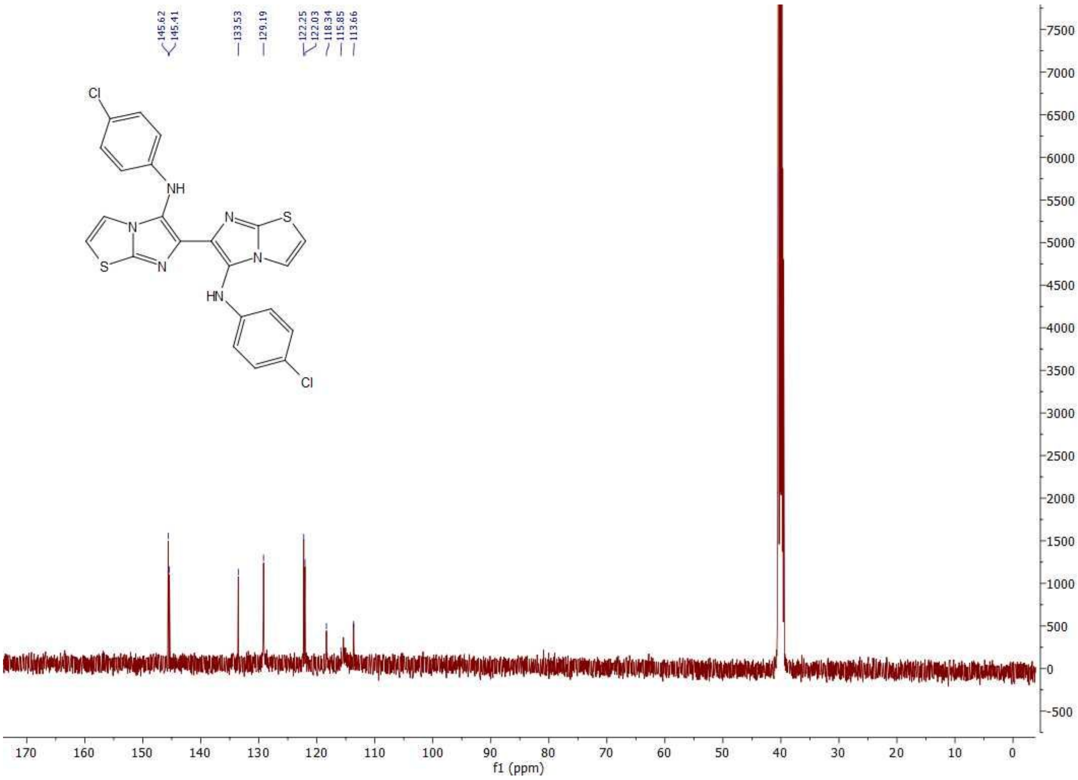

^{1}H NMR spectrum of **3bm** (500 MHz, $CDCl_3$)

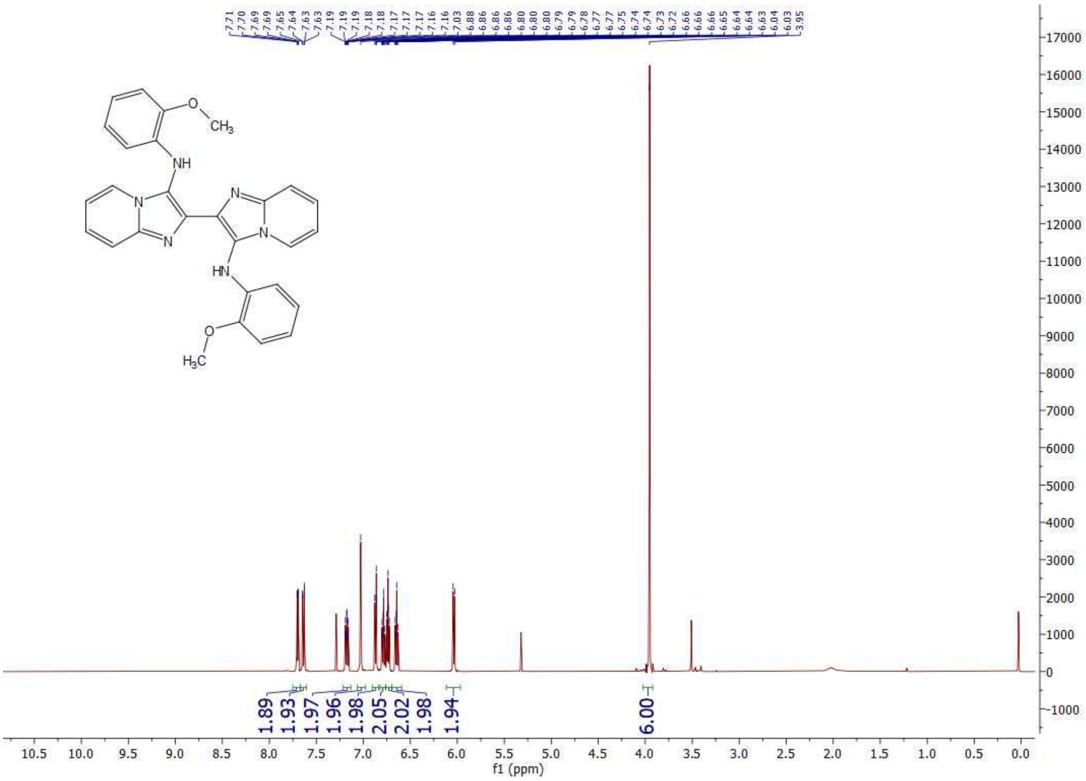

$^{13}C\{^{1}H\}$ NMR spectrum of **3bm** (126 MHz, $CDCl_3$)

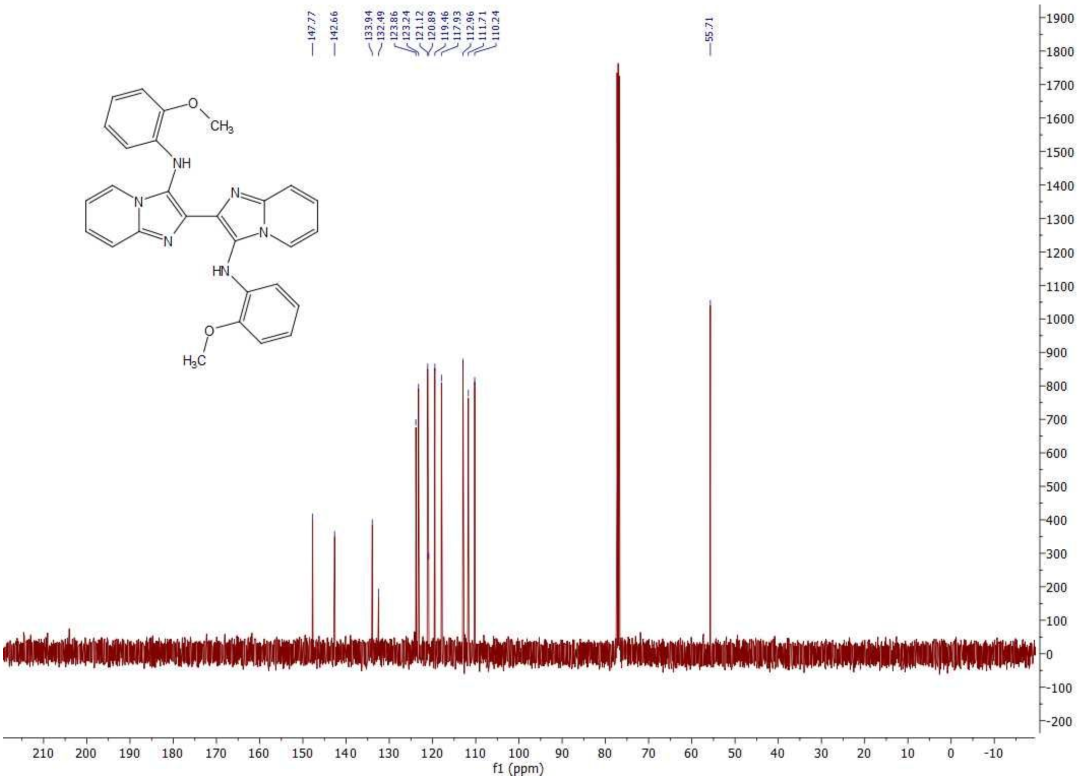

^{1}H NMR spectrum of **3bn** (500 MHz, $CDCl_3$)

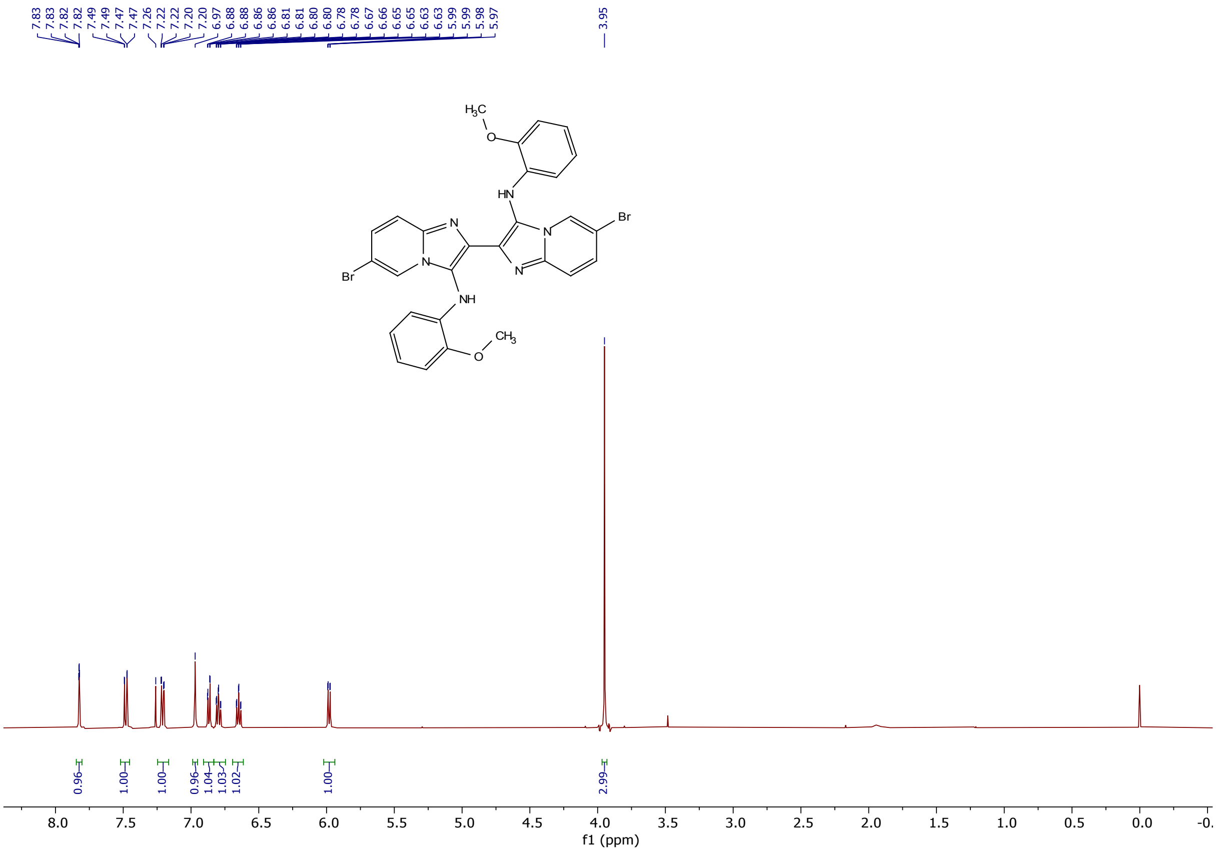

$^{13}C\{^{1}H\}$ NMR spectrum of **3bn** (126 MHz, $CDCl_3$)

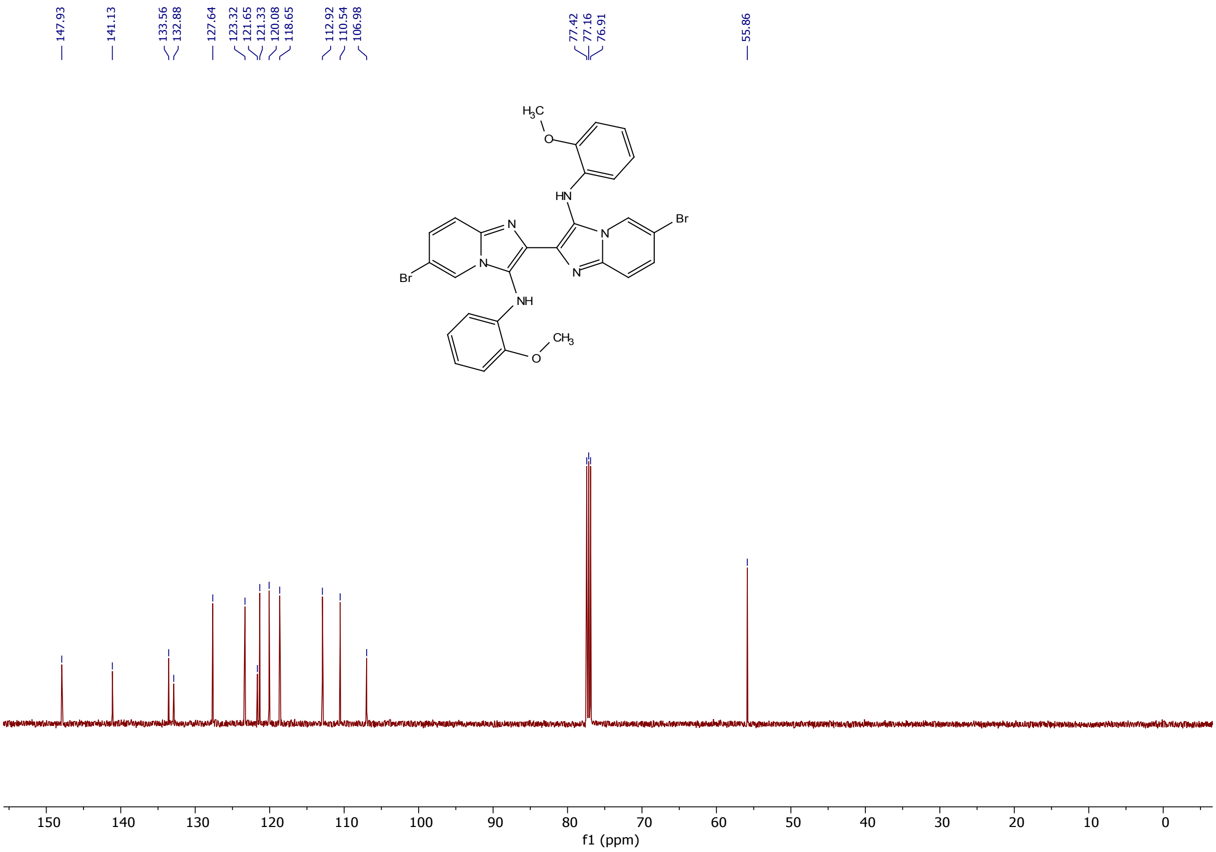

^{1}H NMR spectrum of **3bo** (500 MHz, DMSO-d_6)

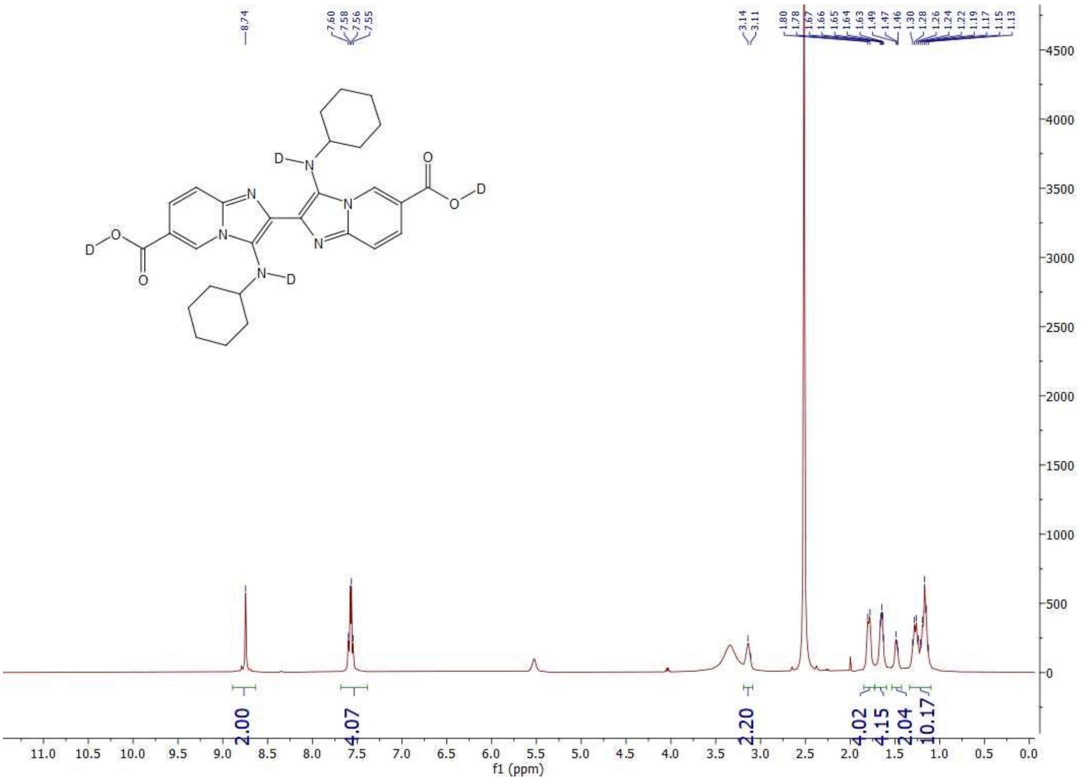

$^{13}C\{^{1}H\}$ NMR spectrum of **3bo** ((500 MHz, DMSO-d_6)

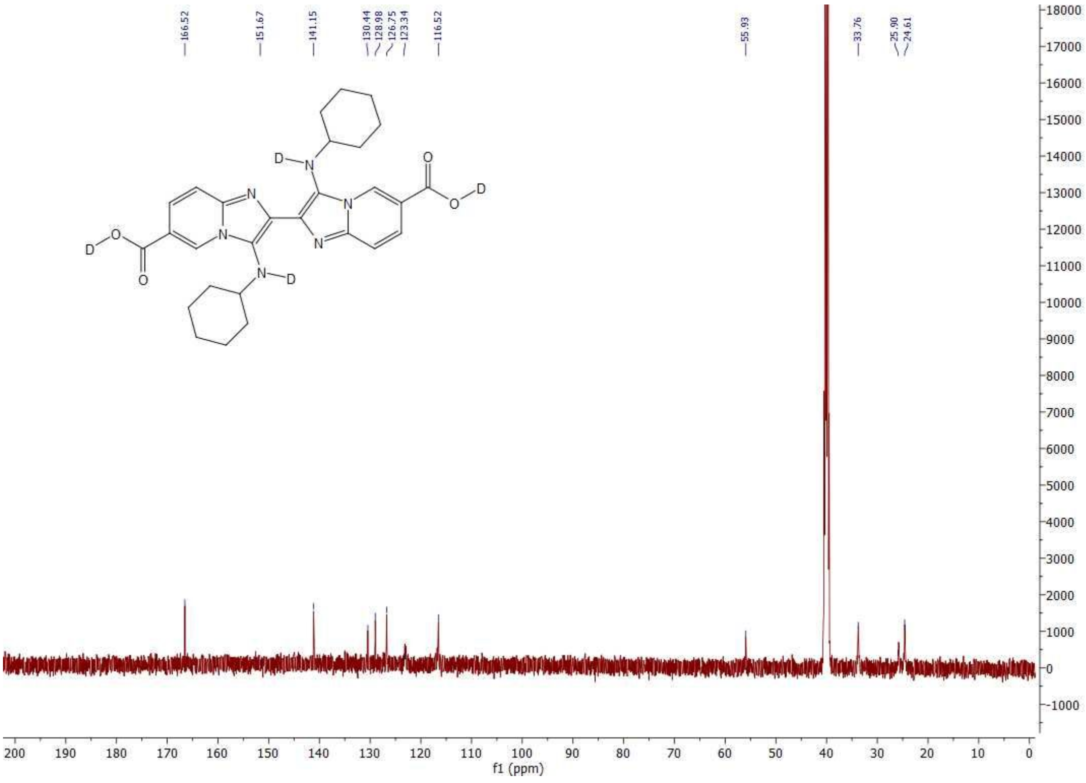

^{1}H NMR spectrum of **3bp** (500 MHz, $CDCl_3$)

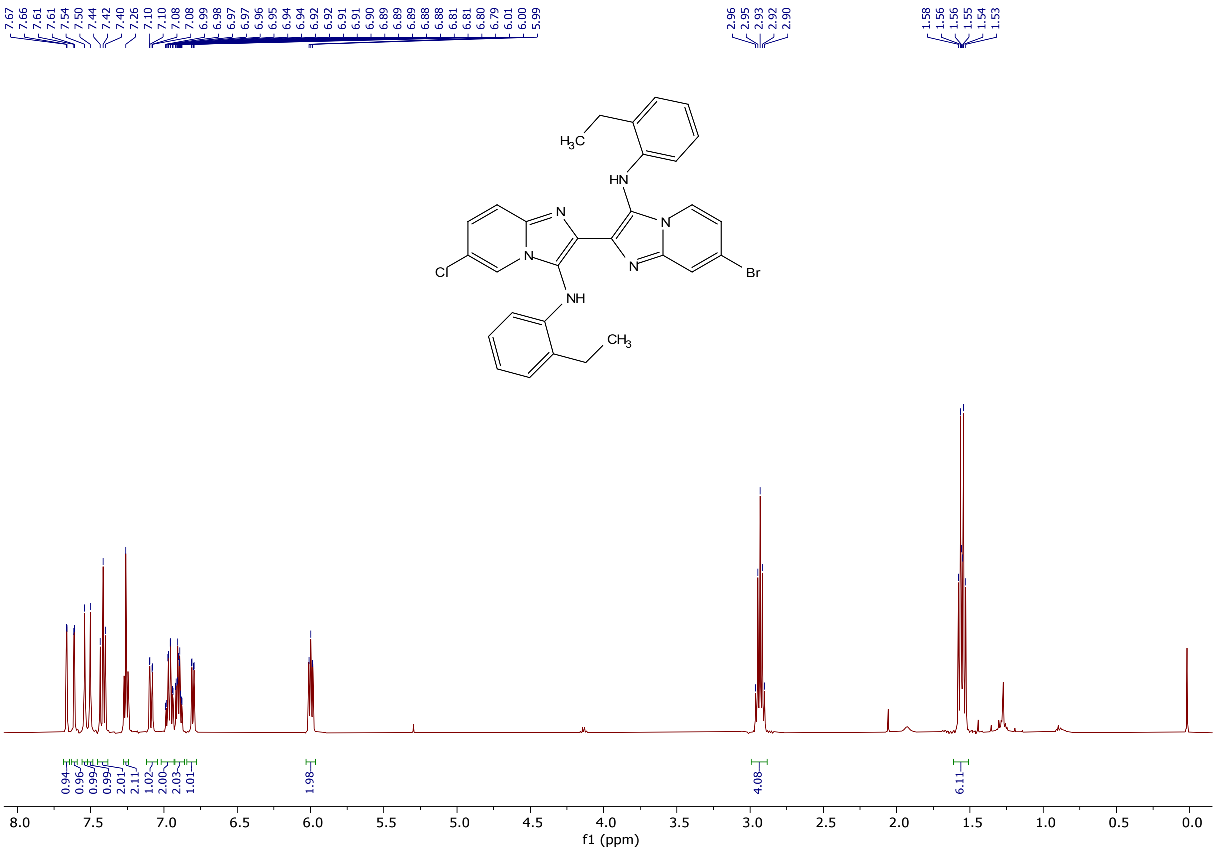

$^{13}C\{^{1}H\}$ NMR spectrum of **3bp** (126 MHz, $CDCl_3$)

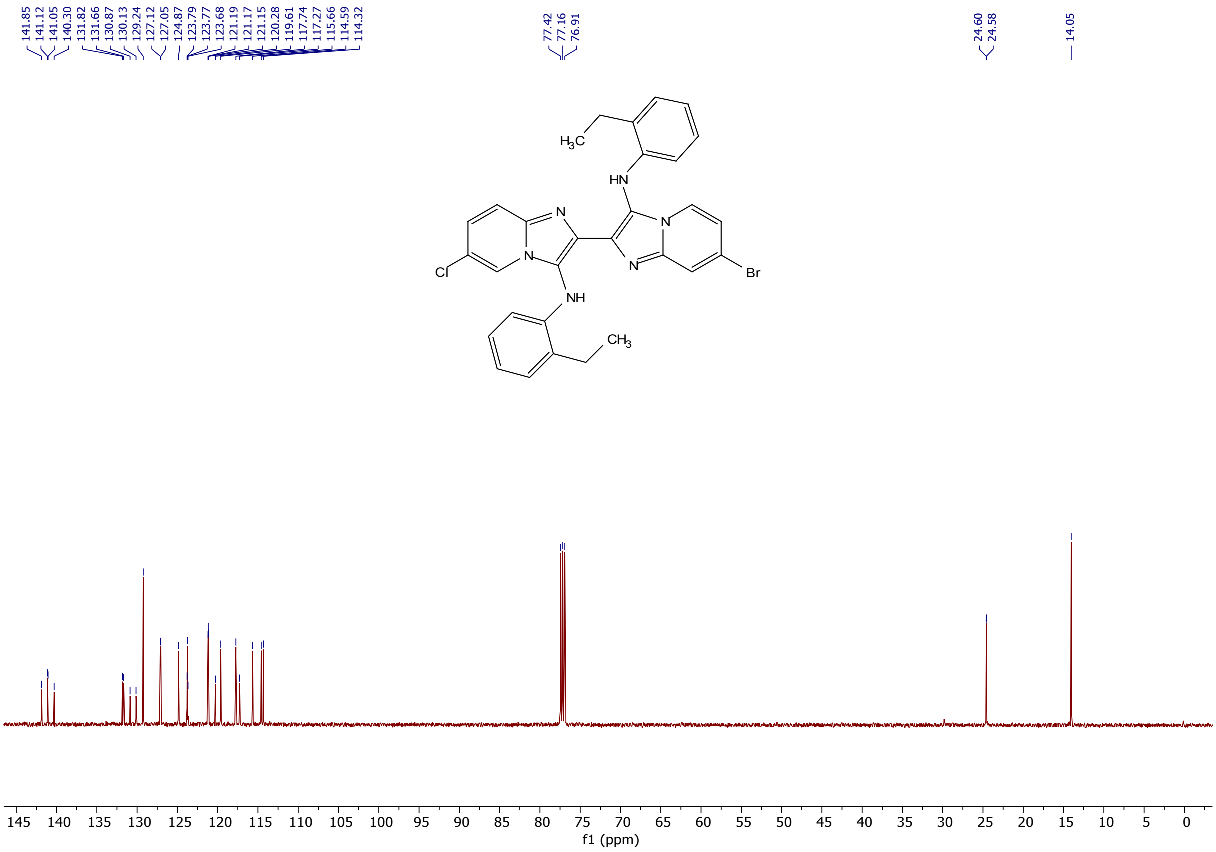

^{1}H NMR spectrum of **3bq** (500 MHz, $CDCl_3$)

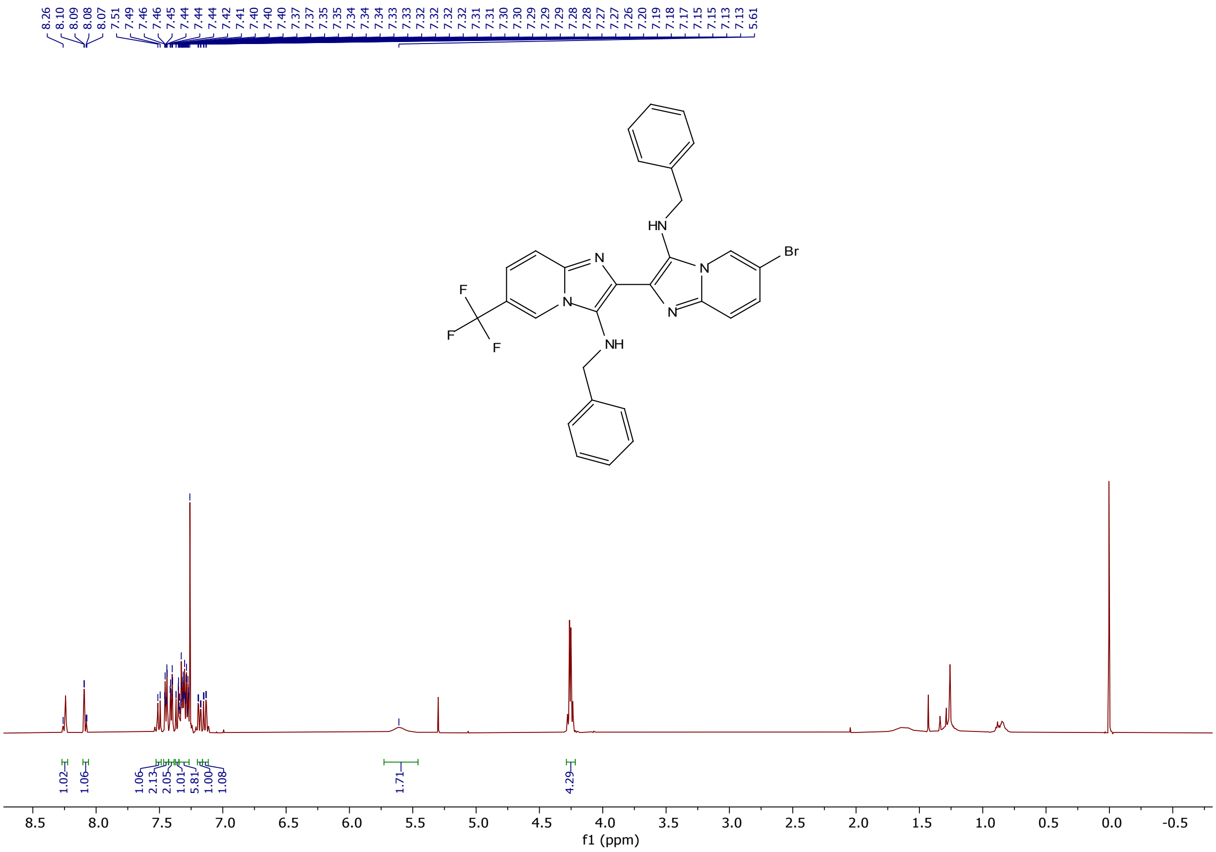

$^{13}C\{^{1}H\}$ NMR spectrum of **3bq** (126 MHz, $CDCl_3$)

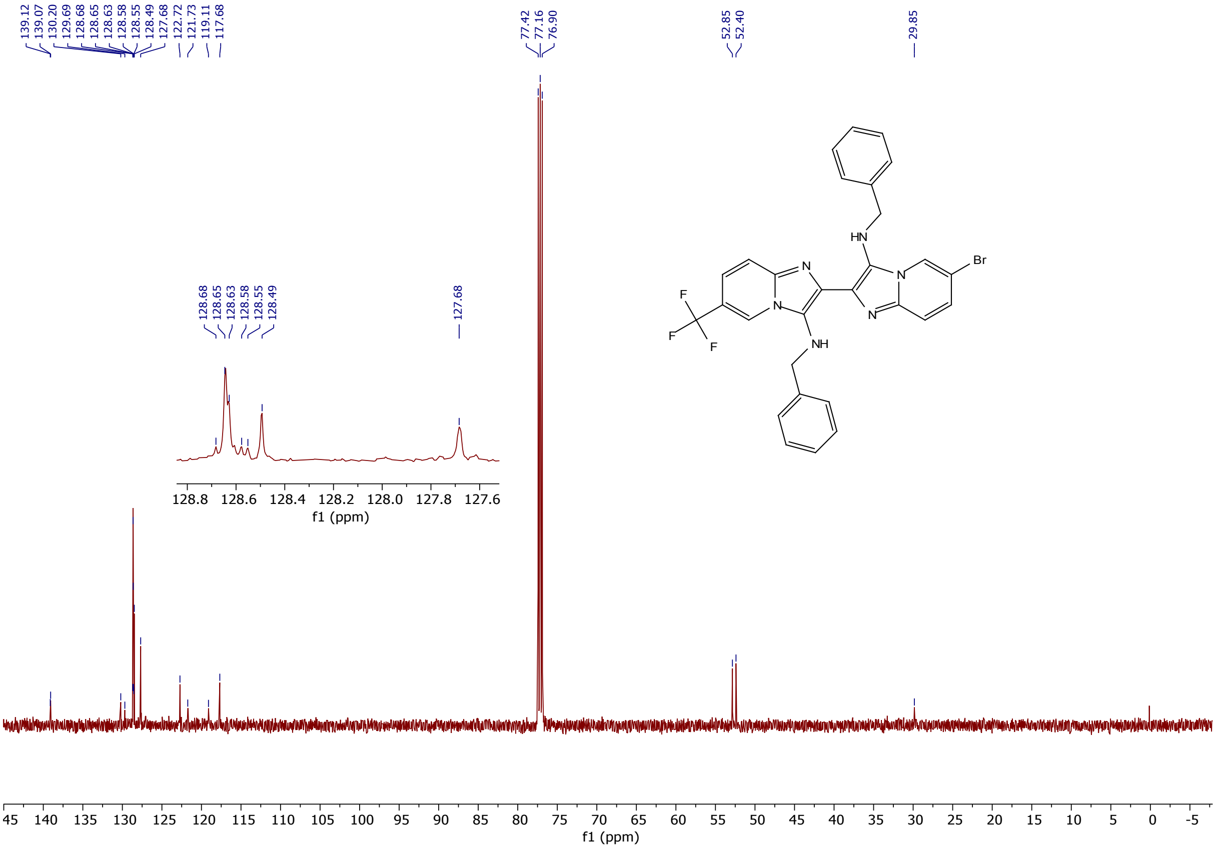

^{1}H NMR spectrum of **3br** (500 MHz, TFA-d)

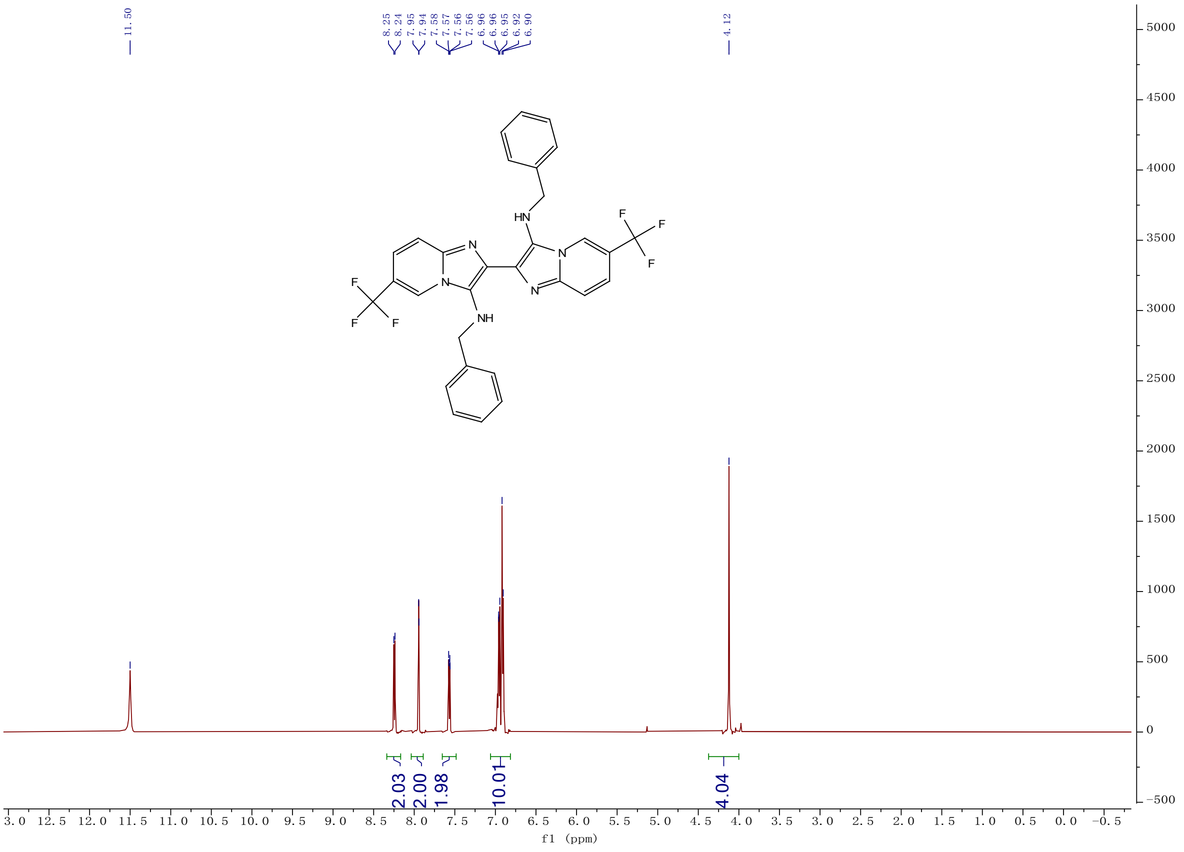

$^{13}C\{^{1}H\}$ NMR spectrum of **3br** (126 MHz, TFA-d)

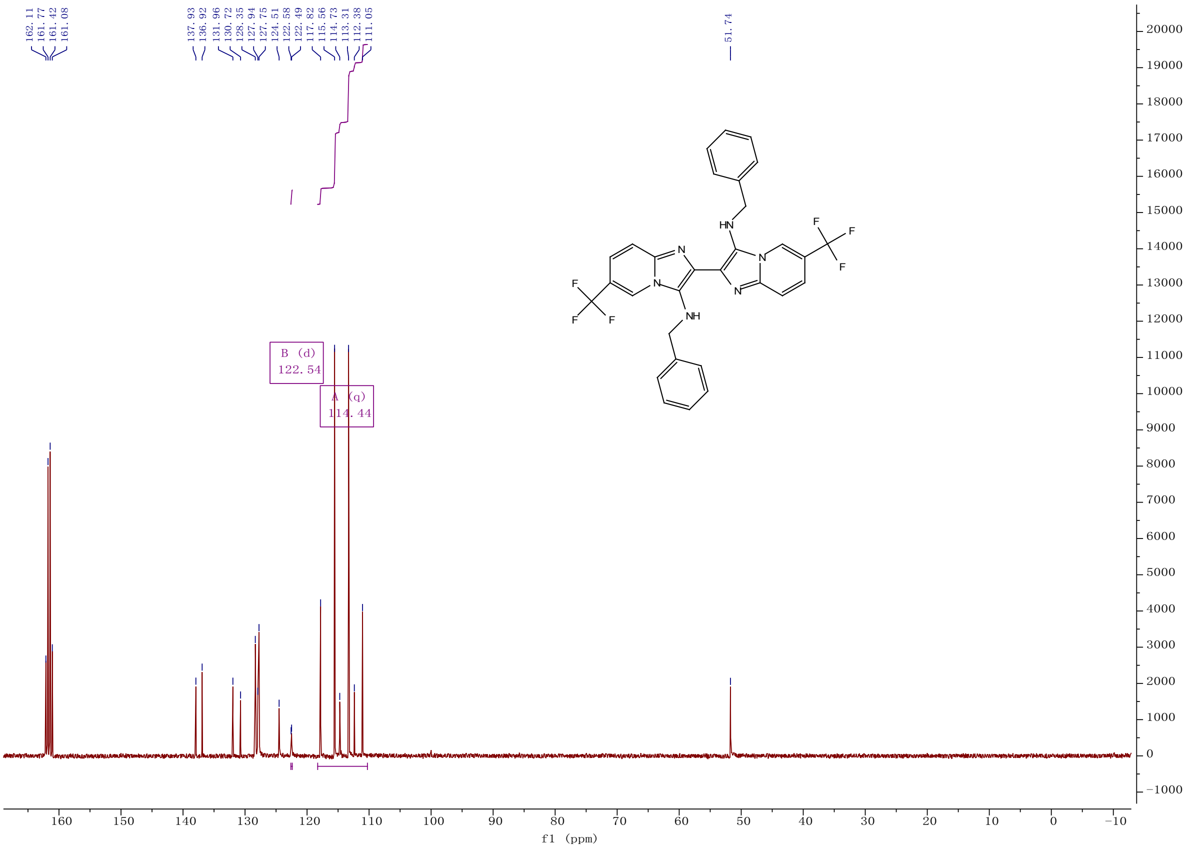

^{1}H NMR spectrum of **3bs** (500 MHz, TFA-d)

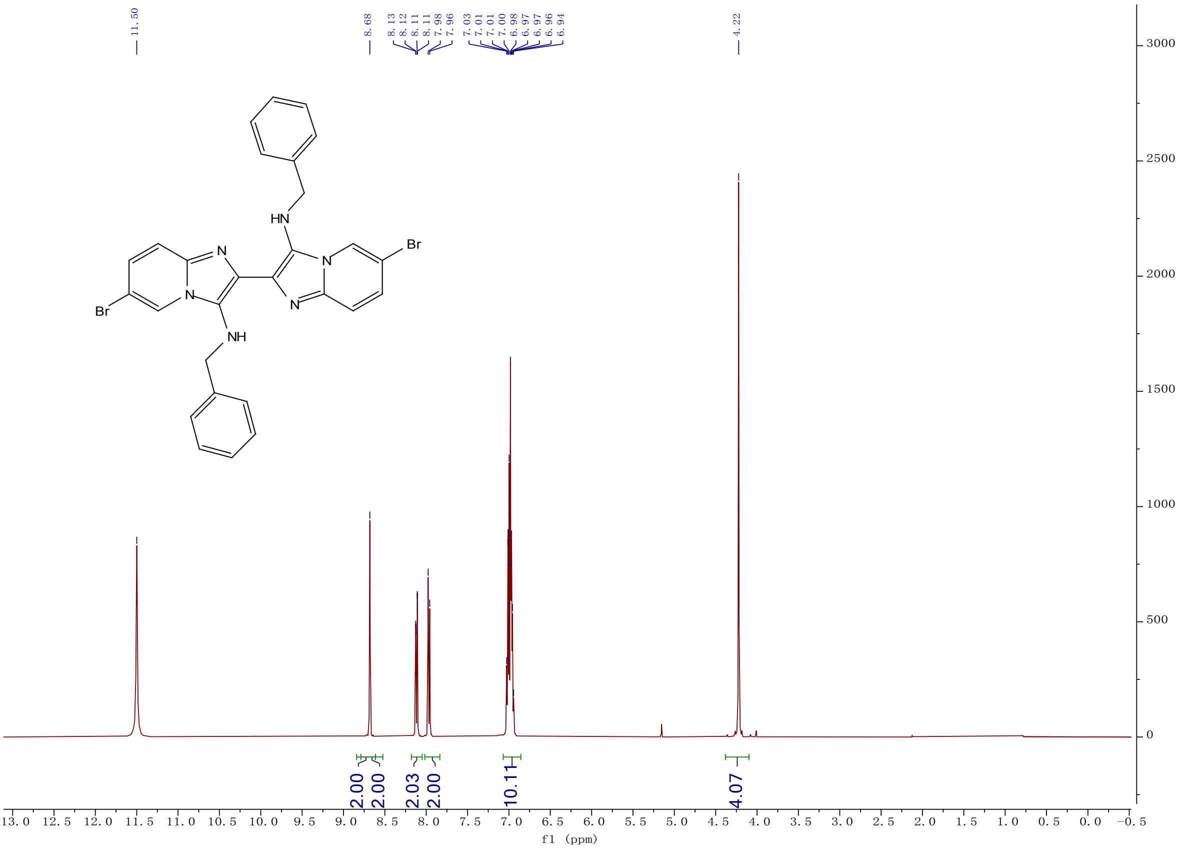

$^{13}C\{^1H\}$ NMR spectrum of **3bs** (126 MHz, TFA-d)

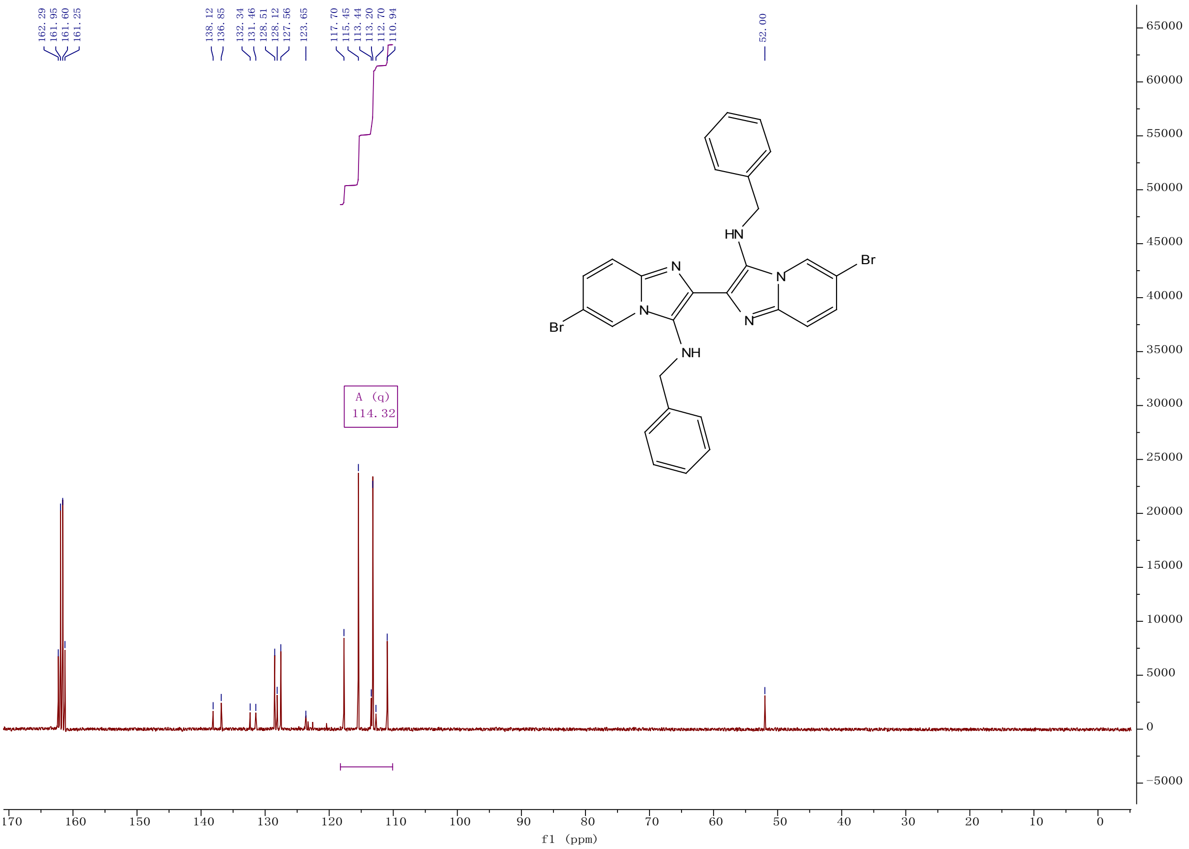

References:

[1] Rigaku-Oxford Diffraction; CrysAlisPro Oxford Diffraction Ltd, Abingdon, England V 1. 171. 36. 2. (release 27-06-2012 CN (2006)

[2] Palatinus, L. Chapuis, G., J. Appl. Cryst. 2007, 40, 786.

[3] Sheldrick, G. M. ActaCryst. 2008, A64, 112-122.

[4] Farrugia, L.J. Journal of Applied Crystallography 2012, 45, 849-854.

[5] Macrae C. F., Edgington P.R., McCabe P., Pidcock E., Shields G.P., Taylor R., Towler M., & van de Streek J., J. Appl. Cryst. 2006, 39, 453-457.

[6] Berlman, I. B. (1971) Handbook of Fluorescence Spectra of Aromatic Molecules, 2nd edn., Academic Press, New York.
